# Dual functional POGases from bacteria encompassing broader O-glycanase and adhesin activities

**DOI:** 10.1038/s41467-025-57143-8

**Published:** 2025-02-25

**Authors:** Linjiao Zhou, Uriel Ortega-Rodriguez, Matthew J. Flores, Yasuyuki Matsumoto, John Q. Bettinger, Wells W. Wu, Yaqin Zhang, Su-Ryun Kim, Thomas G. Biel, Jordan D. Pritts, Rong-Fong Shen, V. Ashutosh Rao, Tongzhong Ju

**Affiliations:** 1 Office of Pharmaceutical Quality, Center for Drug Evaluation and Research, Food and Drug Administration, Silver Spring, MD USA; 2 Facility for Biotechnology Resources, Center for Biologics Evaluation and Research, Food and Drug Administration, Silver Spring, MD USA

**Keywords:** Glycobiology, Bacteriology, Biologics, Hydrolases

## Abstract

Mucin-type O-glycans on glycoproteins are pivotal for biology and impact the quality of biotherapeutics. Furthermore, glycans on host cells serve as ligands for lectins/adhesins on bacteria for bacterium-host interactions in the colonization or attachment/invasion of bacteria. Defining the structure-function relationship of O-glycans is hindered by a lack of enzyme(s) to release sialylated O-glycans from glycoproteins. Here we show identification of endo-α-N-acetylgalactosaminidases (O-glycanases, GH101) with broad substrate specificities, termed Peptide:O-Glycosidase (POGase). In 5 POGase orthologs identified, we characterize one that releases sialylated O-glycans from glycopeptides, glycoproteins and biotherapeutics. Three peptide motifs differentiate the POGase existing in phylum *Actinomycetota* from known O-glycanases in other bacteria. While the GH101 domain classifies POGases, other domains confer the efficient enzyme activity and binding to major glycans decorating epithelial cells. The dual functional POGases encompassing broader O-glycanase and adhesin activities will facilitate the study of O-glycomics, quality assessment of biotherapeutics, and development of microbiology and medicine.

## Abstract (web summary)

This study identifies several O-glycanases, termed Peptide:O-Glycosidases (POGases), with broad substrate specificities. POGases are dual-functional, exhibiting glycan cleavage as well as adhesin-like glycan binding capabilities for diverse glycans.

## Introduction

O-GalNAc, or mucin-type O-glycosylation is a major type of glycosylation that occurs on more than 80% of proteins traversing through the secretory pathway in mammalian cells^1^. Despite the formation of the common GalNAcα-Ser/Thr/Tyr (Tn antigen) in proteins to initiate O-glycan biosynthesis by a family of ∼20 GalNAc transferases in humans^2,3^, mature O-glycans on glycoproteins from cells are diverse^2^. In mammalian systems, the Tn antigen can be extended by one or two monosaccharides to generate 8 core structures, with Cores 1-4 being the most common^4^. O-glycan core structures are often further elongated to form mature O-glycans with sialylation, fucosylation, sulfation, and other modifications^5^. O-glycans on cellular glycoproteins are known to play pivotal roles in many critical biological processes, including cell-cell and cell-matrix interactions, immunity, and more^5–7^, abiding by the biological rule of structure-function relationship. Furthermore, interactions of mucin glycans and microbiota in gastrointestinal tracts are critical for host-microbial symbioses^8,9^. Understandably, O-glycosylation is often altered in human diseases, including cancer^5,10,11^. As the repertoire of critical roles for O-glycosylation in biology, human diseases^5,10–12^, and therapeutic proteins^13,14^ has grown, further progress has been hindered by the lack of a universal enzyme to release intact O-glycans for structural analysis. This contrasts with the study of N-glycans, which can be released by commercially available enzymes, such as PNGase F/A, leading to the advancement of functional N-glycomics.

Endo-α-N-acetylgalactosaminidase (EC 3.2.1.97), also known as O-glycanase or O-glycosidase, belongs to the glycoside hydrolase family 101 (GH101), which catalyzes the hydrolysis of Core 1 disaccharide, Galβ1,3GalNAcα- from glycoproteins. At least 10 members or orthologs of the enzyme derived from different bacteria have been identified and characterized^15–24^. Most of them have a strict substrate specificity for the basic Core 1 structure. A few of them also possess limited activity to cleave Core 3, GlcNAcβ1,3GalNAcα-R disaccharides, and residual activity to Core 2, GlcNAcβ1,6(Galβ1,3)GalNAcα-R^23^. An “endo-acting O-glycanase” in a recent study was determined to be an endo-N-acetyl-β-D-glucosaminidase, a member of GH16^25^. Two other reports have shown that O-glycanase mutants can release α2,3sialylCore 1 O-glycans from glycoproteins, though with very low catalytic efficiency^26,27^. No naturally occurring enzyme has been identified to efficiently cleave sialylated O-glycans from glycoproteins. Therefore, the currently available O-glycanases, including mutants, are not applicable to functional O-glycomics studies or quality control strategies in manufacturing O-glycoprotein drugs that primarily possess sialylated O-glycans. The identification of a universal O-glycanase that can cleave a broad range of sialylated O-glycans, is an unmet need that will fill knowledge gaps in multiple scientific disciplines and improve the control of safe and effective drug products.

Here we describe the screening and identification of a previously uncharacterized endo-α-N-acetylgalactosaminidase with broad substrate specificity, termed Peptide:O-Glycosidase (POGase) that cleaves a variety of neutral and sialylated O-glycans, Core 1, Core 2, Core 3, α2,3sialylCore 1, and sialylCore 2 from glycopeptides, glycoproteins, and therapeutic glycoproteins. Furthermore, GlycanArray analysis and cell-binding assay indicate that POGases also bind to major glycans found in epithelial cells, demonstrating their potential adhesin activity. The identification of POGases has the potential to impact numerous scientific disciplines including glycobiology, biotechnology, immunology, microbiology, and medicine.

## Results

### Peptide:O-Glycosidase (POGase) orthologs were identified through screening putative O-glycanases with α2,3sialylCore 1-(4-MU)

**Fig. 1:**
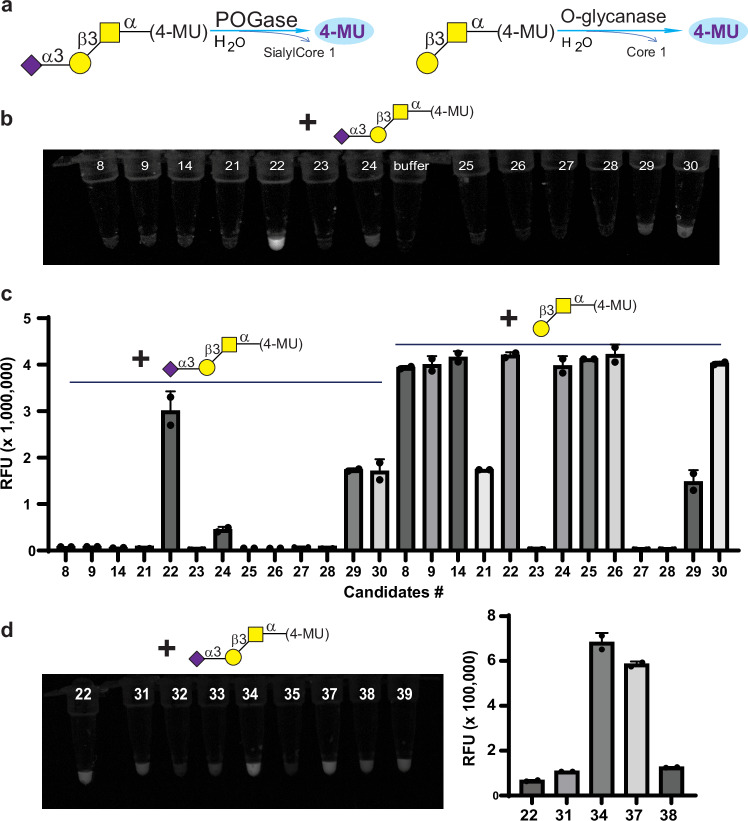
Screening POGases using α2,3sialylCore 1-(4-MU). **a** POGase screening strategy: Only POGases cleave the α2,3sialylCore 1 from α2,3sialylCore 1-(4-MU) to release fluorescence 4-MU while all O-glycanases can cleave Core 1 from Core 1-(4-MU) to release fluorescence 4-MU. **b** Representative screening results: after incubating with substrate α2,3sialylCore 1-(4-MU), four of 13 candidates in a library of 30 uncharacterized and putative O-glycanases (#1–30, Supplementary Table 1) were identified to release 4-MU which is fluorescent under UV light. **c** The candidates’ activities towards α2,3sialylCore 1-(4-MU) (left) and Core 1-(4-MU) (right) were measured respectively. Data are presented as mean values ± SD (*n* = 2). **d** Representative screening results of 16 more putative O-glycanases: an expression library of 16 more putative POGases (#31–46, Supplementary Table 1) with significant sequence similarity to the candidate #22 (termed POGase TB) was screened. Five more candidates, #31 (N01), #34 (N04), #37 (N07), #38 (N08), and #39 (N09) were identified to have significant fluorescence or activity and were shown. Activity comparison of POGase candidates: Enzymatic Reactions of 5 POGase candidates with significant activity to α2,3sialylCore 1-(4-MU) were compared. Data are presented as mean values ± SD (*n* = 2). Source data are provided as a Source Data file.

Using α2,3sialylCore 1-(4-methylumbelliferone, 4-MU), Neu5Acα2,3Galβ1,3GalNAcα1-(4-MU) as the substrate (Supplementary Fig. 1a–d), we initially selected and screened 30 uncharacterized putative O-glycanases from different bacteria (Supplementary Table 1, #1–30). These candidates were based on the EC 3.2.1.97, endo-α-N-acetylgalactosaminidase from BRENDA, a comprehensive enzyme information system. Candidate POGases cleave the sialylCore 1 O-glycans off the fluorophore and release a 4-MU (Fig. 1a), which can be visualized and then quantified by fluorescence-based approaches. The recombinant forms of enzymes were expressed in *E. coli* cells and crude bacterial lysates were screened against synthetic α2,3sialylCore 1-(4-MU), and Core 1-(4-MU) as a control. Initial evaluation with visualization under UV lights suggested that four of the recombinant enzymes, candidates 22, 24, 29, and 30, enzymatically cleaved α2,3sialylCore 1-(4-MU) and released 4-MU (Fig. 1b). Enzymatic activities of the candidates against α2,3sialylCore 1-(4-MU) were confirmed and quantified with a fluorescence reader using Core 1-(4-MU) as an additional substrate. As expected, most candidates had high activity toward Core 1-(4-MU). Clone number 22 (POGase from *Trueperella* *bernardiae*, termed as POGase TB) had the highest activity toward α2,3sialylCore 1-(4-MU) (Fig. 1b, c).

To identify more prominent POGases, a BLAST search was performed against protein sequences of POGase TB. An additional 16 putative O-glycanases from different bacteria were selected from top hits with >31% overall sequence identity to POGase TB (Supplementary Table 1, #31–46). The recombinant forms of these enzymes were expressed, and the crude lysates were screened against α2,3sialylCore 1-(4-MU). Visualization under UV lights indicated that lysates of clones #31 from *Trueperella* *pyogenes* (POGase TP), #33 from *Schaalia* *hyovaginalis* (POGase SH), and #34 from *Actinomyces* *sp.* (POGase AS), #37 from *Knoellia sp*. (POGase KS), #38 from *Bowdeniella nasicola* (POGase BN), and #39 *Knoellia remsis* (POGase KR) had significant activity on α2,3sialylCore 1-4-MU (Fig. 1d). In the 5 POGase candidates that were selected based on their relative activity, candidate #34, POGase AS, exhibited the highest observed activity as compared to all other lysates (Fig. 1d, and Supplementary Tables 1 and 2). In all cases, a control substrate, GalNAcα-(4-MU), was also tested, and all POGases had no or only residual activity toward GalNAcα-(4-MU).

### POGases contain the GH101 domain, and other domains—primary sequence alignment

**Fig. 2:**
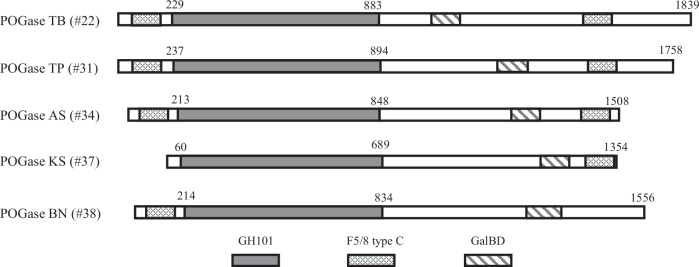
Schematic domain structures of the POGases. Alignment of the domain structures of POGases that cleave α2,3sialylCore 1 O-glycan are shown. The gray boxes correspond to the GH101 domain, while the patterned boxes indicate different types of carbohydrate-binding domains.

The primary protein sequences of POGase TB (#22), POGase TP (#31), POGase AS (#34), POGase KS (#37), and POGase BN (#38) (Supplementary Table 2) were aligned and revealed significant homology in the GH101 domain among the POGases, which covered ~600 amino acids (aa) in the N-terminal portion (Fig. 2 and Supplementary Fig. 2). The variable, relatively short N-terminal (~200 aa) and large C-terminal regions had less similarity, except for the predicted GalBD-like (Galactose-binding domain) and F5/8 type C domains, which potentially possess carbohydrate/glycan-binding activity (Fig. 2). Interestingly, unlike the other four POGases, POGase KS (#37) has a very short 63 aa N-terminal region with no predicted domain structure. In contrast, POGase TB (#22), POGase TP (#31), and POGase AS (#34) have two F5/8 type C domains, one at each terminus, and also have a GalBD domain located in between the GH101 and F5/8 type C domains. Notably, POGase BN (#38) only has one F5/8 type C domain at its N-terminus. Highly conserved peptides or motifs were observed among these POGases (Supplementary Fig. 2), which will be compared to the known O-glycanases in later sections.

### POGase AS has broader substrate specificities—characterization of kinetics

**Fig. 3:**
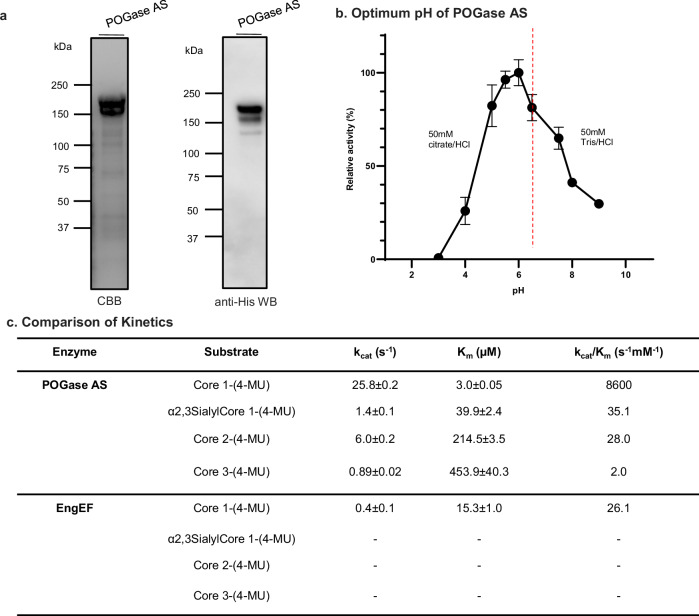
Characterization of POGase AS. **a** Expression and purification of recombinant POGase AS: The POGase AS with 6xHis tag at its C-terminus was recombinantly expressed in *E. coli* (BL21(DE3)) cells and purified with a Ni-NTA-column. SDS-PAGE with CBB staining and Western blot analysis of purified POGase AS were shown. The experiment was repeated three times independently with similar results. **b** Optimum pH: Activity of recombinant POGase AS toward α2,3sialylCore 1-(4-MU) was measured in enzymatic reactions under different pH conditions. Data are presented as mean values ± SD (*n* = 2) in two independent experiments. **c** Kinetic parameters of recombinant POGase AS: kinetics of cleavage of Core 1-(4-MU), α2,3sialylCore 1-(4-MU), Core 2-(4-MU), and Core 3-(4-MU) by recombinant POGase AS were determined. Source data are provided as a Source Data file.

POGase AS (#34) was chosen for further characterization due to its high activity against α2,3sialylCore 1-(4-MU) and ease of expression in *E. coli*. Recombinant POGase AS expressed from *E. coli* was purified using a Ni-NTA column with >85% purity (Fig. 3a). POGase AS activity had an optimum pH at 6.0, a range of 5.5–6.5 (Fig. 3b). The *K*_m_ of POGase AS towards Core 1-, α2,3sialylCore 1-, Core 2-, and Core 3-(4-MU) (Supplementary Fig. 3) was measured to be 3.0, 39.9, 214.5, and 453.9 µM, respectively (Fig. 3c and Supplementary Fig. 4). The *K*_cat_ of POGase AS towards Core 1-, α2,3sialylCore 1-, Core 2-, and Core 3-(4-MU) was determined to be 25.8, 1.4, 6.0, 0.89 s^−1^, respectively. *K*_cat_/*K*_m_ of POGase AS towards Core 1-, α2,3sialylCore 1-, Core 2-, and Core 3-(4-MU) was calculated at 8600.0, 35.1, 28.0, and 2.0 s^−1^mM^−1^, respectively. These data demonstrate that the efficiency of POGase AS to hydrolyze the substrates was highest on Core 1, but also significant on α2,3sialylCore 1, Core 2, and Core 3 structures. POGase AS cleaved α2,3sialylCore 1-(4-MU) at 180-fold slower than Core 1-(4-MU). As a control, kinetic characterization was also performed on a commercially available O-glycanase (EngEF, Cat# P0733S, NEBiolabs), and *K*_m_, *K*_cat_, and *K*_cat_/*K*_m_ could be measured only toward Core 1-(4-MU), at 15.3 µM, 0.4 s^−1^, and 26.1 s^−1^mM^−1^, respectively (Fig. 3c and Supplementary Fig. 4). Notably, POGase AS showed a *K*_cat_/*K*_m_ value approximately >300-fold higher than EngEF.

### POGase AS releases intact O-glycans from O-glycopeptides

**Fig. 4:**
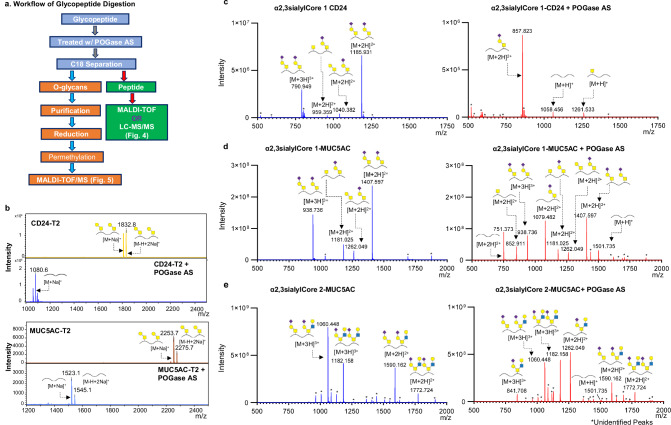
MS analysis of CD24 and MUC5AC O-glycopeptides after POGase Treatment. **a** Schematic illustration of analytical strategy: released O-glycans and peptides from glycopeptides by POGase AS were proceeded with permethylation for O-glycan analysis on MALDI-TOF and LC-MS/MS analysis, respectively. **b** Peptides from glycopeptides carrying Core 1: spectra of CD24 glycopeptides and MUC5AC glycopeptides carrying two Core 1 (CD24-T2 and MUC5AC-T2) and their corresponding peptides after POGase AS treatment were shown. **c** Mass spectra of digested CD24 glycopeptides carrying sialylCore 1: MS^1^ of α2,3sialylCore 1 CD24 before and after POGase AS treatment were shown. **d** Mass spectra of digested MUC5AC carrying α2,3sialylCore 1: MS^1^ of α2,3sialylCore 1 MUC5AC before and after POGase AS treatment were shown. **e** Mass spectra of digested MUC5AC carrying α2,3sialylCore 2: MS^1^ of α2,3sialylCore 2 MUC5AC before and after POGase AS treatment were shown. In all cases, the unmodified peptide was present after POGase release. Source data are provided as a Source Data file.

To determine if POGase AS could release intact sialylated O-glycans from glycopeptides, in contrast to O-glycosidases with no reported activity against such substrates^28^, we tested POGase AS with O-glycopeptides bearing a variety of O-glycans. MUC5AC peptide is a 16-amino acid fragment of MUC5AC, the glycopeptide bears two O-glycosites at T3 and T13. We enzymatically synthesized a series of MUC5AC O-glycopeptides bearing Core 1, α2,3sialylCore 1, and α2,3sialylCore 2 O-glycans (Supplementary Fig. 5). Additionally, we tested POGase AS activity on commercial CD24 glycopeptide substrates, with two Core 1 (CD24-T_2_) or two α2,3sialylCore 1 O-glycans (CD24-sT_2_) (Supplementary Table 3). O-glycans released after POGase AS digestion were isolated by C_18_ separation (Fig. 4a), reduced, permethylated, and analyzed by matrix-assisted laser desorption/ionization time-of-flight mass spectrometry (MALDI-TOF MS). The retained (glyco)peptides were analyzed by MALDI-TOF MS, which is suitable for glycopeptides carrying neutral glycans or, nanoC18 liquid chromatography-tandem mass spectrometry (LC-MS/MS) for sialylated glycopeptides that poorly ionize on MALDI-TOF MS (Fig. 4a). Complete release of Core 1 O-glycans by POGase AS was observed in MUC5AC and CD24 glycopeptides carrying two Core 1 O-glycans by MALDI-TOF MS analysis (Fig. 4b). CD24 glycopeptides carrying α2,3sialylCore 1 O-glycans at two glycosites (CD24-sT_2_) were observed as doubly and triply charged ions (*m/z* 1185.9 [M + 2H]^2+^ and *m*/*z* 790.9 [M + 3H]^3+^) (Fig. 4c, left panel). After treatment with POGase AS, new peaks consistent with CD24 unmodified peptide, *m*/*z* 1058.5 [M + H]^+^, and CD24 glycopeptide with one α2,3sialylCore 1 O-glycan (*m*/*z* 857.8 [M + H]^+^) appeared (Fig. 4c, right panel**)**. MUC5AC glycopeptides primarily consisted of two α2,3sialylCore 1 O-glycans, *m*/*z* 1407.6 [M + 2H]^2+^ and *m*/*z* 938.7 [M + 3H]^3+^ (Fig. 4d, left panel), and a minor species with one α2,3sialylCore 1 O-glycan at one site, the Core 1 O-glycan or Tn antigen at the other. MUC5AC glycopeptides were derived from MUC5AC-Tn_2_ glycopeptides used as substrates for enzymatic synthesis of glycopeptide (Supplementary Fig. 5). Similarly, after POGase treatment, some MUC5AC glycopeptides remained as minor species or contained two α2,3sialylCore 1 O-glycan as the minor species (Fig. 4d, right panel). More importantly, new peaks appeared corresponding to the unmodified peptide (*m*/*z* 751.4 [M + 2H]^2+^ and *m*/*z* 1501.7 [M + H]^+^) and MUC5AC glycopeptide with one α2,3sialylCore 1 (*m*/*z* 1079.5 [M + 2H]^2+^). In both studies, the glycopeptides with one Tn (CD24: *m*/*z* 1261.5 [M + H]^+^ and MUC5AC: *m*/*z* 852.9 [M + 2H]^2+^) remained (Fig. 4c, d), consistent with the observation that POGase AS does not efficiently cleave αGalNAc (Tn antigen) off glycopeptides and artificial substrates. Finally, the MUC5AC carrying two α2,3sialylCore 2 O-glycans (*m*/*z* 1773.7 [M + 2H]^2+^ and 1182.2 [M + 3H]^3+^) and a mixture of either α2,3sialylCore 2 O-glycan or α2,3sialylCore 1 O-glycan (*m*/*z* 1590.2 [M + H]^+^ and 1060.4 [M + 2H]^2+^, respectively) were also tested (Fig. 4e, left panel). Treatment with POGase AS gave only a minor change in the glycopeptide pattern: two new peaks corresponding to the MUC5AC with one α2,3sialylCore 2 O-glycan (*m*/*z* 1262.0 [M + 2H] ^2+^ and 841.7 [M + 3H]^3+^) (Fig. 4e, right panel) appeared, which could be derived from the original MUC5AC glycopeptide after release of either one α2,3sialylCore 2 O-glycan or one α2,3sialylCore 1 O-glycan. Also, one minor peak with *m/z* of 1501.7 corresponding to the unmodified MUC5AC peptide appeared. To understand the efficiency of glycan release with POGase AS, the extracted ion chromatograms (XICs) of substrate and product peaks were extracted using a mass tolerance of 0.0500 Daltons, to facilitate high-accuracy mass filtering. The percentage of the peak area of each substrate peak, and product peak are summarized (Supplementary Fig. 6). In α2,3sialylCore 1 CD24, the major product peak formed was α2,3sialylCore 1_1_-CD24, which accounted for 88.6% (Supplementary Fig. 6a), while unmodified CD24 accounted for 5.8%. In contrast, the major product peak identified in α2,3sialylCore 1 MUC5AC was the unmodified MUC5AC peak which accounted for 30%, while the α2,3sialylCore 1_1_-MUC5AC peptide accounted for 22% (Supplementary Fig. 6b). In both cases, POGase AS was not able to completely convert the glycopeptide substrate to a homogenous unmodified peptide substrate. In α2,3sialylCore 2 MUC5AC reactions, the major product identified was an α2,3sialylCore 2_1_-MUC5AC peptide which accounted for 45.7% (Supplementary Fig. 6c). A small percentage of unmodified MUC5AC was detected, however, it only accounted for 1.8%. Although the substrate peaks α2,3sialylCore 2_2_-MUC5AC (23.9%) and α2,3sialylCore 1_1,_ α2,3sialylCore 2_1_-MUC5AC (28.6%) remained after POGase AS treatment, a reduction of ~50% in both peaks were observed, indicating successful release of α2,3sialylated Core 1, and Core 2 O-glycans with preference to α2,3sialylCore 1. To further optimize the complete release of O-glycans by POGase, several factors including excess enzyme, and longer incubation periods were tested, however, full cleavage of sialylated O-glycans was not observed, most likely caused by enzyme stability issues during long incubation periods or product inhibition which will need to be further investigated. Nevertheless, unmodified peptides were generated from sialylated glycopeptides after POGase AS digestion, demonstrating its activity towards different sialylated O-glycans.

**Fig. 5:**
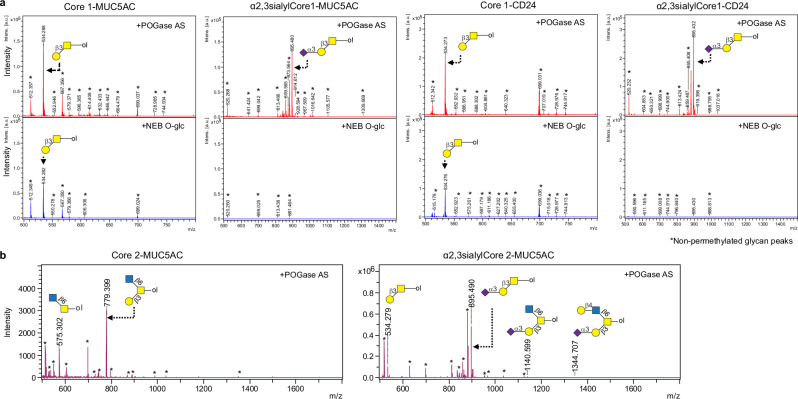
MALDI-TOF MS analysis of Released Core 1, sialylCore 1, Core 2 and sialylCore 2 O-glycans from glycopeptides CD24 and MUC5AC. **a** Core 1 and α2,3sialylCore 1 O-glycans released from MUC5AC glycopeptides and CD24 glycopeptides by POGase AS, respectively. **b** Core 2 and α2,3sialylCore 2 O-glycans released by POGase AS from MUC5AC glycopeptides. Source data are provided as a Source Data file.

To further support that POGase AS could release various intact O-glycans, from MUC5AC, and CD24 glycopeptide substrates, the O-glycans recovered from C_18_ separation were reduced, permethylated, and analyzed on MALDI-TOF MS (Fig. 5). In the glycan species derived from both the CD24 and the MUC5AC glycopeptide reactions (Fig. 4b–d), two major peaks consistent with the mass of intact, reduced, and permethylated Core 1 (*m*/*z* 534.3) and α2,3sialylCore 1 O-glycans (*m*/*z* 895.5 from MUC5AC*, m*/*z* 895.4 from CD24, respectively) were identified. (Fig. 5a top panel). When subjected to treatment with New England Biolabs (NEB) O-glycosidase (O-glc), the major O-glycan species detected in the mass spectra of Core 1 MUC5AC, and Core 1 CD24 was permethylated Core 1 (*m*/*z* 534.3) (Fig. 5a, bottom panel). Regarding the sialylated O-glycans, α2,3sialylCore 1 O-glycan was not detectable in either sialylCore 1 MUC5AC (*m*/*z* 895.5) or sialylCore 1 CD24 (*m*/*z* 895.4). Tandem mass spectra obtained by laser-induced dissociation (LID) on both *m*/*z* 534 and 895 in LIFT mode (a proprietary technique used in Bruker instruments in which LID-derived fragmentation products are initially accelerated at lower energy, then are lifted to high potential in the collision cell before mass measurement is performed^29^) confirmed that the *m*/*z* 534 peaks from Core 1 MUC5AC and CD24 were Core 1 O-glycans based on the presence of signature fragments which are only possible by mono-substitution of the GalNAc core (*m*/*z* 298) **(**Supplementary Fig. 7a, MUC5AC as a representative). The identity of the α2,3sialylCore 1 O-glycan observed in sialylCore 1 MUC5AC, and CD24 was confirmed by the presence of signature fragments corresponding to mono-substitution of the inner galactose residue (*m*/*z* 520) and both a free sialic acid residue (*m*/*z* 398) and a fragment which corresponds to a terminal sialic acid residue linked to the inner galactose residue (*m*/*z* 620) (Supplementary Fig. 7b, MUC5AC as a representative). MALDI-TOF MS analysis of O-glycans released from α2,3sialylCore 2 MUC5AC (Fig. 4e) demonstrated two O-glycan peaks at *m*/*z* 779 and 575 (Fig. 5b left panel) that correspond to a Core 2 O-glycan and a Core 6 O-glycan, respectively. The latter was unanticipated because GCNT2 is responsible for deriving a Core 2 O-glycan from Core 1, and appears to work on Tn antigen. MALDI-TOF MS analysis of O-glycans released from α2,3sialylCore 2 MUC5AC revealed evidence of mixed Core 1 and Core 2 oligosaccharides which included Core 1 (*m*/*z* 534.3), α2,3sialylCore 1 (*m*/*z* 895.5) and two sialylCore 2 O-glycans with or without a galactose residue on the β1,6GlcNAc branch (*m*/*z* 1140.6 and *m*/*z* 1344.7, respectively) (Fig. 5b, right panel). Tandem mass spectra in LIFT mode obtained from *m*/*z* 779 confirmed the identity of the Core 2 O-glycan by distinctive fragments *m*/*z* 302, and *m*/*z* 284 that are only possible by di-substitution of the reduced GalNAc residue, and *m*/*z* 543 which is a fragment formed by the loss of a terminal galactose residue leaving a terminal GlcNAc linked to the reduced GalNAc core (Supplementary Fig. 7c). Fragmentation of the possible Core 6 O-glycan at *m*/*z* 575 (Supplementary Fig. 7d) confirmed the identity of this glycan by the presence of *m*/*z* 316 and *m*/*z* 298 that are only possible by mono-substitution of the reduced GalNAc core, and the fragment *m*/*z* 282 which is consistent with a terminal GlcNAc residue. Evidence of other potential cross-ring fragments at *m*/*z* 413 and *m*/*z* 429 were also present (Supplementary Fig. 7d). These data suggest that the GCNT2 can synthesize Core 6 structures in vitro, in addition to Core 2 structures. We attempted to confirm the two sialylCore 2 peaks identified (*m*/*z* 1140.6 and *m*/*z* 1344.7, Fig. 5b, right panel), however, the peak with *m*/*z* 1344 was very low in abundance, and due to poor precursor ion isolation, only the identity of the *m*/*z* 1140 peak corresponding to the sialylCore 2 that lacks a galactose on β1,6GlcNAc was confirmed by fragmentation patterns observed in the tandem mass spectra (Supplementary Fig. 7e). The combination of fragments at *m*/*z* 765, 881, 506, and 543 was used to confirm the branched structure of this sialylCore 2 O-glycan (Supplementary Fig. 7e). Furthermore, we also identified a fragment at *m*/*z* 620 (Supplementary Fig. 6e) consistent with the mass of a terminal sialic acid residue linked to an internal galactose residue which was also present in the α2,3sialylCore 1 peak, which further supports that the sialic acid residue was present on the β1,3Gal branch.

Together, these data confirm that POGase can release Core 1, Core 2, α2,3siaylCore 1, and α2,3siaylCore 2 O-glycans, as well as Core 6 O-glycans.

### POGase AS releases intact O-glycans from O-glycoprotein and biotherapeutics

**Fig. 6:**
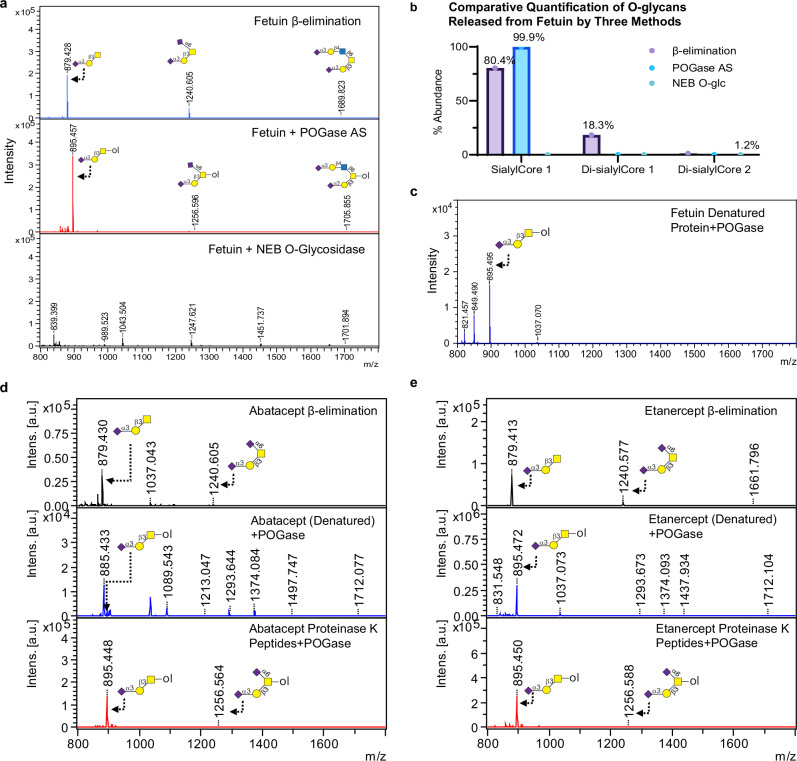
Release of sialylated O-glycans from bovine fetuin and protein drugs etanercept and abatacept. **a** O-glycans from fetuin peptides by β-elimination, POGase AS, and O-glc digestions: Top panel: O-glycans released from glycopeptides of fetuin by β-elimination (unreduced glycans); Middle panel: POGase AS released O-glycans with a 16 Da increased mass shift (reduced glycans); Bottom panel: NEB O-glycosidase (O-glc) treatment of fetuin did not yield any sialylated O-glycan peaks. **b** Comparative quantification of released O-glycan species by three different methods from (**a**). **c** Mono-sialylCore 1 O-glycans (*m*/*z* 895) released from denatured fetuin by POGase AS digestion. **d**, **e**: O-glycans from protein drugs released by POGase AS: glycopeptides of abatacept (**d**) and etanercept (**e**) treated by POGase AS yielded two sialylated O-glycan peaks consistent with α2,3sialylCore 1 and a minor di-sialylCore 1 peak (*m*/*z* 895, *m*/*z* 1256, respectively) as compared to the corresponding peaks (*m*/*z* 879, *m*/*z* 1240, respectively) from the β-elimination (unreduced glycans). The O-glycans from denatured proteins treated with POGase AS only yielded mono-sialylCore 1 (*m*/*z* 895). Source data are provided as a Source Data file.

To assess the potential for POGase AS to release glycoprotein-derived O-glycans, bovine fetuin, a frequently used model glycoprotein was selected. In parallel, O-glycans were released with POGase AS, NEB O-glycosidase, and with our optimized one-pot glycomic method in which O-glycans are released by β-elimination during permethylation of short O-glycopeptides obtained from digestion of the protein backbone with proteinase K^30^. Consistent with other reports, three major O-glycan structures were obtained by β-elimination of fetuin (Fig. 6a, top panel) which are unreduced forms of permethylated mono-sialylCore 1 (*m*/*z* 879.4), di-sialylCore 1(*m*/*z* 1240.6), and di-sialylCore 2 (*m*/*z* 1689.8)^30,31^. The relative abundance of the three O-glycans was calculated based on peak intensity and is presented in Fig. 6b**(**sialylCore 1: 80.4%; di-sialylCore 1: 18.3%; di-sialylCore 2: 1.2%). O-glycans released by POGase AS and NEB O-glycosidase were reduced, introducing a mass increase of 16 Da, that is only present in enzymatically released oligosaccharides to differentiate them from β-eliminated O-glycans if any contaminating O-glycopeptides remain after C_18_ separation. In fetuin O-glycopeptides treated with POGase AS (Fig. 6a, middle panel), the major O-glycan identified was reduced mono-sialylCore 1 (*m*/*z* 895.5) which accounted for 99.9% of the glycan profile, and two low abundant peaks consistent with reduced di-sialylCore 1 (*m*/*z* 1256.6), and di-sialylCore 2 (*m*/*z* 1705.9) were present, however, both of these O-glycan species accounted for less than 0.01% each. These data suggest that POGase AS preferentially releases mono-sialylCore 1 O-glycans, with limited activity against di-sialylCore 1, and di-sialylCore 2 O-glycans, which is consistent with similar observations made with synthetic O-glycopeptides, in which POGase AS could not efficiently release sialylCore 2 O-glycans from MUC5AC. Nevertheless, some residual activity on these di-sialylated O-glycans was observed (Figs. 4–6). In contrast, in fetuin treated with NEB O-glycosidase, no sialylated Core 1, or Core 2 O-glycans were obtained (Fig. 6a, b).

The capability for POGase to release O-glycans from fetuin glycoproteins without the requirement for proteinase K was also assessed (Fig. 6c). Briefly, fetuin was processed with materials from a commercial filter-aided sample preparation (FASP) kit, and the retained glycoprotein was digested with POGase AS. In contrast to Fig. 6a, only mono-sialylCore 1 (*m*/*z* 895) was identified in the mass spectra, suggesting that disintegration of the peptide backbone facilitates partial release of di-sialylCore 1 (*m*/*z* 1256) and di-sialylCore 2 (*m*/*z* 1689). We also assessed the capability of POGase AS to release O-glycan species from two therapeutic proteins: etanercept and abatacept^14,32^. Both proteins were processed with materials from the FASP kit. In parallel, the same glycoproteins were processed for β-elimination as described in our previous study^30^, and another set of glycoproteins were digested with proteinase K, and the O-glycopeptides were then treated with POGase. In abatacept and etanercept, only two O-glycan species were identified from β-elimination reactions, which included mono-sialylCore 1 (*m*/*z* 879.4) and di-sialylCore 1(*m*/*z* 1240.6) (Fig. 6d, e, top panels), which is consistent with the observations made in our previous study with the same glycoproteins^30^. POGase treatment of denatured etanercept and abatacept resulted in a single O-glycan peak at 895 *m*/*z*, consistent with the mass of reduced mono-sialylCore 1 O-glycan (Fig. 6d, e, middle panels), however, in abatacept, other non-specific peaks were present, suppressing the O-glycan peak. In comparison, O-glycan release from O-glycopeptides of abatacept and etanercept generated by proteinase K resulted in much cleaner O-glycan profiles, and enhanced release of the mono-sialylCore 1 (*m*/*z* 895) in abatacept was observed (Fig. 6d, e, bottom panels). In addition to mono-sialylCore 1, POGase AS was capable of partially releasing di-sialylCore 1 O-glycans (*m*/*z* 1256.6) in both therapeutic glycoproteins (Fig. 6d, e, bottom panels). Tandem mass spectra in a LIFT mode could only be recorded for the peak corresponding to *m*/*z* 895 of all glycoproteins (Supplementary Fig. 8). The combination of *m*/*z* 520.1 and 298.0 peaks supports that the *m*/*z* 895 peak corresponds to the α2,3sialylCore 1 O-glycan, as these fragments are only possible by mono-substitution of the reduced GalNAc and the internal galactose residue. No evidence of di-substitution was observed to confirm that the major O-glycan species released was linear trisaccharide with an α2,3-linked sialic acid attached to the galactose instead of the branched glycoform which bears α2,6sialylation of the GalNAc core. Tandem mass spectra were not possible to obtain on the di-sialylCore 1 or di-sialylCore 2 peaks from POGase AS-treated samples due to the low peak abundance of the precursor ion. Together, these data further demonstrate that POGase AS can release intact sialylated O-glycans from glycoproteins and biotherapeutics.

We attempted experiments on release, recovery, and MS analysis of O-glycans from more complex samples such as colon cancer cell extracts with digestion by POGase AS, however, we could not obtain clean O-glycan spectra. This is most likely due to the complexity of samples in nature, and importantly the limited accessibility of POGase AS to cluster O-glycans on mucin-domains of glycoproteins and mucins from cell extracts, which is a common obstacle for O-glycomics. Several strategies such as combining digestions of cellular glycoproteins with proteases, O-glycopeptidases, and mucinases, and importantly generating a universal POGase are currently taken to develop a feasible biotechnology for O-glycomics.

### Three distinctive peptide sequences, Motif-1, Motif-2, and Motif-3, differentiate POGases from known O-glycanases—sequence comparison of POGases to the known O-glycanases

The above results demonstrated that POGases have broader substrate specificity than the known O-glycanases, indicating that structural differences exist between them. While these proteins all belong to the GH101 family, alignments of the sequences spanning GH101 domains of all 5 POGases against the 5 known O-glycanases revealed significant differences in three peptide motifs: Motif-1, AWGWMNQ in POGases versus GWD/NWLDN in O-glycanases; Motif-2 WANEEAY in POGases versus WSAEKDY in O-glycanases; and Motif-3, YSAWAWV/IEI in POGases versus YSLYLNTET (EngEF) or YAVYVGVDNR [EngBF, SpGH101(Q8DR60)] in O-glycanases (Supplementary Table 4, Supplementary Figs. 2 and 9). Motif-1 and -2 are within the predicted GH101 domain, and Motif-3 is C-terminal to the GH101 domain, but within other domains. These differences may account for the differences in substrate specificity between POGases and known O-glycanases. Additionally, compared to the known O-glycanases, the POGases are generally larger proteins with longer and less conserved sequences in their C-terminal regions. Interestingly, C-terminal regions of POGases contain one or two predicted domains, such as F5/8 type C domains, GalBD, or even sialidase-like domains with carbohydrate-binding properties. However, known O-glycanases often only possess one predicted carbohydrate-binding domain. Most POGases, except for POGase KS (#37), also contain an extra glycan-binding domain at their N-termini before the GH101 domain.

### α2,3SialylCore 1 and other O-glycan moieties can be docked in POGase AS— structural modeling

**Fig. 7:**
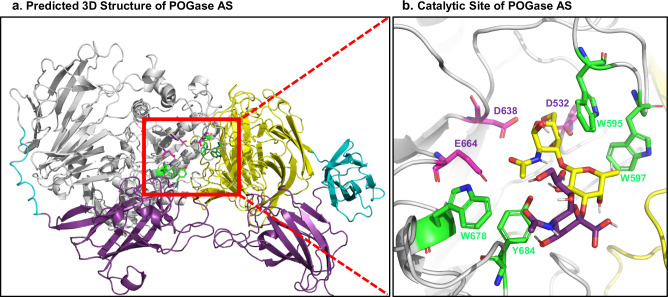
Structural modeling of POGase AS. **a** POGase AS from AlphaFold2 with each domain is highlighted: GH101 domains 1–5 are in white, GalBD domains (GH101 domain 6) are in yellow, the F5/8 type C domains are in purple, and unstructured or unannotated domains are in cyan. **b** Catalytic channel of POGase AS: the overall structure of the active site of POGase AS with α2,3sialylCore 1 in the binding pocket is focused. Residues W595, W597, W678, and Y684 forming the substrate-binding pocket are shown in green, and residues D532, D638, and E664, which compose the DDE catalytic triad, are shown in magenta.

Structural modeling of POGase AS (#34) indicated complex structures composed of individually assembled domains with the GH101 (Fig. 7a), and the overall 3D structure of POGase AS (Supplementary Movie 1). Focusing on the predicted catalytic site within the GH101 domain revealed a channel consisting of two motifs (green) generating the active site lid to allow for proper orientation of the glycan moieties in the active site, before being cleaved by the DDE catalytic triad (magenta) (Fig. 7b), which is also found within the active site of known O-glycanases^33,34^. Two motifs make up the glycan coordination channel: AWGWMNQ (Motif-1) and WANEEAY (Motif-2) (Supplementary Table 4). In the WWWY channel, two W residues in Motif-1, and the W and Y residues in Motif-2 seem to be involved in glycan positioning and binding as suggested by previous studies^35^. In the known O-glycanases, these motifs are altered to GWDWLDQ (Motif-1) and either WSAEKDY, WGGDPAY, or WAADLTY (Motif-2) (Supplementary Table 4, Supplementary Fig. 9). Previous studies have reported that the dual tryptophan active site lid residues, W864 and W866 in Motif-1 in EngBF, play a critical role in substrate recognition and properly positioning glycans for catalysis^33,35^. The biggest difference between these motifs in POGases versus O-glycanases is that in 3D space there is a charge differential: zero charge in Motif-1 and two negative charges in Motif-2 for POGases, while there are 1 or 2 negatively charged residues in Motif-1 and one negatively charged residue in Motif-2 for known O-glycanases. The presence of these charged residues in the known O-glycanases likely contributes to a narrower substrate pool than the POGases. Furthermore, a highly conserved leucine (L867, numbering in EngBF) residue in known O-glycanases, was found to be substituted with a highly conserved methionine residue (M598, numbering in POGase AS) within Motif-1 in POGases (Supplementary Table 4). It is therefore possible that the side-chain differences of M598 and L867 in combination with the charge status of Motif-1 and 2 in POGase AS, may alter the conformational dynamics of the glycan stabilization channel and play a critical role in the recognition of sialylated O-glycans. In terms of the role of the M/L substitution’s effect on activity, the substitution seems to alter the area of hydrophobic interactions within the hydrophobic core of the enzymes. Evolutionary coupling analysis reveals that the M/L substitution will also induce a change in the residue that the M/L will interact within the hydrophobic core, namely I/F respectively (Fig. 7, Supplementary Movies 1 and 2). The M/I interaction interface has less area for the hydrophobic interaction to occur (less contact points) than the L/F pair, again suggesting a possible explanation for how the active site’s flexibility is altered to allow for more complex (sialylated and branched) O-glycans. Together, the identity of these motifs in POGases likely allows for activity towards a larger pool of substrates leading to its broader substrate specificity.

To further support the structural assessment, Core 1 and Core 3 O-glycans were computationally docked to the POGase AS (Supplementary Fig. 10) with two different modeling approaches, AutoDock Vina (Supplementary Fig. 10a, b) and Vina-Carb (Supplementary Fig. 10c, d). All O-glycan moieties tested had the αGalNAc proximally oriented toward the DDE catalytic triad. When docking glycans into the active site of POGase AS, considering the intramolecular forces of Core 1 and Core 3 O-glycans had no effect on the orientation or binding energy of the docked glycan. Comparison of the two algorithms reveals similar poses and binding energy for simple, disaccharide glycans, except for α2,3sialylCore 1 (Supplementary Table 5). For α2,3sialylCore 1, AutoDock Vina alone could not accurately orient the glycan in the active site, however, after incorporating an energetic penalty for suboptimal glycan torsion angles using Vina-Carb, the glycan retained a similar position to not only the disaccharide O-glycans docked in this study but a T antigen (Core 1) analog found in a spGH101 domain that has previously been experimentally derived^35^. The distance of the anomeric carbon to the DDE catalytic triad in any of the docked structures presented here does significantly differ from an experimentally derived structure of a T antigen analog (Core 1) bound to an experimentally derived GH101 domain (PDB ID:5a56) (Supplementary Fig. 11), suggesting that the computationally predicted poses are conducive towards catalysis.

We also attempted to dock the Core 2, GlcNAcβ1,6(Galβ1,3)GalNAcα to the POGase AS catalytic site using AutoDock Vina and Vina-Carb, however, no reasonable pose was predicted. In contrast, the Core 2 glycan was able to be reasonably docked to one of the POGase orthologs, POGase KS (#37), which will be further investigated in a separate study. Despite attempts to dock α2,6sialylCore 1 into the active site, no reliable results could be observed, which could explain the observation of the limited activity of POGase AS on di-sialylCore 1 O-glycans. It should be noted that the computational prediction of substrate binding to predicted protein structures is reliant on the accurate prediction of amino acid side-chain orientations as well as the conformational dynamics of enzyme active sites. While it has been suggested that AlphaFold has appreciable accuracy in the prediction of amino acid side-chain orientations, experimental validation of computational predictions and resolution of discrepancies with the experimentally determined substrate binding of POGases is still needed^36^. Nonetheless, our molecular docking experiments yield valuable insights into the potential determinants of expanded substrate preferences in POGase AS; such as differences in the active site charge differential, hydrophobic area, and flexibility.

### POGases as potential adhesins bind to glycans

**Fig. 8:**
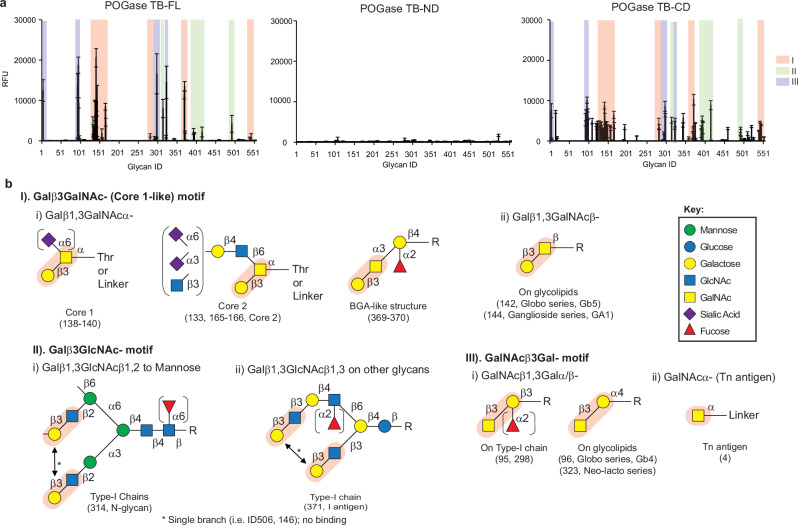
Glycan-binding profiles. **a** GlycanArray Analysis: the POGase TB-FL, -ND, and -CD were analyzed on the nCFG GlycanArray, and the binding signal (relative fluorescence units, RFU) were measured and summarized on the bar graph (error bars, ±1 SD of four replicates, after eliminating the highest and lowest fluorescence reading from 6 glycan spots). **b** Shared Glycan determinants: Glycan motifs recognized by both POGase TB-FL and its -CD were grouped based on the Motifs (I, II, III) in (**a**). Source data are provided as a Source Data file.

POGases are large proteins with 1500–2000 aa residues as compared to known O-glycanases (1000–1300 aa). The POGase KS (#37) is an exception with 1354 aa. Although POGases and O-glycanases share the GH101 domain at their N-terminal regions, the POGases have very large (~700–1000 aa) and diverse C-terminal regions containing other domains such as F5/8 Type C domain and GalBD (Fig. 2). The F5/8 Type C domain, also known as discoidin domain, is found in the GH29 and GH32 family of glycoside hydrolases involved in glycan binding^37,38^. These features imply that the POGases may have additional function(s), such as carbohydrate-binding as adhesins. POGase TB was chosen for GlycanArray analysis since it was the first identified POGase ortholog, and its full-length (FL) and domain substructures, ND (N-terminal Domain) and CD (C-terminal Domain) were expressed and obtained (Supplementary Fig. 12a) to test their potential glycan-binding activity. Binding activity was tested at 4 °C rather than 25 °C to limit the possible cleavage of the O-glycans by POGases. POGase TB-ND had little to no enzymatic activity to both substrates, Core 1 and α2,3sialylCore 1-(4-MU), while POGase TB-CD had no activity as expected (Supplementary Fig. 12b). Loss of enzymatic activity of POGase TB-ND suggests that the overall fold or cooperativity between the two domains has been lost. GlycanArray data demonstrated significant and specific glycan-binding activities among the POGase TB-FL and POGase TB-CD, but not the POGase TB-ND (Fig. 8a, and Supplementary Material). The consensus of their glycan-binding determinants is mainly the terminal β3Gal- and β3GalNAc- in three glycan motifs. They are: (I) Galβ3GalNAc- motif in Core 1 (including α2,6sialylCore 1), Core 2 (including sialylCore 2) O-glycans, and in BGA-like epitope (Galβ1,3GalNAcα1,3-), as well as in Galβ1,3GalNAcβ1,3 in glycolipids; (II) Galβ3GlcNAc- motif in bivalent Type-I chains (Galβ1,3GlcNAcβ1,2Manα-) of N-glycans and I antigen in O-glycans and others; and (III) GalNAcβ3Gal- motif in terminal GalNAcβ1,3Galα/β- found in glycolipids of Globo-series. Interestingly, POGase TB-FL and POGase TB-CD are also bound to Tn antigen (GalNAcα1-*O*-linker) as a truncated O-glycan (Fig. 8b, and Supplementary Material). Generally, sialylation of those motifs abolished the binding by POGase TB-FL and POGase TB-CD. Importantly these glycan motifs are mostly found in O-glycans, N-glycans, and glycolipids (Fig. 8b, and Supplementary Material) that are commonly expressed on mammalian epithelial cells. Overall, POGase TB displayed higher binding signals than POGase TB-CD to their shared glycans, while POGase-CD appeared to have a broader specificity, such as both terminal GalNAcα- and GalNAcβ-Linker (ID4 and 15) with similar affinity/signal (Supplementary Material). Since POGase is expected to be expressed as a whole, the broader specificity of POGase-CD may not necessarily be biologically relevant and, thus should not be over-interpreted. Nevertheless, this data implies that the ND in the full-length POGase may have some indirect impact on glycan binding or recognition probably due to the conformational differences between the CD within the full-length protein versus the individual CD alone. Taken together, these data indicate that POGase TB is a general glycan-binding protein for O-glycans, the Type-I chain of N-glycans, and glycolipids.

**Fig. 9:**
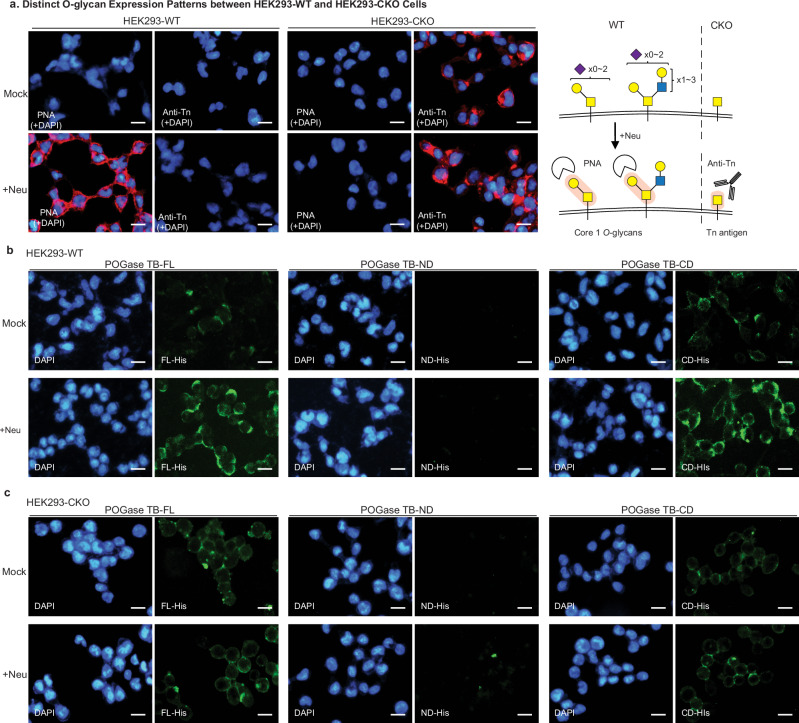
Cell-binding by POGase. Wild-type HEK293 cell line (HEK293-WT), and its isogenic line with *Cosmc*-mutation (HEK293-CKO) were cultured on 4-well chambered slides for 24 h, and then desialylated with neuraminidase (+neu), fixed, stained with **a** PNA and anti-Tn mAb; **b**, **c** with recombinant 6xHis-tagged POGase TB-FL, -ND, and -CD at 50 µg/mL and corresponding fluorescently labeled streptavidin and secondary antibodies, respectively. DAPI was used to stain nuclei. Images were collected by the fluorescence microscopy at 20× objective. Representative images were shown (*n* = 4). Bars, 20 μm. The experiment was repeated by immunofluorescence and flow cytometry analyses of a different set of cell lines independently with similar results.

To further investigate the glycan-binding or adhesin activity of POGase, an experiment of cell-binding by POGase was carried out. Human embryonic kidney cells (HEK293-WT) express Core 1-based O-glycans, mainly sialylCore 1 and sialylCore 2 on their membrane proteins as confirmed by PNA staining after desialylation (Fig. 9a, HEK293-WT). HEK293 cells with *Cosmc* gene mutation (HEK293-CKO) were expressed Tn antigen only^39^ (Fig. 9a, HEK293-CKO). Both POGase TB-FL and -CD weakly bound to HEK293-WT cells, but strongly bound to the cells after desialylation (Fig. 9b). They minimally or moderately bound to HEK293-CKO cells without much effect of sialylation (Fig. 9c, HEK293-CKO) likely through recognizing the Tn antigens uniformly expressed on the cells. POGase-ND did not show any binding to either HEK293-WT or HEK293-CKO cells. These results were consistent with the GlycanArray data (Fig. 8). These results indicated that both POGase TB-FL and -CD specifically bind to the HEK293 cells in an O-glycan-dependent manner as HEK293 cells mainly express complex N-glycans with Type-II, but not Type-I chains and little gangliosides^40^.

Taken together, the GlycanArray and cell-binding data demonstrate that POGase TB has glycan-binding activity and potentially functions as an adhesin in addition to its O-glycan-cleaving activity.

### POGases mainly exist in *Actinomycetota*—evolutionary analysis

**Fig. 10:**
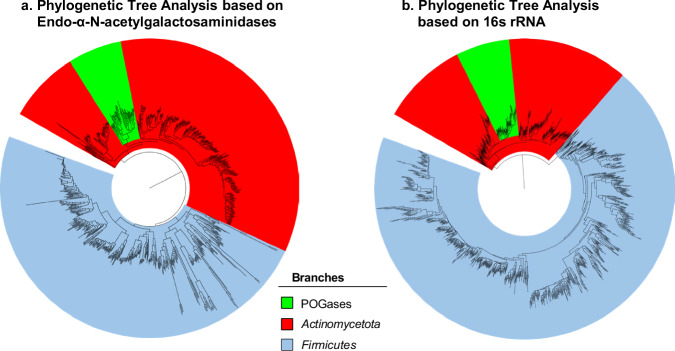
Evolutionary History of GH101s. **a** Phylogeny of Endo-α-N-acetylgalactosaminidases as a marker gene with the WAG + F + I + G4 substitution model in IQ-TREE. Maximum Likelihood Phylogenetic tree showing that POGases are all evolutionarily related and are expressed in *Actinomycetota*. Other GH101 domains containing GHs are evolutionarily distinct and are expressed in *Firmicutes*. **b** The evolutionary history of the species expressing the GH101s was also generated but using 16s rRNA nucleotide sequences from *Actinomycetota* and *Firmicutes* with the SYM + I + G4 substitution model in IQ-TREE. The evolutionary history of the proteins matches that of the species in which it’s expressed, indicating the proteins evolved with the species as a niche was found.

In the phylogenetic analysis, the group containing the POGase orthologs (POGase TB, POGase TP, POGase AS, POGase KS, and POGase BN) was analyzed and all putative POGase sequences obtained from the NCBI database were determined to fall within a monophyletic clade, appearing to be derived from *Actinomycetota* (Fig. 10a). The GH101 family of endo-α-N-acetylgalactosaminidases (ENGs) seems to have independently evolved in *Firmicute* bacteria and *Actinomycetes* (Fig. 10b), a pattern also observed when comparing the 16 ribosomal RNA sequences of these phyla (Fig. 10a), corroborating that these proteins independently evolved in these phyla based on their niche. Interestingly, most species of *Actinomycetota* have an ENG present, whereas many of the *Firmicutes* do not.

*Firmicut*e-derived ENGs contain a much higher diversity within their sequences, suggesting that the ENGs expressed in these bacteria serve a more direct role (substrate-specific), whereas the ENGs in *Actinomycetes* may serve a more generalized role. This seems to be supported by the broader substrate pool of the POGases compared to other ENGs, such as EngBF, EngCP, or EngEF, all of which are expressed by some members of the *Firmicutes*. Deeper analysis reveals alterations in Motif-1 in the active site identified via structural analysis, with changes in the charge states, architecture, and predicted flexibility likely leading to distinct differences in substrate recognition and, thus specificity. Evolutionary coupled (EC) analysis has identified residues that may have a role in this substrate recognition, many of which match the structural analysis using the POGase AS model. Many of these EC results suggest that in addition to the substitutions in the motifs themselves, the hydrophobic core of the proteins seems to have a role in the substrate differential noted in the enzymes. M/I in POGase AS and L/F were identified via EC analysis (Supplementary Movie 2) which helped support the structural modeling results that were observed, suggesting that there may be more area for hydrophobic interactions to occur in the core of the protein, restricting flexibility in substrate limited O-glycanases (Supplementary Movie 2).

## Discussion

We identified five previously uncharacterized O-glycanases with significant activity to hydrolyze sialylated O-glycans, termed POGases. Further characterization of one of the POGases, POGase AS from *Actinomyces sp*., showed its capability to enzymatically hydrolyze Core 1, sialylCore 1, Core 2, sialylCore 2, and Core 3 O-glycans from artificial substrates, glycopeptides, and glycoproteins. Moreover, the POGase tested was found to function as adhesins as demonstrated by binding to O-glycans and Type-I chains of N-glycans on GlycanArray and cells after neuraminidase treatment likely through their C-terminal domains of the proteins. Interestingly, the C-terminal domain/portion of POGases is also required for efficient enzymatic activity. The POGase orthologs seem to exist in a restricted bacterial clade derived from *Actinomycetota*. This report demonstrates that naturally occurring POGases in a specific clade of bacteria have dual activities: hydrolyzing sialylated O-glycans and functioning as adhesin molecules.

The POGases, a previously uncharacterized class of O-glycanases, have much broader substrate specificities with distinctive properties compared to known O-glycanases. In addition to Core 1 and Core 3 disaccharide, its substrates include (1) sialylCore 1 O-glycans, including both α2,3 and α2,6sialylCore 1, and di-sialylCore 1; (2) Core 2 trisaccharide, galactosylated and sialylated Core 2 O-glycans; and (3) Core 3 O-glycans. All these encompass the major sialylated O-glycans of animal cells in nature. Furthermore, POGase AS has much higher activity than EngEF towards Core 1 (*K*_m_ of 3.0 µM versus 15.3 µM) and Core 3 (*K*_m_ of 453.9 µM versus un-measurable) (Fig. 3). Around 10 known O-glycanases (EC 3.2.1.97) in the GH101 family identified in several bacteria have a strict substrate specificity, acting only on Core 1, and, in a limited number of cases, Core 3 disaccharides with residual activity^15–24^. None of them have any activity towards sialylated O-glycans. Site-directed mutagenesis, protein engineering, and screening were used to generate O-glycanases capable of cleaving α2,3sialylCore 1^26,27^. Wardman et al. generated a Q868G mutant of SpGH101 that releases the α2,3sialylCore 1 from a chromogenic substrate and glycoproteins, as well as whole cells^26^. However, both the endo α-galactosaminidases from *T. nexilis* (Tn2105)^24^ and EngCP from *C. perfringens*^21^ which have a G868 residue were found to lack sialylCore 1 cleaving activity, indicating the amino acid residues in other regions also played roles in the substrate specificity. The POGases have corresponding amino acid residues with small side chains (A/V/T). Recently, Hansen et al. generated an E1294A EngBF variant, beyond the GH101 domain, and showed the release of α2,3sialylCore 1 from fetuin^27^. The POGases have an Ala residue corresponding to the E1294 in EngBF, suggesting structures in other domains beyond GH101 may also contribute to substrate specificities of this family of enzymes. In comparison to the naturally occurring POGases identified in this work, the mutants of O-glycanases displayed only residual activity in releasing α2,3sialylCore 1 O-glycans (*K*_m_ was not measurable)^26,27^. Furthermore, other substrates were not tested for those mutants.

The POGase is a promising tool for enzymatic release of intact sialylated O-glycans from glycoproteins for functional O-glycomics and quality assessments of therapeutic glycoproteins (Fig. 6), especially CHO-manufactured protein drugs which are predominantly glycosylated with Core 1-based sialylated O-glycans^14^. While the intensity of di-sialylated Core 1 and Core 2 O-glycans released by POGase AS was lower than the β-eliminated counterparts, further investigation of undescribed POGase enzymes or mutagenesis may yield an ideal candidate with broad substrate specificity and enhanced efficiency for release of di-sialylCore 1 and other sialylated O-glycan species. The disintegration of the protein is a key step in the O-glycan release process by POGase AS which made release of di-sialylated O-glycans possible, most likely by removing steric hindrance from the peptide backbone and facilitating cleavage site accessibility. POGase AS is also a large protein with an estimated size of ~200 kDa. Identifying a smaller functional domain might also facilitate accessibility for the release of O-glycans directly from undenatured glycoproteins and cell substrates.

POGases have both distinctive and shared sequence motifs compared to known O-glycanases. The crystal structure of an O-glycanase, EngBF, was resolved and its substrate-binding domain was identified^33^. In addition, several studies have investigated the sequences, motifs, and amino acid residues in the known O-glycanases required for enzymatic activity^22,34,41^. POGases share most of the conserved sequences, motifs, and conserved amino acid residues that are critical for activity within their GH101 domain. Those include the motifs: VDWQD, EGHD, V/IHV/IN, RFLxN, and the DDE catalytic triad^41^ (Supplementary Fig. 2 and Supplementary Fig. 9). Not surprisingly, POGases also have distinctive sequence motifs within the GH101 domain: POGases contain AWGWMNQ (Motif 1) and WANE/DEAY (Motif 2), whereas the known O-glycanases contain A/GWDWLDN (Motif 1) and WSAEKDY (Motif 2 of EngEF) (Supplementary Table 4, Fig. 7, and Supplementary Fig. 9) among others beyond the GH101 domain. Interestingly, EngPA has hybrid sequences of POGases and O-glycanases within the Motifs: AWDWMEQ (Motif-1), WANDENY (Motif-2), and HSLWAWVQI (Motif-3), however, its activity on sialylated O-glycans was not detected^23^.

Based on structural modeling, the POGases seem to use these two motifs to help stabilize glycans within their active site. The residues that help actively stabilize the glycans are three W residues and one Y residue found in these two motifs, with the other residues likely allowing for glycan specificity through charge state and flexibility. A comparison of the interface created by these motifs to the conjugate motifs created in other O-glycanases indicates the charge state in this cleft seems to be altered based on the change to the motifs from EngEF. This could begin to explain how the POGases are able to recognize a broader substrate pool, especially sialylated O-glycans. Molecular docking also consistently reveals that the various O-glycan moieties are consistently docking between the two motifs (Fig. 7, and Supplementary Fig. 10), with the DDE catalytic triad embedded within the same cleft for POGase AS, which is consistent with what had been found in other O-glycanases^33^. Because the models are in the apo confirmation, CH-π stacking is minimized between the WW domain and the docked glycan moiety. Most docking algorithms omit CH-π from their scoring functions, with Autodock Vina and Vina-Carb both showing difficulties being able to accurately predict binding modes when CH-π stacking is involved in a CH/Protein interaction. However, since the model is in its apo form, the CH- π would be minimized, as it is shown in an experimentally derived structure (PDB ID:5a56) (Supplementary Fig. 11) when it’s in its apo form. The position of the anomeric carbon relative to the DDE catalytic triad of the docked glycan moieties is consistent with experimentally determined structures^35^ and does not significantly differ based on average distance of the DDE residues from the anomeric carbon (T-test) (Supplementary Fig. 11 and Supplementary Table 5).

Within the computationally predicted docking poses of Core 1 and Core 3 glycans, we observe some torsion angles that are suboptimal for free glycans. To confirm and optimize the poses of the docked glycans, an algorithm, Vina-Carb was used to address the energetic constraints on intramolecular bonds within the glycan structure. Considering the intramolecular forces did not change the docked position of Core 1 and Core 3 glycans, suggesting that intramolecular constraints from suboptimal torsion angles are compensated for by induced fit and intermolecular contacts with the enzyme active site. Only after applying a penalty of 4.5 kcal/mol for the intramolecular energy of the glycan conformation using Vina-Carb could the α2,3sialylCore 1 glycan moiety be accurately oriented within the active site^35^ (Supplementary Table 5). Taken together, these suggest a complex interplay between the POGase active site geometry and the conformational flexibility of its glycan substrates. When compared to the kinetics of POGase AS, Core 3 appears to have a similar affinity computationally, to Core 1, however, the position of the GlcNAc varies from the position of the Gal in the experimental structure, suggesting even more strain on the Core 3 glycan while being oriented between motif 1 and 2. The computational data for Core 1 and the α2,3sialylCore 1 glycan seem to match well with the kinetics as even more strain is evident on the α2,3sialylCore 1 glycan, leading to a lower affinity experimentally and computationally.

A third motif, YSAWAWV/IEI, exists outside of the GH101 domain that is highly conserved within the POGases, however, its function remains elusive. It is proximal to the GH101 domain on the C-terminal end, possibly having implications in glycan binding and coordination before cleavage. POGases often have more than two glycan-binding domains in addition to the GH101 domain. The determinants of activity of POGases will be further investigated and confirmed by analyzing their crystal or cryo-EM structures in complex with sialylCore 1 O-glycan and other O-glycan substrates, to determine substrate/enzyme interactions and differences more accurately between the POGases and other known O-glycanases.

Identification of the POGases will promote functional O-glycomic research and the development of therapeutic O-glycoproteins. Given the complexity and heterogeneity of O-glycosylation, structural analysis of O-glycans is still challenging as there is no universal O-glycanase that can release all sialylated O-glycans from glycoproteins. The current O-glycan release protocol relies on a chemical method, termed β-elimination, which has several limitations. Five identified and partially characterized POGases have residual activity to cleave α2,6sialylCore 1, and di-sialylCore 1 O-glycans, which also commonly occur on glycoproteins. The characterization of POGase AS showed that it is not a perfect tool yet, especially in application to cellular O-glycomic analysis, most likely due to the limited accessibility to mucin-domains of glycoproteins from cell extracts which is the common obstacle for O-glycomics. More efficient enzymes need to be screened or generated through mutagenesis. Identification of the POGases is a step forward to generate a universal enzyme with the capability to release all sialylated O-glycans. This could be potentially achievable by random mutagenesis or directed evolution approaches assisted by predictive artificial intelligence (AI). Information on POGase structure will greatly facilitate the generation of previously uncharacterized POGases with increased activity and specificity towards sialylated substrates. Interestingly, like many exo-glycosidases and endoglycosidases that were shown to have transglycosylation activity^42–44^, several known O-glycanases also appear to have transglycosylase activity and can transfer the Core 1 disaccharide to aglycone^23,45,46^. The potential transglycosylase activity of POGases needs to be explored and could potentially revolutionize the synthesis of O-glycoproteins/peptides with sialylated O-glycans in vitro. A universal POGase can also make it possible for the characterization and control of O-glycosylation of therapeutic O-glycoproteins, thus facilitating the drug development.

In addition to their glycan hydrolyzing activity, POGases also possess glycan-binding activity that may serve as adhesins for bacteria to colonize and/or invade through attachment to host cells. One of the POGase orthologs tested, POGase TB showed strong and specific binding to O-glycans, the type-I chain in N-glycans, and glycolipids on the Legacy CFGv6 glycan MicroArray (Fig. 8), a powerful platform to identify glycan-binding proteins and their specificities^47^. Furthermore, POGase TB can bind to HEK293 cells, confirming its cell-binding, thus potential adhesin activity. The POGases identified thus far are all expressed in different bacterial species. It is well-known that host cell glycans serve as ligands of bacterial lectins and toxins^48^. Besides digesting these glycans as the energy/nutrition source for the bacteria’s own benefit, the endoglycosidases may also serve as adhesion molecules, or adhesins, and play important roles in their colonization. More importantly, some of those endoglycosidases may assist in invading through the barriers of epithelia and contribute to pathogenesis. For example, EngCP of *Clostridium perfringens* is a virulence factor involved in gangrene infections^49^. The POGase orthologs identified here exist in hundreds of different bacterium species, primarily in *Actinomyces* and *Trueperella* genus. The roles of those POGases in both the microbiology and their potential pathogenesis, including both in humans and animals, are unknown and warrant further investigation. For example, POGases exist in *Actinomyces* and other species of gut microbiota, which are found in association with Crohn’s disease^50–53^, one of the major forms of inflammatory bowel disease. The adhesin function of POGases may play a critical role in the attachment of pathogens to host cells, an initial step for invasion and/or infection. This is supported by the POGase’s high binding to Type-I chains on N-glycans in addition to sialylated O-glycans, which are commonly expressed in human epithelial cells such as GI tract epithelia. A better understanding of its role in microbiology and human diseases may evolve potential therapeutics such as inhibitors and even POGase-based vaccines. One may argue the two proposed activities of POGases, hydrolase and adhesin, are paradoxical. We propose that these two independent activities of POGases are dynamically active at different stages of the life cycle of bacteria as the two activities have two different targets: hydrolase activity that is specific to sialylated O-glycans and adhesin activity for all major types of glycosylation: O-glycans, N-glycans, and glycolipids. The adhesin activity of POGases may possibly be initiated upon contact of glycoproteins, mucins, or even glycolipids on the host cell surface, facilitating the attachment of bacteria for either colonization or invasion, while its glycosidase activity is executed when the glycoproteins on host cells are partially degraded by the concerted action of sialidases, O-glycopeptidases^54^ and peptidases, such as StcE mucinase from *E. coli*^55^. The POGases were observed to cleave the sialylated O-glycans less efficiently from intact and unmodified glycoproteins compared to their denatured counterparts and glycopeptides on short peptide backbones (Fig. 6). In both processes, the C-terminal portion of the POGases appears to be essential, for not only adhesin function but also sufficient hydrolase activity.

The POGases are found within a monophyletic clade suggesting a shared evolutionary path. This clade was found within a larger monophyletic parental clade corresponding to Endo-α-N-Acetylgalactosaminidases derived from the phylum *Actinomycetota* (Fig. 10). Another distinct clade of Endo-α-N-Acetylgalactosaminidases was found to be expressed only within *Firmicutes*. The overall monophyletic nature of these clades suggests a divergent evolutionary event. Since the POGases are only present in *Actinomycetota*, this suggests that these proteins independently evolved in these bacteria as opposed to *Firmicutes*. The evolutionary history of the species expressing POGases seems to match with the evolution of the POGases they express.

It is worth noting that POGase Motif-1 blast hits one ortholog, GenBank: CRH87837.1 in *Chlamydia trachomatis* (termed “POGase CT”), whose sequences were submitted by Wellcome Trust Sanger Institute, CB10 1SA, United Kingdom on 10-MAR-2015. “POGase CT” also contained all 3 motifs of POGase. Sequence alignments of the “POGase CT” with POGase AS showed significant homology (identity: 38.71%, and similarity: 51.86%). We even expressed recombinant “POGase CT” in *E. coli* and purified the protein and demonstrated its expected activity towards a series of sialylated O-glycan substrates. Given the fact that *Chlamydia trachomatis* is well-known to cause sexually transmitted diseases in humans, we contacted Dr. David Ted Hackstadt, an expert on *Chlamydia trachomatis* infection at NIAID, and learned that the GenBank: CRH87837.1 was absent from the *Chlamydia trachomatis* genome, which is nearly completed (99%). Inquiries about the sequence information got no response. It is speculated that the sequence was derived from contamination of other bacterium species when the samples of *Chlamydia trachomatis* were taken from the patient. Surprisingly, blast results of “POGase CT” showed no orthologous with significant homology (>80%), suggesting the GenBank: CRH87837.1 may exist in an unidentified bacterium.

In summary, we have identified five previously unidentified O-glycanases, termed POGases, from different bacteria. These POGases have broad substrate specificities with abilities to cleave sialylCore 1 O-glycans, sialylCore 2, and Core 3 O-glycans, in addition to Core 1 O-glycans, with a higher efficiency compared to known O-glycanases. POGases were also found to specifically recognize and bind to O-glycans, Type-I chains on N-glycans, and even glycolipids that commonly decorate human epithelia, independent of their enzymatic activity. POGases evolutionarily exist in a distinctive clade of bacteria within the genus of bacteria expressing O-glycanases. The identification of POGases could facilitate developing tools for analyzing O-glycans of therapeutic proteins in quality assessment and for functional O-glycomics. The features of POGases also shed light on their potentially significant roles in microbiology and in pathogenesis in human diseases, evolving as potential targets of specific therapeutics for the relevant diseases.

## Methods

### Materials and reagents

Recombinant soluble glycosyltransferases were purchased from Glyco Expression Technologies, Inc. (Athens, GA), including Drosophila T-synthase (Dm-C1GALT1) with GFP tag; human recombinant ST3GAL1; human Core 2 GnT (GCNT2, Core 2 synthase) with GFP tag; human recombinant β4GALT1; and human β3GnT6 (C3GnT, Core 3 Synthase) with GFP tag; and human ST3Gal4 with GFP tag. 4-Methylumbelliferyl 2-acetamido-2-deoxy-α-D-galactopyranoside [GalNAcα-(4-MU)] (Cat# EM04782) and CMP-Neu5Ac (sialic acid) (Cat# MC04391) were purchased from Biosynth Carbosynth (Berkshire, UK). UDP-α-D-Galactose (UDP-Gal, Cat# 670111) and UDP-α-D-N-Acetylglucosamine (UDP-GlcNAc, Cat# 670107) were purchased from Millipore Sigma (Burlington, MA). The following glycopeptides were purchased from Sussex Research (Toronto, Canada): CD24(41-51)-TF(41, 51) (CD24-T_2_, Cat# GP900110); CD24(41-51)-STF(41,51) (CD24-sT_2_, Cat# GP000070); MUC1(138‐157)‐tri-Tn(144,150,151) (MUC1-Tn_3_, Cat# GP100085). MUC5AC-3/13 carrying Tn antigens at Thr3 and Thr13 (MUC5AC-Tn_2_, Cat# AS-61332) was purchased from AnaSpec (Fremont, CA) (Supplementary Table 3). O-glycosidase (Cat# P0733S) was purchased from NEBiolabs (New England, MA). All other chemicals were from either from Sigma-Aldrich (St. Louis, MO) or Fisher Scientific (Hampton, NH), otherwise indicated.

### Synthesis of *Core 1-(4-MU)*, *Core 2-(4-MU)*, *Core 3-(4-MU)*, and *α2,3sialylCore 1-(4-MU)*

#### Synthesis of *Core 1-(4-MU)*

The reaction system for synthesis of *Core 1-(4-MU)* contained 3 µmol of GalNAc-α-(4-MU), 6 µmol of UDP-Gal, and 300 µg of recombinant Dm-C1GALT1 (T-synthase) in 3 mL of 100 mM MES-NaOH (pH 7.0), 20 mM MnCl_2_, and incubated at 37 °C for 18 h. The reaction was confirmed to be >99% completed by analytical RP-(U)HPLC, ACQUITY UPLC system (Waters) with a BEH C_18_ column (1.7 µm, 2.1 × 50 mm) using a gradient of 0-40% B for 5 min, then 40–95% B for 2 min, and 95% B for additional 0.2 min under 0.3 mL/min flow rate at room temperature (solvent A = 100% H_2_O + 0.1% trifluoroacetic acid (TFA); solvent B = 100% CH_3_CN + 0.1% TFA). The identity of the product was verified using a Bruker UltrafleXtreme MALDI-TOF/TOF MS (Bruker) in positive reflector mode (calculated MW: 541.5 Da, observed MW: 543.4 Da [M + H]^+^; Supplementary Fig. 1b).

#### Synthesis of *α2,3sialylCore 1-(4-MU)*

The reaction system for synthesis of *α2,3sialylCore 1-(4-MU)* contained 1.5 µmol of GalNAc-α-(4-MU), 3 µmol of UDP-Gal, 3 µmol of CMP-SA,150 µg units of recombinant T-synthase, and 150 µg of recombinant ST3GAL1 in 1.5 mL of 100 mM MES-NaOH(pH 7.0), 20 mM MnCl_2_, and incubated at 37 °C for 18 h. The reaction was confirmed to be >99% completed by analytical RP-(U)HPLC using a gradient of 25–65% B over 5 min (solvent B = 100% CH_3_CN + 0.1% TFA). The identity of the product was verified by MALDI-TOF MS (calculated MW: 832.8 Da, observed MW: 855.5 Da [M + Na]^+^; (Supplementary Fig. 1c).

#### Synthesis of *Core 2-(4-MU)*

The reaction system for synthesis of *Core 2-(4-MU)* contained 1000 µL of reaction mixture from above containing 1.5 mM Core 1-(4-MU) with 25 mM EDTA, 3 mM UDP-GlcNAc, and 300 µg of recombinant GCNT2. The reaction was incubated at 37 °C for 18 h. The product was confirmed to be >99% completed by analytical RP-(U) HPLC using a gradient of 10–40% B over 5 min (solvent B = 100% CH_3_CN, 0.1% TFA). The identity of the product was verified by MALDI-TOF MS (calculated MW: 744.7 Da, observed MW: 745.1 Da; Supplementary Fig. 3a).

#### Synthesis of *Core 3-(4-MU)*

The reaction system for synthesis of *Core 3-(4-MU)* contained 2.5 µmol of GalNAc-α-(4-MU), 5 µmol of UDP-GlcNAc, and 300 µg of recombinant β3GnT6 (Core 3 GnT) in 1500 µL of 100 mM MES-NaOH (pH 7.0), 20 mM MnCl_2_, and incubated at 37 °C for 18 h. The product was confirmed to be >99% completed by analytical RP-(U) HPLC using a gradient of 10–40% B over 5 min (solvent B = 100% CH_3_CN + 0.1% TFA). The identity of the product was verified by MALDI-TOF MS (calculated MW: 582.6 Da, observed MW: 583.1 Da; Supplementary Fig. 3b).

### Synthesis of sialylated O-glycopeptides

#### *Core 1 MUC5AC*

The total 250 µL reaction system contained 500 µg of *MUC5AC-Tn*_*2*_, 10 mM UDP-Gal, 20 mM MnCl_2_, and 50 µg of recombinant T-synthase in 100 mM MES-NaOH (pH 7.0). The reaction mixture was incubated at 37 °C for 18 h. One-half of 1 µL of reaction mixture was diluted with 9.5 µL of H_2_O, then 0.5 µL of diluted mixture was spotted on the MALDI-TOF MS target plate (MTP Targets, Cat# 8280784, Bruker), and mixed with 0.5 µL of 2,5-dihydroxybenzoic acid (DHB, 10 mg/mL containing 1 mM NaCl in 30% ACN, 0.1% TFA) matrix. After drying, the sample was analyzed on MALDI-TOF MS (Supplementary Fig. 5).

#### *α2,3sialylCore 1 MUC5AC*

The total 250 µL reaction system contained 200 µg of *MUC5AC-Tn*_*2*_, 10 mM UDP-Gal, 10 mM CMP-SA, 20 mM MnCl_2_, 30 µg of recombinant T-synthase, and 30 µg of recombinant ST3GAL1 in 100 mM MES-NaOH (pH 7.0). The reaction was incubated at 37 °C for 18 h. One-half of 1 µL of reaction mixture was diluted with 9.5 µL of H_2_O, then 0.5 µL of the diluted mixture was spotted on the target plate, mixed with 0.5 µL of DHB, and analyzed by MALDI-TOF MS (Supplementary Fig. 5).

#### *α2,3sialylCore 2 MUC5AC*

Two-step syntheses were carried out. The first step was to the synthesis of *Core 2 MUC5AC*: The 150 µL reaction system contained 200 µg of *MUC5AC-T*_*2*_ (Core 1 MUC5AC) solution from the above reaction with 25 mM EDTA, 3 mM UDP-GlcNAc, and 30 µg of recombinant GCNT2. The reaction was incubated at 37 °C for 18 h. One microliter of diluted reaction mix (1:20 in water) was analyzed on MALDI-TOF to confirm >95% completion (Supplementary Fig. 5). Second step was to synthesize *α2,3sialylCore 2 MUC5AC*: the 250 µL reaction system contained: 200 µg of *Core 2 MUC5AC*, 5 mM UDP-Gal, 5 mM CMP-SA, 20 mM MnCl_2_, 20 µg of recombinant β4GALT1, and 30 µg of ST3Gal1 (pH 7.0). The reaction was incubated at 37 °C for 18 h. One-half of a microliter of the reaction mixture was analyzed by LC-MS (Supplementary Fig. 5).

### Cloning, expression, and screening of active POGases on α2,3sialylCore 1-(4-MU)

Based on EC 3.2.1.97, endo-α-N-acetylgalactosaminidase from BRENDA, a comprehensive enzyme information system, 30 genes encoding the putative O-glycanases (Supplementary Table 1, #1–30) were synthesized and codon-optimized for *E*scherichia *coli* (*E. coli*) expression. A signal sequence, MKKITAAAGLLLLAAQPAMA, was added at their N-terminus and any predicted transmembrane domain was eliminated from the ORF. The modified expression sequences were cloned into the expression vectors pET301/CT-DEST (Arizona State University, Biodesign Institute, DNASU Plasmid Repository) with a 6xHis tag at their C-terminus. An additional 16 putative O-glycanases from different bacteria were also selected from top hits with >31% overall sequence identity to POGase TB (#22) (Supplementary Table 1, #31–46). Their ORFs were optimized, synthesized, and cloned into the pET-29b (+) expression vectors (Genscript, New Jersey) with a 6xHis tag at their C-terminus. For each POGase clone, BL21 (DE3) competent *E. Coli* cells (Cat# C600003, Thermo Fisher Scientific) were transformed with the respective constructs, and grown in 1 mL LB media (Cat: 3002036, MP Biomedicals, Solon, OH) at 37 °C until reaching a mid-log phase. Overexpression of the POGase protein was then induced by the addition of 0.5 mM Isopropyl β-D-1-thiogalactopyranoside (IPTG) (Cat# I6758, Sigma, St. Louis, MO) at 22 °C for 18 h. After collecting the cells by centrifugation at 6000 × *g* for 15 min, the cell pellet was resuspended and lysed in 200 µL of B-PER™ Bacterial Protein Extraction Reagent (Cat# 78243, Thermo). The insoluble material was removed by centrifugation at 17,000 × *g* for 10 min. The supernatant was diluted at 1:20 in TBS (20 mM Tris-HCl, 150 mM NaCl, pH 7.4). One microliter of the diluted supernatant was combined with α2,3sialylCore 1-(4-MU) (0.5 nmol) or Core 1-(4-MU) (0.5 nmol) in 9 µL of TBS and incubated at 37 °C. Images were taken under UV (301 nm wavelength) after 30 min, 1 h, 2 h, 3 h, or overnight. For quantitative comparison, 1 µL of undiluted supernatant was combined with α2,3sialylCore 1-(4-MU) (0.5 nmol) or Core 1-(4-MU) (0.5 nmol) in 99 µL of TBS in 96-well black plates and incubated at 37 °C for 30 min. One hundred microliters of 1 M glycine-NaOH (pH 10.0) was added to each well and the relative fluorescence units (RFU) were measured on a plate reader (SpectraMax® i3, Molecular Devices, San Jose, CA) using a released umbelliferone detection (Ex., 355 nm; Em., 460 nm).

### Purification of POGases

The supernatant was incubated overnight at 4 °C with Ni-NTA beads (Thermo Fisher, Cat# 88221) pre-equilibrated with TBS. The beads were washed with 10-column volumes of wash buffer (TBS with 25 mM imidazole, pH 7.4), and eluted with 5-column volumes of wash buffer containing 300 mM imidazole. Pure fractions were pooled and analyzed by SDS-PAGE and Western blot.

### Kinetic characterization

Michaelis–Menten kinetic parameters were determined for the substrate Core 1-(4-MU), Core 2-(4-MU), Core 3-(4-MU), or α2,3sialylCore 1-(4-MU) for POGase AS and O-glycosidase (EngEF, P0733S, New England Biolabs, MA). Kinetic assays were carried out at 37 °C in 20 mM citrate/HCl, 50 mM NaCl, pH 6.0 (total volume of 50 µL) in 96-well black plates for fluorescence assays. The increased fluorescence resulting from MU release was measured using a plate reader. Reactions were performed with varying concentrations of substrates and enzymes. Initial rates (µM/min) were converted from absorbance to concentration using MU standard concentration curves determined under identical reaction conditions. Kinetic parameters were then determined by fitting to the Michaelis–Menten equation using a nonlinear regression method in GraphPad Prism 8.

### Release and analysis of O-glycans from glycopeptides

One µg of glycopeptides and 0.5 µg of POGase enzymes were combined in 10 µL of reaction buffer (20 mM citrate/HCl, 50 mM NaCl, pH 6.0) and incubated at 37 °C for 16 h. The mixture of released glycans was subsequently analyzed on a nano C_18_-LC-MS/MS using a gradient of 2–50% B in H_2_O containing 0.1% formic acid (FA) over 45 min (solvent B = 100% CH_3_CN, 0.1% FA).

To optimize O-glycan release with POGase, the undenatured glycoproteins were digested with POGase AS compared to glycoproteins which were reduced, alkylated and denatured as described in the Filter Aided N-glycan Separation (FANGs) protocol (except POGase AS in NEBiolab’s Glycobuffer 2, which is 50 mM sodium phosphate (pH 7.5), was used for digestion)^14,56^. For analysis of POGase-released O-glycans from synthetic glycopeptides, 10 µg of glycopeptide was combined with 5 µg of enzyme in 100 µL of reaction buffer (Glycobuffer 2) and incubated at 37 °C for 16–24 h. The reaction mixture was passed through a 30 kDa molecular weight cutoff filter (Filter Aided Sample Prep (FASP) protein digestion kit, Cat# ab270519, Abcam) to remove the enzyme, and the filtrate was acidified by adding 100 µL of 0.2% TFA. Peptides were removed by passing the filtrate through a 50 mg HyperSep^TM^ C18 cartridge (Cat# 60108-301, Thermo, Waltham, MA). The retained peptides were washed three times with 5% acetonitrile (ACN), and 0.1% TFA and eluted with 900 µL of 85% ACN and 0.1% TFA for analysis by LC-MS/MS using a Waters Xevo G2 coupled to a Waters nano C_18_-LC-MS/MS.

O-glycans recovered in the C_18_ flowthrough were purified by HyperSep^TM^ NH2 cartridges (Cat# 60108-364, Thermo) used in a HILIC mode. The concentration of ACN was adjusted to 80% containing 0.1% TFA and loaded on the cartridges and passed through a total of three times. The resin was washed with 3 mL of 90% ACN and 0.1% TFA and finally eluted in a total of 900 µL of 10% ACN and 0.1% TFA. The O-glycans were dried by vacuum centrifugation and reduced with 75 µL of a 10 mg/mL ammonia borane solution and incubated at 60 °C for 1 h. Excess reactants were removed by a series of methanolic evaporations in which 200 µL of 10% acetic acid in 90% methanol was added to the reduced O-glycans and evaporated by vacuum centrifugation. This process was repeated a total of five times. Reduced O-glycans were permethylated by solid-phase permethylation followed by C_18_ purification as described^14,31^. O-glycans were dried by vacuum centrifugation.

### Release and analysis of O-glycans from glycoprotein drugs by filter-aided approach

O-glycans from glycoprotein drugs etanercept and abatacept and bovine fetuin were released in a manner consistent with FANGs methodology as mentioned above, with POGase used instead of PNGase F^14,56^. Essentially, 50 µg of proteins were reduced and alkylated as described with materials provided in the FASP protein digestion kit. After buffer exchange into 50 mM sodium phosphate, pH 7.5 (Glycobuffer 2), 25 µg of POGase AS was added onto the filter and the samples were incubated for 24 h at 37 °C. Released O-glycans were recovered by centrifugation at 15,000 × *g* for 15 min, and the filter was washed with 100 µL of 0.2% TFA in water. The O-glycan mixture was diluted with 800 µL of ACN, 0.1% TFA, and purified by HyperSep^TM^ NH2 cartridges followed by reduction and solid-phase permethylation as described in the previous section.

### Release and analysis of O-glycans from glycopeptides of protein drugs

Fifty µg of fetuin, etanercept, and abatacept were processed with materials from a FASP protein digestion kit as described above and the glycoproteins were digested on the filter with 20 µg of proteinase K (Cat# 70-663-4, Millipore Sigma) in 100 mM Tris/HCl, pH 8.5, at 50 °C for 24 h. The short glycopeptides were recovered by centrifugation at 15,000 × *g* for 15 min and the filter was washed with 100 µL of water. Twenty microliters of 10x Glycobuffer 2 were added to the reaction mixture, and 25 µg of POGase AS was added. The samples were then incubated for 24 h at 37 °C. Excess POGase enzyme was removed by passing the reaction mixture through a 30 kDa cutoff FASP filter, and free O-glycans were separated from O-glycopeptides by C_18_ purification. O-glycans recovered in the flowthrough were then purified further by NH2 cartridges, reduced, and permethylated as described in the previous sections.

### MALDI-TOF/MS analysis of permethylated O-glycans

Dry O-glycans were resuspended in 50% methanol, mixed 1:1 with DHB, and spotted on a target plate for analysis by a Bruker UltrafleXtreme MALDI-TOF/TOF MS in a positive reflector mode. Permethylated O-glycans were detected as sodiated and reduced ions. O-glycan mass calculations and tandem mass spectra predictions were performed using Glycoworkbench 2.1 (https://code.google.com/archive/p/glycoworkbench/).

### Structural modeling and substrate docking of POGases

The FASTA sequence of the POGase AS was folded into 3D models using various modeling algorithms including AlphaFold2^54–57^, SWISS_MODEL^57^, and Phyre2^58^. Each model was individually compared across various quality metrics including QmeanDisCO global, Qmean Z, Cβ, All Atom IDDT, solvation, and torsion scores^59,60^. The highest quality model by all metrics was then used to conduct protein-ligand docking to model the active site of the POGases. Initial Ligand PDB files were generated and downloaded from the Glycam Carbohydrate builder (http://glycam.org). To prepare ligands for docking, hydrogens were added using OpenBabel^61^, oligosaccharides were manually kekulized, and active torsion and a generalized amber force field were applied for the flexibility of the glycan. To confirm accurate carbohydrate models, all structures were compared to known 2D models to ensure the correct placement of functional groups. Similarly, to prepare the receptor (GH101 Domain of POGase AS) for docking, hydrogens were added, aromatic residues were manually kekulized, and the receptor was set to rigid. The resulting PDBQT files were then used to conduct the docking. Docking was run with AutoDock Vina^62^, and Vina-Carb^63^ with each specified O-glycan (Core 1, Core 3, and α2,3sialylCore 1) being separately docked at 20 poses, with exhaustiveness of 40 and energy range of 3 for Vina and Vina-Carb. Glycans were docked into the active site cleft using the coordinates 2.11, −7.94, and −1.18 for the center *x*, *y*, and *z* coordinates for AutoDock Vina to localize the docked poses into the active site cleft previously identified for GH101 domains^35^. For Vina-Carb, the same coordinates, number of poses, and energy range were used but also had a Chi energy coefficient of 1 and Chi energy cutoff of 2 to better incorporate carbohydrate-specific intramolecular forces. For comparison to experimentally solved GH101 domains, the POGase AS/glycan complex was aligned to PDB ID:5a56 (https://www.rcsb.org/structure/5a56) which is a crystal structure of a GH101 domain with a Core 1 agonist in the active site cleft. To ensure a more accurate model, the pose that best reflected the experimental structure was chosen after three computational replicates. A standard student *T*-test was used to compare the distances of the DDE catalytic triad to the anomeric carbon. No molecular dynamics simulations were conducted for this study.

### Expression and purification of N-terminal domain and C-terminal domain of POGase TB

The genes encoding the peptide corresponding to the N-terminal domain, 193–925aa, and C-terminal domain, 876–1839aa of POGase TB with a 6xHis tag at their C-terminus were codon-optimized, synthesized, and cloned into the pET-30a (+) expression vectors (Genscript, New Jersey). The signal peptide, MKKITAAAGLLLLAAQPAMA, was added to the N-terminus of each construct. The plasmids were transformed into *E. coli* cells, BL21 (DE3), and the recombinant proteins were expressed and purified using the same strategies and procedures for other POGases as described above.

### GlycanArray analysis

The glycan-binding activity of POGases was tested on Glycan MicroArray (Legacy CFGv6)^47^ by the Emory Glycomics and Molecular Interactions Core (EGMIC) (https://www.cores.emory.edu/egmic/index.html) using the standard protocol with some modifications, which is mainly at 4 °C instead of room temperature. Briefly, POGases were diluted to 100 µg/mL in Tris buffer (20 mM Tris, 150 mM NaCl, 2 mM CaCl_2_, 2 mM MgCl_2_, pH 7.4) with 1% BSA and 0.05% Tween 20 to a final total volume of 100 µL. The diluted sample was applied on the slide (Legacy CFGv6) and incubated at 4 °C in a dark humidified chamber for 2 h. The slide was washed four times with Tris buffer containing 0.05% Tween 20 and four times with Tris buffer. Then, 100 µL of 5 µg/mL of Alexa Fluor® 647-labeled anti-His Tag antibody (Cat# 652513, BioLegend) was added to the slide and incubated at 4 °C in a dark humidified chamber for 1 h. The slide was washed four times with Tris buffer containing 0.05% Tween 20, four times with Tris buffer, and then four times with water. After drying up by spinning, the slide was scanned with InnoScan 1100AL scanner (Innopsis, France) (resolution: 5 µm/pixel, Alexa 647: PMT 85/Laser Power High) with data acquisition performed by a Mapix 9.1.0 software (Innopsys, Chicago, IL). Data were analyzed using an Excel macro developed by the EGMIC^64^.

### Cell-binding assay

HEK293 cells (Cat# CRL-1573, ATCC) with *Cosmc* knockout (HEK293-CKO) were established with a procedure described previously^14^. Approximately 1 × 10^5^ cells of HEK293-WT and -CKO lines were seeded in 4-well chamber slides (Cat# 154526, Thermo Fisher Scientific), and cultured at 37 °C incubator and 5% CO_2_. The next day, cells were washed with PBS and treated with 50 mU pan-neuraminidase (Cat# P0720L, New England Biolabs) in PBS for 2 h at 37 °C, then fixed with fixation buffer (Cat# 420801, BioLegend) for 10 min at RT. After blocking with 5% (w/v) BSA (Cat# A30075, Fraction V, Research Product International) in PBS for 1 h at RT, cells were incubated with 50 μg/mL of full-length (FL), N-, or C-terminus domains (ND, or CD) of POGase TB (#22) in cold PBS overnight at 4 °C. Cells were washed 3× with cold PBS, then incubated with Alexa Fluor^TM^ 488-labeled mouse anti-6x-His Tag mAb (HIS.H8, Cat# MA1-21315-A488, Thermo Fisher Scientific, diluted at 1:200 in cold PBS) for 1 h at 4 °C. After washing 3× with cold PBS, then slide was covered with mounting medium with DAPI (Cat# H-1200, Vectashield, Vector Laboratories, Inc.), and analyzed on a fluorescent microscope (EVOS fl, AMG, 20× magnification), and images were collected. In parallel, fixed cells were incubated with biotinylated PNA (Peanut Agglutinin, Cat# B-1075-5, Vector Laboratories, diluted to 1 μg/mL in PBS), or anti-Tn mouse IgM (BaGs6 in-house, diluted at 1:500 in PBS), and signals were detected with streptavidin DyLight^TM^ 549 (Cat# SA-5549, Vector Laboratories), or Alexa Fluor^TM^ 568-labeled goat anti-mouse IgM (Cat# A21043, Invitrogen) at 1:400 dilution in PBS.

### Evolutionary analysis and multiple sequence alignments

Sequences of the POGases were aligned to sequences of carbohydrate-binding proteins, galactosaminidase family proteins, and CAZyme family proteins with lengths of 1000 aa–4000 aa. All sequences were derived from the NCBI database. This tree was used as a database tree, from which the GH101 family of enzymes was pruned and analyzed. Another tree was generated using the 16s rRNA sequences of *Actinomycetota* and *Firmicutes*, using sequences derived from the NCBI database. Before processing the multisequence FASTA generated from these sequences, the CD-hit algorithm^65^ was used to eliminate redundant sequences, with the similarity threshold being set to 90% for the carbohydrate-binding protein tree and 95% for the 16s tree.

For the highly diverged carbohydrate-binding protein tree, MAFFT^66^ was used for the multiple sequence alignment. Because the 16s rRNA sequences are housekeeping genes and are more conserved amongst the selected taxa, Clustal Omega^67^ was used for the multiple sequence alignment. Alignments were trimmed using TrimAl’s gappyout algorithm^68^. With the sequences aligned and the alignments processed, maximum likelihood trees were generated using IQ-TREE^69^, with the substitution model being allowed to be chosen by the algorithm based on the data. For the 16s tree, the best model was calculated to be SYM + I + G4 substitution model, while for our functional gene tree, WAG + F + I + G4 was the substitution model chosen based on the sequences in the dataset (chosen by IQtree). After the generation of the maximum likelihood trees, the trees were annotated and visualized using iTOL^70^. To bridge the gap between structural analysis and evolutionary analysis, evolutionary coupling analysis^71^ was done on POGase AS and PDB ID:5a56 (https://www.rcsb.org/structure/5a56).

The FASTA sequences of the POGases (POGase TB, POGase TP, POGase AS, POGase KS, and POGase BN) were aligned to one another, as well as the sequences of various known O-glycanases (EngCP, EngEF, EngSP, EngBF, and EngPA) using Clustal Omega and then viewed and annotated using Jalview^72^.

### Reporting summary

Further information on research design is available in the Nature Portfolio Reporting Summary linked to this article.

## Supplementary information

Supplementary Information

Description of Additional Supplementary Files

Supplementary Movie 1

Supplementary Movie 2

Reporting Summary

Transparent Peer Review file

## Source data

Source Data 1

Source Data 2

Source Data 3

## Data Availability

All data supporting the findings of this study are available in the article and supplementary files. The mass spectrometry (LC-MS) proteomics data have been deposited to the ProteomeXchange Consortium via the PRIDE with an accession number PXD051707. The structural docking analysis utilized PDB structures 5A56 for all experimental structure comparisons. Source data are provided with this paper.
