# Supplementary material for: Dual functional POGases from bacteria encompassing broader O-glycanase and adhesin activities
Source: Nat Commun. 2025 Feb 25;16:1960. doi: 10.1038/s41467-025-57143-8 (PMC11861894; doi:10.1038/s41467-025-57143-8)
Supplement: Supplementary file 1 — Supplementary Information [file 41467_2025_57143_MOESM1_ESM.pdf]

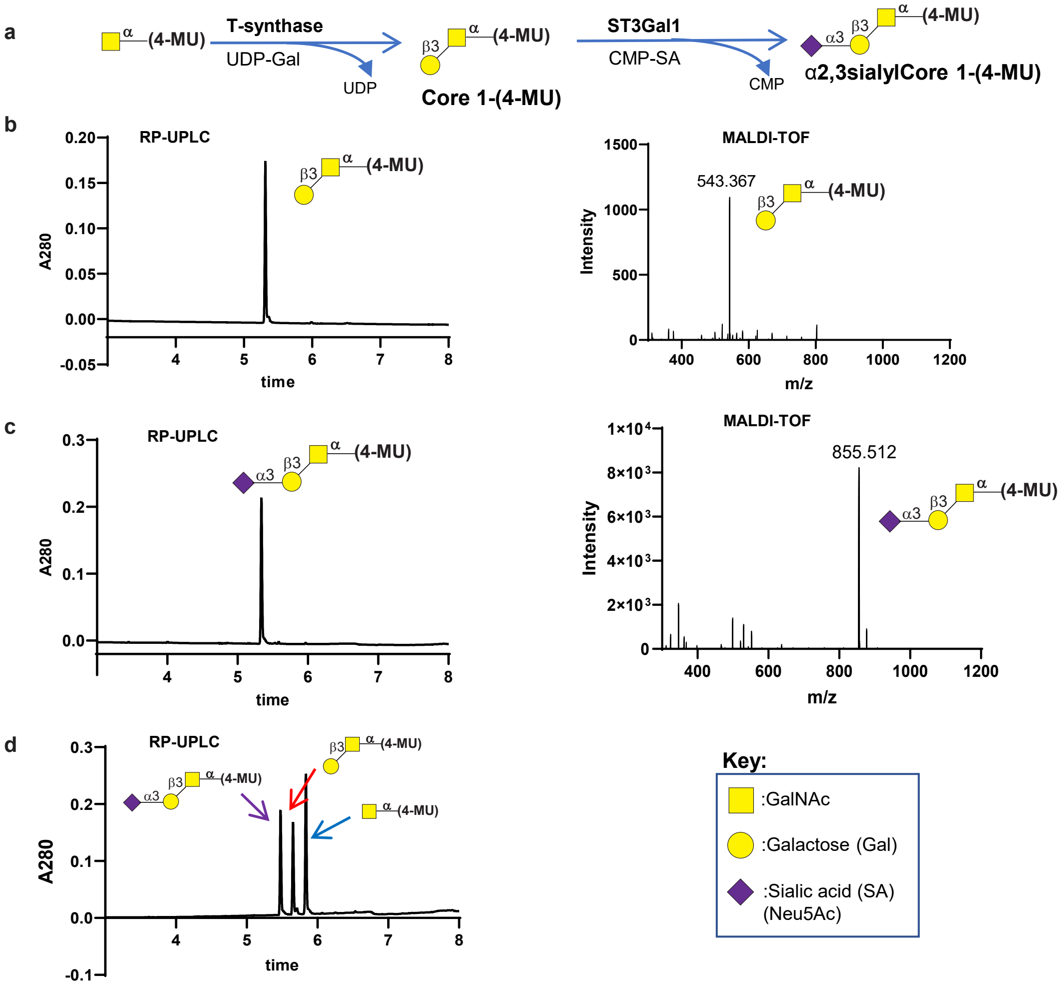

Supplementary Figure 1. Synthesis of Core 1- and α2,3sialylCore 1-(4-MU). a. Schematic strategy of synthesis of fluorogenic substrates. **b**. RP-UPLC and MALDI-TOF-MS analysis of Core 1-(4-MU) (MW: 541.5 Da calculated, 543.367 Da observed). **c**. RP-UPLC and MALDI-TOF-MS analysis of α2,3SialylCore 1-(4-MU) (MW: 832.8 Da calculated, 855.512 Da $(M+H+Na)^{+1}$ observed). **d**. Further RP-UPLC analysis of the mixture of synthesized α2,3sialylCore 1-(4-MU), Core 1-(4-MU), and acceptor GalNAcα-(4-MU).

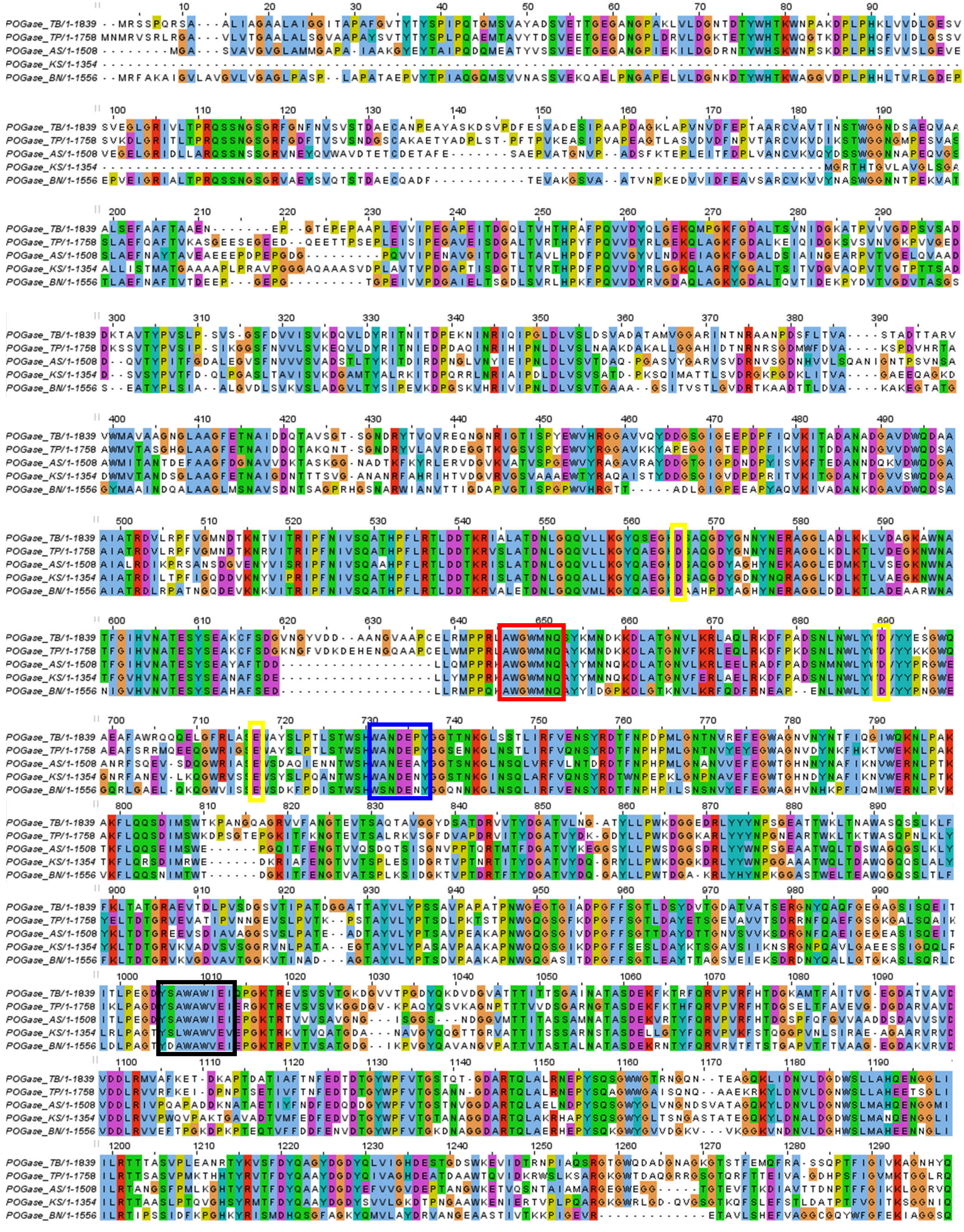

POGase_TB/1-1839
POGase_TP/1-1758
POGase_AS/1-1508
POGase_KS/1-1354
POGase_BN/1-1556

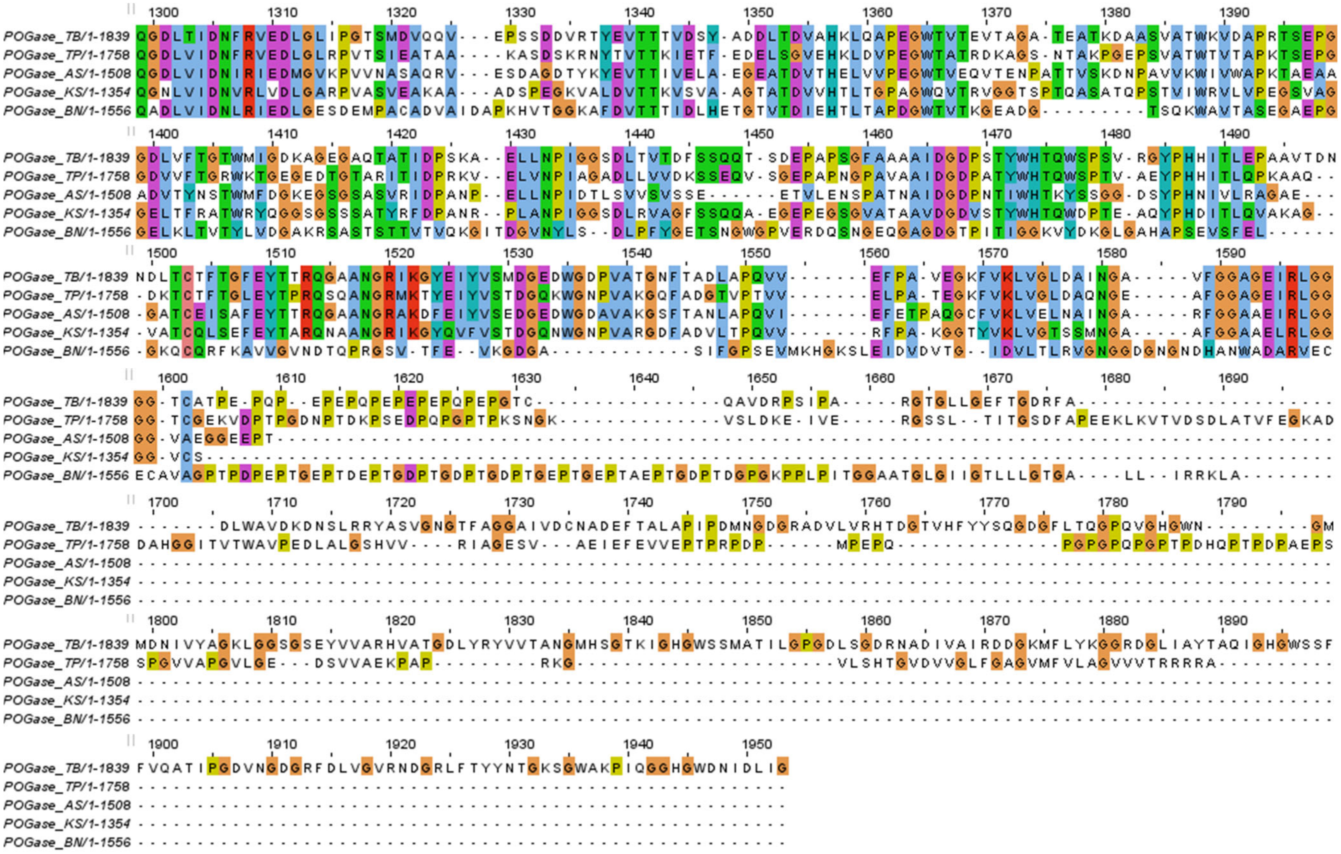

Supplementary Figure 2. Multiple sequence alignment of the 5 POGases identified in this study. Sequences were aligned with Clustal Omega and alignment was viewed using Jalview. For ease of analysis, residues were annotated and colored according to their clustal grouping. Motif 1 (red), motif 2 (blue), and motif 3 (black) along with the DDE catalytic triad residues (yellow boxed) are all indicated.

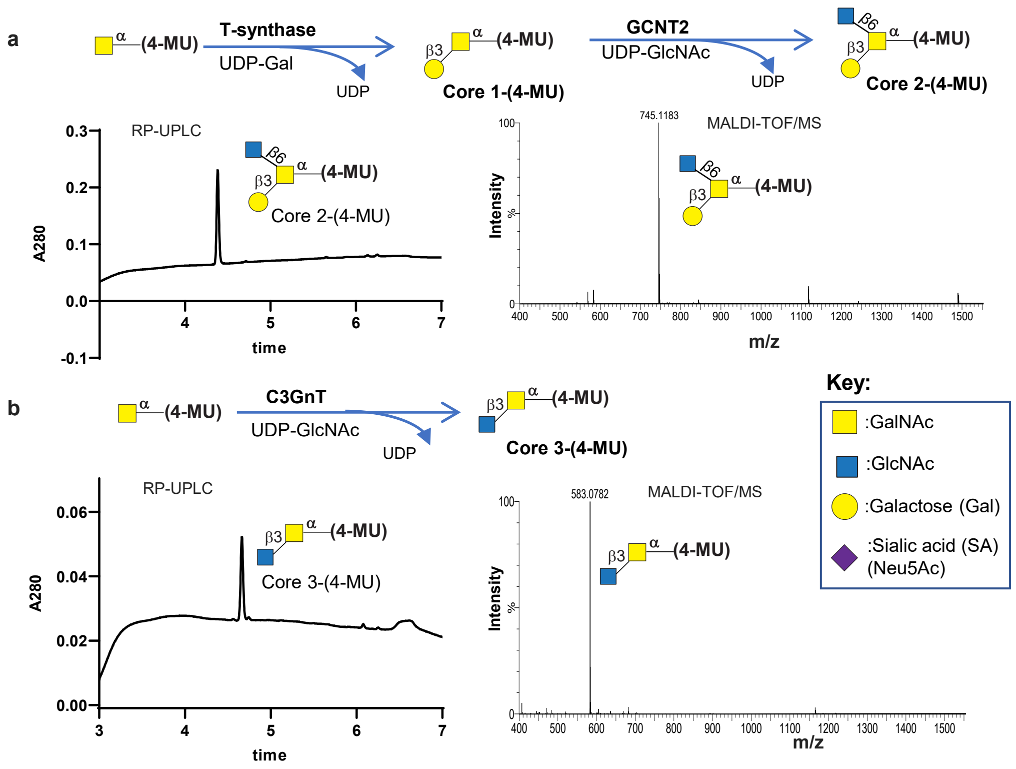

Supplementary Figure 3. Synthesis of Core 2- and Core 3-(4-MU). a. RP-UPLC and MALDI-TOF-MS analysis of Core 2-(4-MU) (MW: 744.7 Da calculated, 745.1 Da observed). **b**. RP-UPLC and MALDI-TOF-MS analysis of Core 3-(4-MU) (MW: 582.6 Da calculated, 583.1 Da observed).

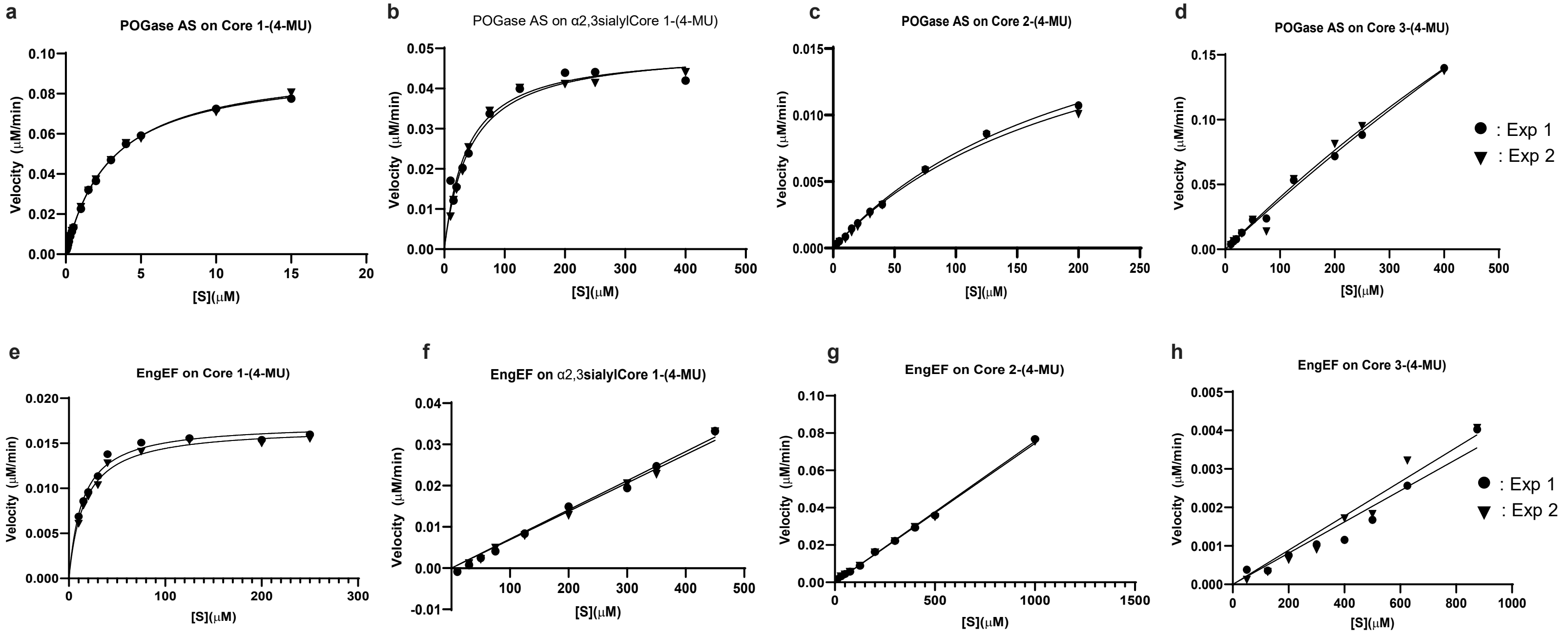

Supplementary Figure 4. Kinetic characterization of POGase AS. **a**. [Et]=61 pM. **b**. [Et]=610 pM. **c**. [Et]=61 pM. **d.** [Et]=610 pM. **e**. [Et]=688 pM. **f.** [Et]=6.88nM. **g**. [Et]=3.44nM. **h**. [Et]=3.44nM. [Et]: The total concentration of enzyme, POGase AS in different kinetic studies. Source data are provided as a Source Data file.

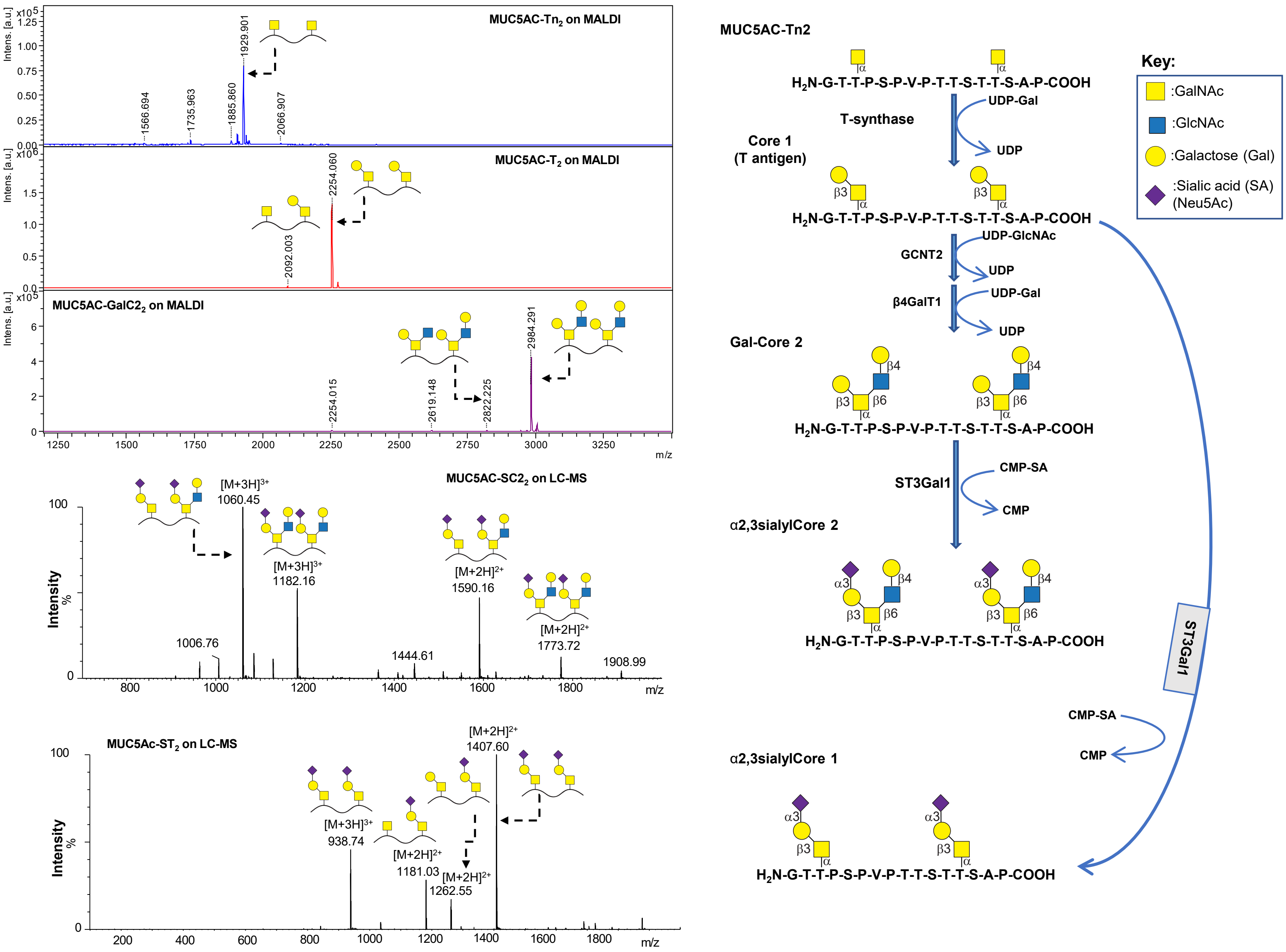

Supplementary Figure 5. Synthesis of α2,3sialylCore 1-MUC5AC, sialylCore 2-MUC5AC. α2,3sialylCore 1-MUC5AC, sialylCore 2-MUC5AC were synthesized with serial reactions by corresponding recombinant glycosyltransferases. The reactions were monitored and confirmed by either MALDI-TOF for neutral glycopeptides, or LC-MS for the sialylated glycopeptides.

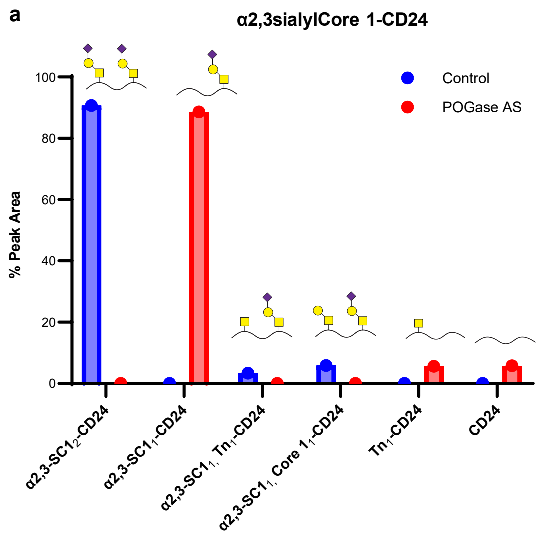

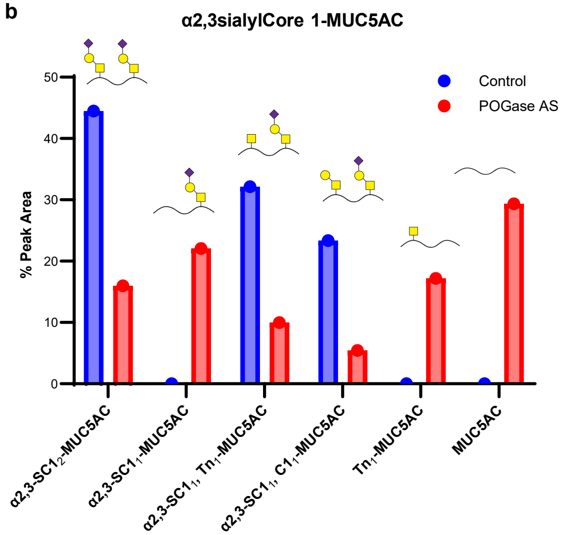

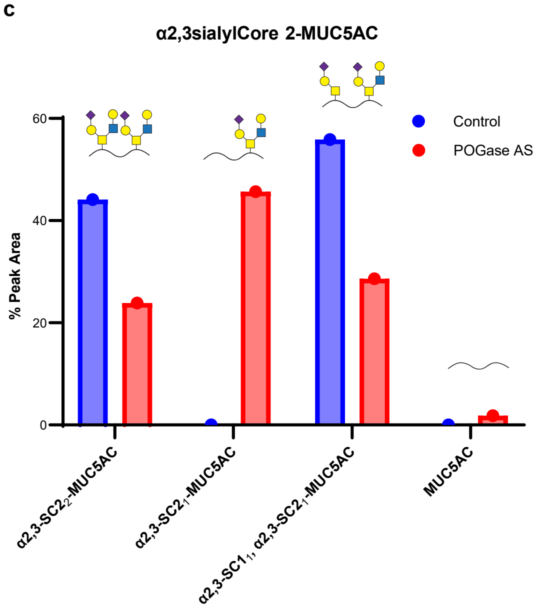

Supplementary Figure 6. Peak area from extracted ion chromatograms (XIC) of glycopeptide products after POGase AS treatment (n=1). a. α2,3sialylCore 1-CD24, **b**. α2,3sialylCore 1-MUC5AC, **c**. α2,3sialylCore 2-MUC5AC. Source data are provided as a Source Data file.

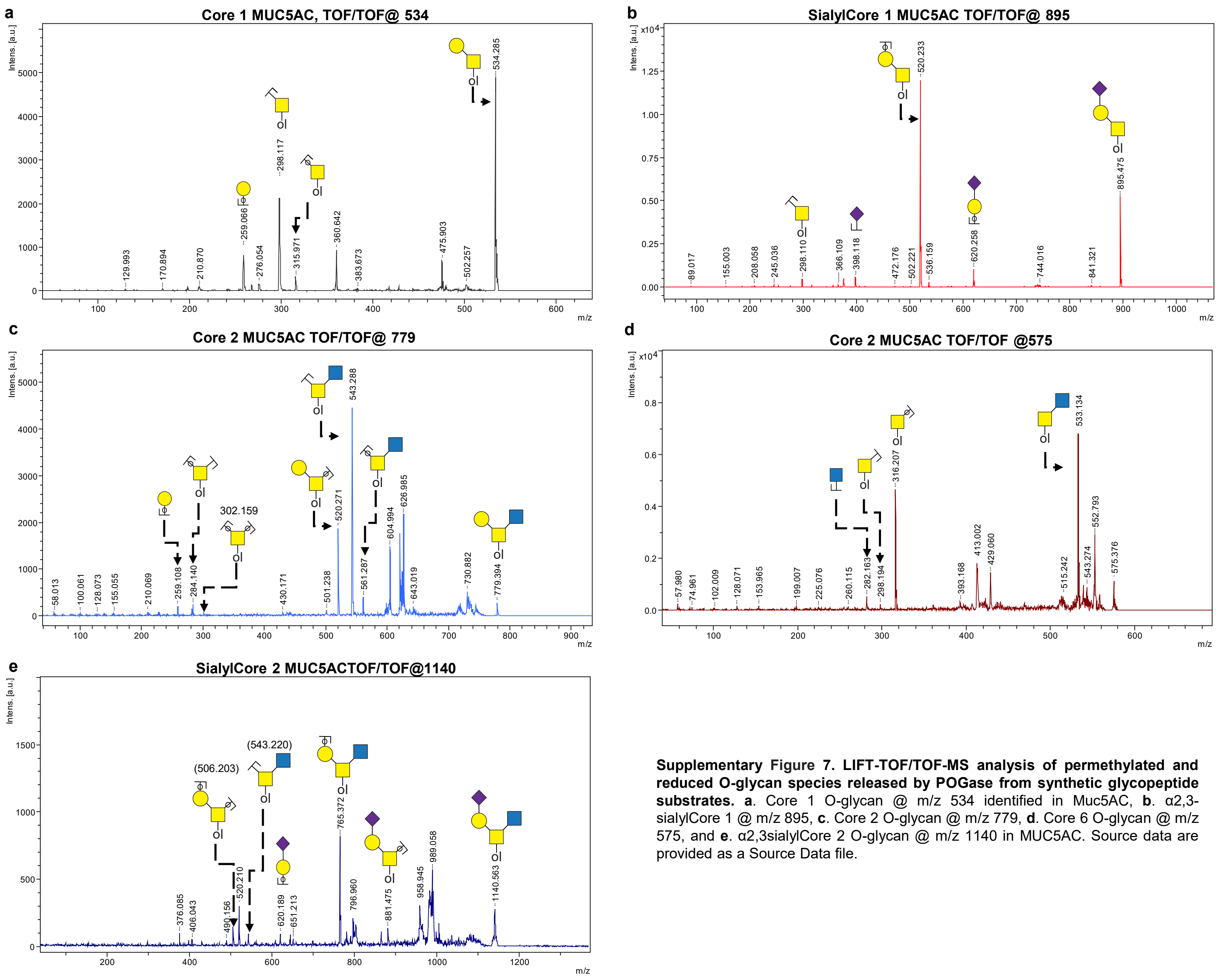

Supplementary Figure 7. LIFT-TOF/TOF-MS analysis of permethylated and reduced O-glycan species released by POGase from synthetic glycopeptide substrates. a. Core 1 O-glycan @ m/z 534 identified in Muc5AC, **b**. α2,3-sialylCore 1 @ m/z 895, **c**. Core 2 O-glycan @ m/z 779, **d**. Core 6 O-glycan @ m/z 575, and **e**. α2,3sialylCore 2 O-glycan @ m/z 1140 in MUC5AC. Source data are provided as a Source Data file.

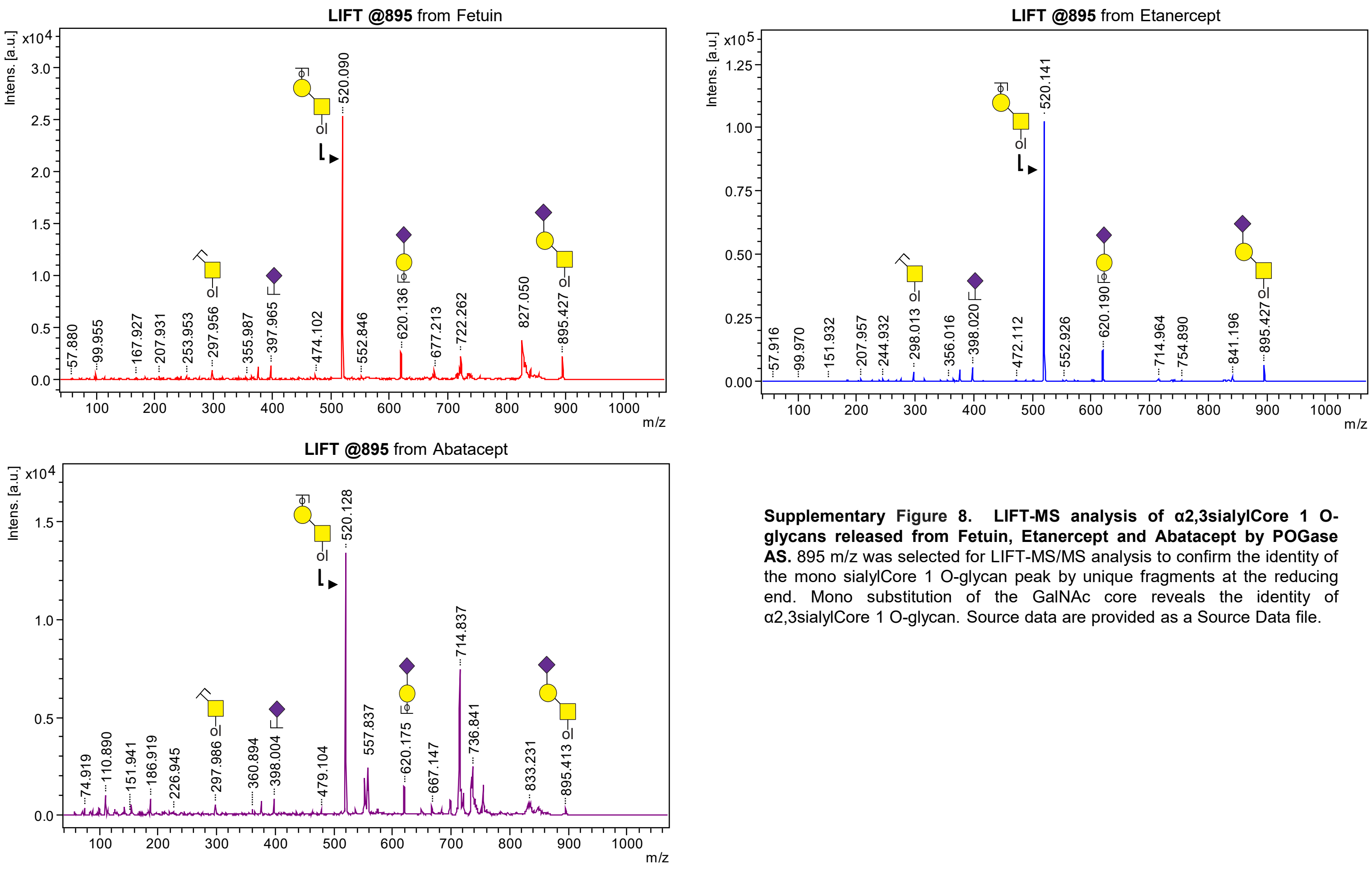

Supplementary Figure 8. LIFT-MS analysis of α2,3sialylCore 1 O-glycans released from Fetuin, Etanercept and Abatacept by POGase AS. 895 m/z was selected for LIFT-MS/MS analysis to confirm the identity of the mono sialylCore 1 O-glycan peak by unique fragments at the reducing end. Mono substitution of the GalNAc core reveals the identity of α2,3sialylCore 1 O-glycan. Source data are provided as a Source Data file.

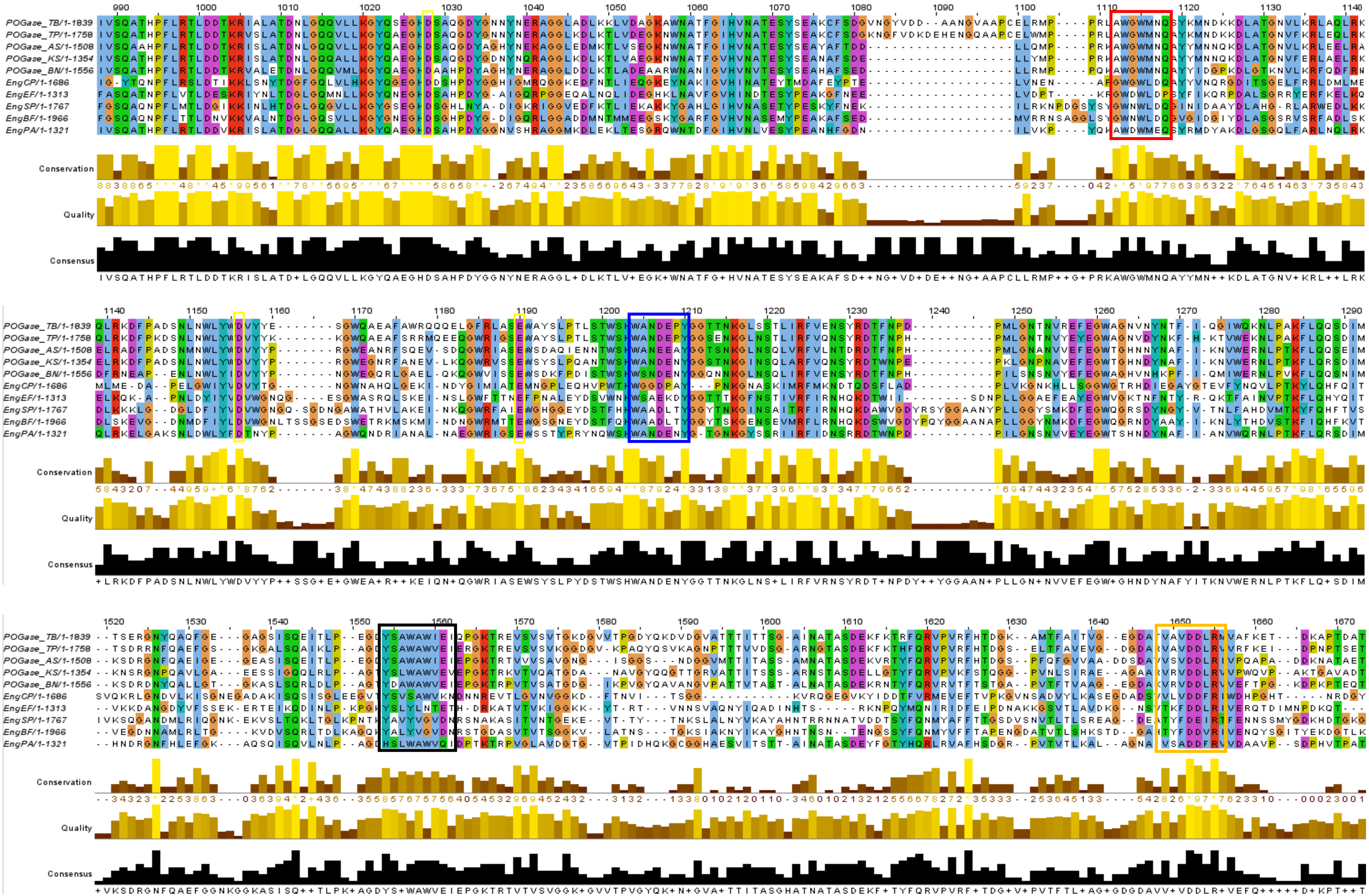

Supplementary Figure 9: Multiple sequence alignment of the GH101 Domain of 5 POGases and 5 known O-glycanases: Sequences were aligned with Clustal Omega and alignment was viewed using Jalview. For ease of analysis, residues were annotated and colored according to their clustal grouping. Motif-1 (red box), Motif-2 (blue box), and Motif -3 (black box) along with the DDE catalytic triad residues (yellow boxes) are all indicated. In orange, the transition point between GH101 domain and other domains is indicated. The quality (how likely a residue is found at all in that position), consensus (Sequence representing most common residue in aligned position) and conservation (how likely residues with similar properties are found in that position) is shown to show conservation/divergence.

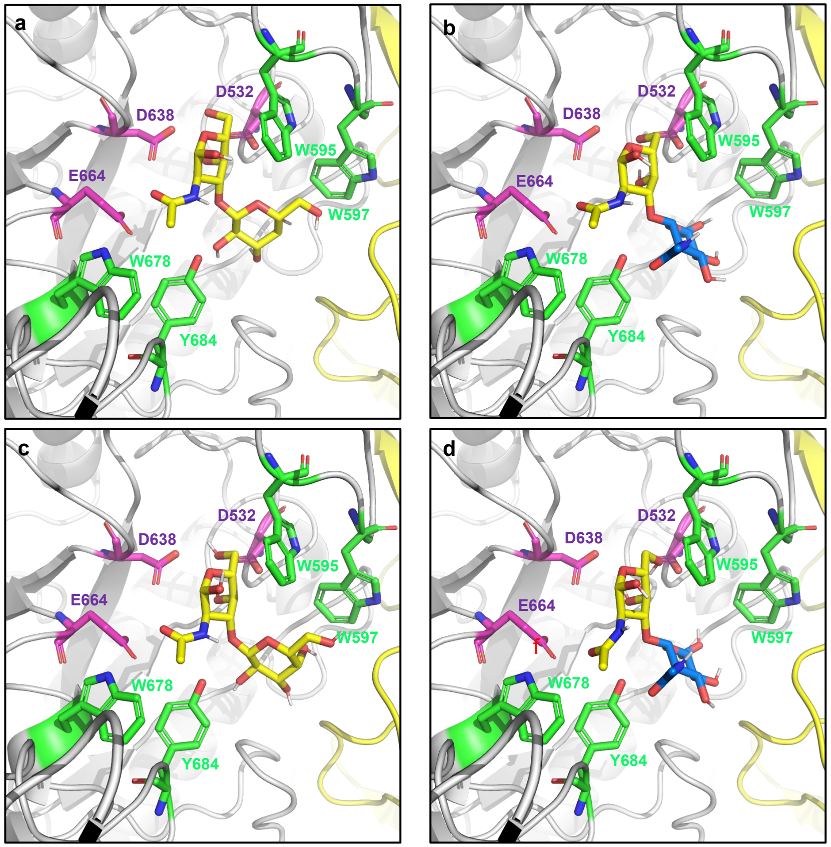

Supplementary Figure 10: Core 1 and Core 3 O-glycans docked to the active site of POGase AS—Comparing VinaCarb (a/b) and (c/d) AutoDock Results. a. Core 1 O-glycan (Yellow) docked between the WW/WY domains (Green) with the αGalNAc oriented toward the DDE catalytic triad (Magenta), docked with Vina Carb. **b.** Core 3 O-glycan (Yellow and Blue) docked between the WW/WY domains (Green) with the αGalNAc oriented toward the DDE catalytic triad (Magenta), docked with Vina Carb. **c.** Core 1 O-glycan (Yellow) docked between the WW/WY domains (Green) with the αGalNAc oriented near the DDE catalytic triad, docked with AutoDock Vina. **d.** Core 3 O-glycan (Yellow and Blue) docked between the WW/WY domains (Green) with the αGalNAc oriented near the DDE catalytic triad, docked with AutoDock Vina.

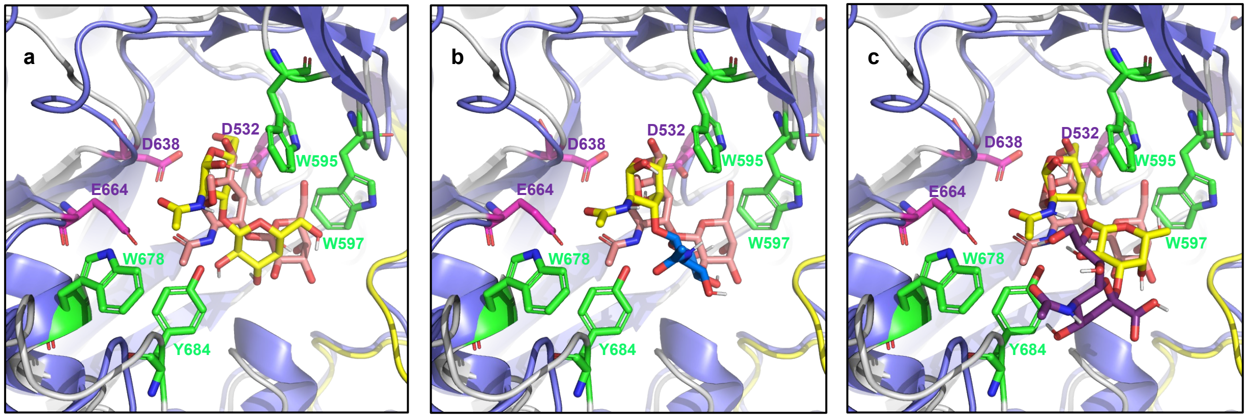

Supplementary Figure 11: Aligning Vina Carb Results to 5A56 reveals glycan docked poses aren't significantly altered from that of the experimentally derived structure with a T antigen (Core 1) agonist.

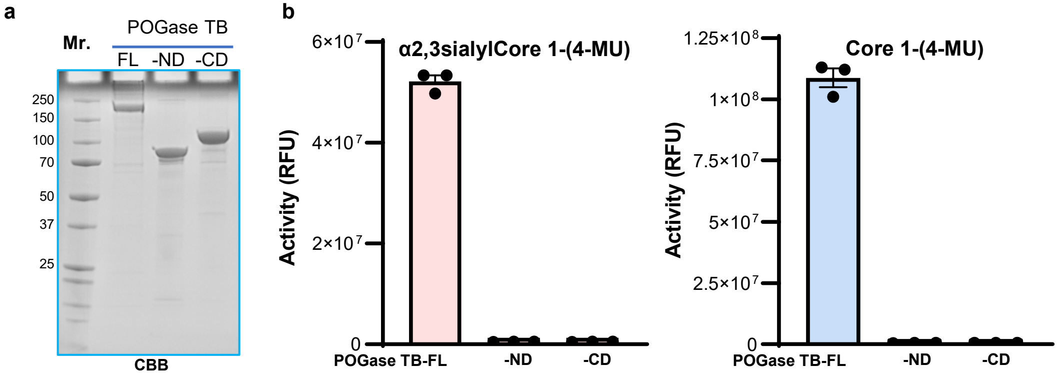

Supplementary Figure 12: Expression and enzymatic activity assays of POGase TB and its domains: a. Expression and purification of the full-length POGase TB (FL), its N-terminal domain (-ND), and C-terminal domain (-CD) were expressed in *E. coli* and purified. The purified proteins were run on SDS-PAGE, and gel was stained with Coomassie blue. **b.** Enzymatic activity Assay: the activity of recombinant POGase TB-FL and-ND, and –CD (500ng proteins) was assayed using 40μM of α2,3sialylCore 1-(4-MU) substrate (left panel) and Core 1-(4-MU) (right panel) for 1 hour (n=3) at 30 μL reaction mixtures, respectively. After adding 100 μL 1M Glycine-NaOH (pH10) (stop solution) and mixing well, the RFU of free 4-MU was measured in the umbelliferone mode using the platereader. Data are presented as mean values ± SEM (n=3). Source data are provided as a Source Data file.

Supplementary Table 1. Putative endo-α-N-Acetylgalactosaminidase candidates screened in this study

Candidate#	Organism	GenBank	Candidate#	Organism	GenBank
01	Clostridiales bacterium CHKCI001	SCJ97620.1	24	Actinomyces bovis	SPT54038.1
02	Clostridiales bacterium CHKCI001	CUN20024.1	25	[Ruminococcus] torques	CUN81354.1
03	Streptococcus acidominimus	SNV33305.1	26	uncultured Ruminococcus sp.	SCH51847.1
04	Streptococcus suis	CYV18626.1	27	Streptococcus pneumoniae	COG72222.1
05	Hungatella hathewayi	CUP62238.1	28	Streptococcus pneumoniae	COM85265.1
06	Streptococcus suis	CYU53783.1	29	Clostridiales bacterium CHKCI001	CVI65756.1
07	Streptococcus suis	CYV10025.1	30	Clostridiales bacterium CHKCI001	CVI65639.1
08	Streptococcus suis	CYV24804.1	31	Trueperella pyogenes	AZR00042.1
09	Streptococcus suis	CYV25301.1	32	Neoactinobaculum massilliense	WP_124039591.1
10	Streptococcus suis	CYV69930.1	33	Schaalia hyovaginalis	WP_154476515.1
11	Streptococcus suis	CYW34645.1	34	Actinomyces sp.	HHT41109.1
12	Streptococcus suis	CYV05123.1	35	Gleimia hominis	PMC85471.1
13	Streptococcus suis	CYX81346.1	36	Cutibacterium avidum	RFT46484.1
14	Bifidobacterium reuteri DSM 23975	KFI88282.1	37	Knoellia sp. DB2414S	WP_171241682.1
15	Bifidobacterium longum subsp. longum JCM 1217	AAX44931.1	38	Bowdeniella nasicola	WP_073716010.1
16	Bifidobacterium longum subsp. longum CECT 7347	CCK34131.1	39	Knoellia remsis	WP_106296018.1
17	Bifidobacterium longum subsp. suis	KFI72916.1	40	Tessaracoccus massiliensis	WP_052459929.1
18	Streptococcus pneumoniae	CKE04844.1	41	bacterium 1xD8-27	NBJ77735.1
19	Bifidobacterium tsurumiense	KFJ08221.1	42	Tessaracoccus rhinocerotis	WP_143938588.1
20	[Ruminococcus] torques	CUO40056.1	43	Brachybacterium tyrofermentans	WP_193115799.1
21	uncultured Ruminococcus sp.	SCI35173.1	44	Corynebacterium xerosis	SLM98462.1
22	Trueperella bernardiae	KTF04411.1	45	Cutibacterium acnes KPA171202	AAT83312.1
23	Bifidobacterium bombi DSM 19703	KFF31755.1	46	Brachybacterium alimentarium	RCS90382.1

Supplementary Table 2. POGases with significant activity to cleave α2,3sialylCore 1 O-glycans.

Name	Clone#	Organism	GeneBank ID
POGase TB	22	Trueperella bernardiae	KTF04411.1
POGase TP	31	Trueperella pyogenes	AZR00042.1
POGase AS	34	Actinomyces sp.	HHT41109.1
POGase KS	37	Knoellia sp. DB2414S	WP_171241682.1
POGase BN	38	Bowdeniella nasicola	WP_073716010.1

Supplementary Table 3: Glycopeptides Purchased from Vendors

Name	Sequences	O-glycans	Mass	Vendor
MUC5AC-3/13	GTT*PSPVPTTSTT*SAP-OH	GalNAcα- (Tn antigen)	1907.6	AnaSpec
CD24(41-51)-Tf(41, 51)	T*SNSGLAPNPT*-OH	Galβ1-3GalNAcα- (Core 1)	1788.77	Sussex Research
CD24(41-51)-sTf(41,51)	T*SNSGLAPNPT*-OH	Neu5Acα2-3Galβ1-3GalNAcα- (α2,3SialylCore 1)	2371.3	Sussex Research
MUC1(138-157)- Tn(144,150,151)	VTSAPDT*RPAPGS*T*APPAHG-OH	GalNAcα- (Tn antigen)	2496.59	Sussex Research

Supplementary Table 4. Comparisons of Motif-1, -2, and -3 sequences between POGases and known O-glycanases.

Name	Motif-1	Motif-2	Motif-3	Representative
POGases	AWGWMNQ	WANEEAY	YSAWAWV/IEI	POGase AS
O-glycanases	GWD/NWLDN	WSAEKDY	YSLYLNTET	EngEF

Supplementary Table 5: Comparison of the two algorithms for docking

O-Glycans	AutoDock Vina	Vina-Carb	Chi Energy Applied	Anomeric C to DDE triad (Å)
Core 1	-5.7 ± 0.06 kcal/mol	-5.5 ± 0.15 kcal/mol	0.0	6.0, 5.7, 7.1 (ns)
Core 3	-5.5 ± 0.06 kcal/mol	-5.5 ± 0.1 kcal/mol	0.0	6.4, 6.1, 7.5 (ns)
α2,3SialyCore 1	-	-5.3 ± 0.06 kcal/mol	4.5 ± 0.0	6.5, 5.6, 6.9 (ns)
5a56	-	-	-	5, 4.2, 6.4

Supplementary Materials:

Movie_1: Predicted 3D Structure of POGase AS

Movie_2: Evolutionary Coupled Residues in/or Near Motif 1 (AWGWMNQ) within POGase AS
