# Supplementary material for: Dual functional POGases from bacteria encompassing broader O-glycanase and adhesin activities
Source: Nat Commun. 2025 Feb 25;16:1960. doi: 10.1038/s41467-025-57143-8 (PMC11861894; doi:10.1038/s41467-025-57143-8)
Supplement: Supplementary file 2 — Description of Additional Supplementary Files [file 41467_2025_57143_MOESM2_ESM.pdf]

Description of Additional Supplementary Files

File Name: Supplementary Movie 1

Description: Predicted 3D Structure of POGase AS

AlphaFold2 Model shows predicted 3D structure of POGase AS around both the latitudinal and longitudinal faces. F5/8 Type C domains are in purple, GH101 domain is in white, GalBD domain is in yellow, unknown and unstructured domains are in Cyan. Residues of interest in the GH101 are either green (W595, W597, W678, and Y684) or pink (D532, D638, and E664). The movie was generated in PyMol v2.

File Name: Supplementary Movie 2

Description: Evolutionary Coupled Residues in/or Near Motif 1 (AWGWMNQ)

This movie shows the sidechains of evolutionarily coupled residues in the modeled POGase AS (M598/I429) and PDB ID: 5a56 (L726/F618). Measurements only shown for PDB ID: 5a56 is to show possible interaction points in the solved structure. POGase AS residues are in white and PDB ID: 5a56 residues are in blue. Parts of the yellow GalDB domain and Green W597 of POGase AS are observable and show the evolutionary coupled residues in context to other structural elements. The movie was generated in PyMol v2.
