# Supplementary material for: Dual functional POGases from bacteria encompassing broader O-glycanase and adhesin activities
Source: Nat Commun. 2025 Feb 25;16:1960. doi: 10.1038/s41467-025-57143-8 (PMC11861894; doi:10.1038/s41467-025-57143-8)
Supplement: Supplementary file 5 — Reporting Summary [file 41467_2025_57143_MOESM5_ESM.pdf]

nature portfolio

Corresponding author(s): NCOMMS-24-20598B

Last updated by author(s): Jan 10, 2025

Reporting Summary

Nature Portfolio wishes to improve the reproducibility of the work that we publish. This form provides structure for consistency and transparency in reporting. For further information on Nature Portfolio policies, see our Editorial Policies and the Editorial Policy Checklist.

Statistics

For all statistical analyses, confirm that the following items are present in the figure legend, table legend, main text, or Methods section.

n/a	Confirmed	
☐	☒	The exact sample size (*n*) for each experimental group/condition, given as a discrete number and unit of measurement
☒	☐	A statement on whether measurements were taken from distinct samples or whether the same sample was measured repeatedly
☒	☐	The statistical test(s) used AND whether they are one- or two-sided<br>*Only common tests should be described solely by name; describe more complex techniques in the Methods section.*
☒	☐	A description of all covariates tested
☒	☐	A description of any assumptions or corrections, such as tests of normality and adjustment for multiple comparisons
☐	☒	A full description of the statistical parameters including central tendency (e.g. means) or other basic estimates (e.g. regression coefficient) AND variation (e.g. standard deviation) or associated estimates of uncertainty (e.g. confidence intervals)
☒	☐	For null hypothesis testing, the test statistic (e.g. *F*, *t*, *r*) with confidence intervals, effect sizes, degrees of freedom and *P* value noted<br>*Give P values as exact values whenever suitable.*
☒	☐	For Bayesian analysis, information on the choice of priors and Markov chain Monte Carlo settings
☒	☐	For hierarchical and complex designs, identification of the appropriate level for tests and full reporting of outcomes
☒	☐	Estimates of effect sizes (e.g. Cohen's *d*, Pearson's *r*), indicating how they were calculated

Our web collection on statistics for biologists contains articles on many of the points above.

Software and code

Policy information about availability of computer code

Data collection	We downloaded sequences and databases used in this study by copying link addresses (provided in the manuscript) to the data directly from the host websites.
Data analysis	The following software and tools were used for analyses in alphabetical order with version in parentheses: AlphaFold (2.3.0), AutoDockVina (1.2.5), Vina-Carb, Glycam Carbohydrate builder (http://glycam.org), Clustal Omega (1.2.4), IQ-TREE (1.6.12), iTOL (6.0), Jalview (2.11.3.0), MAFFT (7.525), PyMOL (3.0), trimAl (1.2rev59), PDB ID:5a56 (https://www.rcsb.org/structure/5a56), GrapgPad Prosm 10.2.3 (403), and GlycoWorkBench version 2.1 (https://code.google.com/archive/p/glycoworkbench/)

For manuscripts utilizing custom algorithms or software that are central to the research but not yet described in published literature, software must be made available to editors and reviewers. We strongly encourage code deposition in a community repository (e.g. GitHub). See the Nature Portfolio guidelines for submitting code & software for further information.

Data

Policy information about availability of data

All manuscripts must include a data availability statement. This statement should provide the following information, where applicable:

- Accession codes, unique identifiers, or web links for publicly available datasets
- A description of any restrictions on data availability
- For clinical datasets or third party data, please ensure that the statement adheres to our policy

All data supporting the findings of this study are available within the manuscript and the Supplementary Information file, and Source Data file.

Research involving human participants, their data, or biological material

Policy information about studies with human participants or human data. See also policy information about sex, gender (identity/presentation), and sexual orientation and race, ethnicity and racism.

Reporting on sex and gender	N/A
Reporting on race, ethnicity, or other socially relevant groupings	N/A
Population characteristics	N/A
Recruitment	N/A
Ethics oversight	N/A

Note that full information on the approval of the study protocol must also be provided in the manuscript.

Field-specific reporting

Please select the one below that is the best fit for your research. If you are not sure, read the appropriate sections before making your selection.

[x] Life sciences   [ ] Behavioural & social sciences   [ ] Ecological, evolutionary & environmental sciences

For a reference copy of the document with all sections, see nature.com/documents/nr-reporting-summary-flat.pdf

Life sciences study design

All studies must disclose on these points even when the disclosure is negative.

Sample size	N/A
Data exclusions	N/A
Replication	N/A
Randomization	N/A
Blinding	N/A

Reporting for specific materials, systems and methods

We require information from authors about some types of materials, experimental systems and methods used in many studies. Here, indicate whether each material, system or method listed is relevant to your study. If you are not sure if a list item applies to your research, read the appropriate section before selecting a response.

Materials & experimental systems

n/a	Involved in the study
[ ]	[x] Antibodies
[ ]	[x] Eukaryotic cell lines
[x]	[ ] Palaeontology and archaeology
[x]	[ ] Animals and other organisms
[x]	[ ] Clinical data
[x]	[ ] Dual use research of concern
[x]	[ ] Plants

Methods

n/a	Involved in the study
[x]	[ ] ChIP-seq
[x]	[ ] Flow cytometry
[x]	[ ] MRI-based neuroimaging

Antibodies

Antibodies used	Alexa Fluor® 647-labeled anti-His Tag antibody (Cat# 801 652513, BioLegend); anti-His PAb; anti-Tn mAb (mouse IgM mAb, CA3638, BaGs6, In-house)
Validation	1. Alexa Fluor® 647-labeled anti-His Tag antibody has been routinely used in the GlycanArray analysis by the Emory Glycomics and Molecular Interactions Core (EGMIC) (https://www.cores.emory.edu/egmic/index.html).<br>2. Anti-Tn mouse IgM (CA3638) was validated by many studies, such as in the papers, PMID: 20439703, and PMID: 31742337.<br>3. Anti-6x-His Tag mAb (HIS.H8, Cat#MA1-21315-AF488) was validated by Thermo Fisher Scientific.

Eukaryotic cell lines

Policy information about cell lines and Sex and Gender in Research

Cell line source(s)	HEK293, ATCC# CRL-1573, ATCC.
Authentication	The HEK293 cell lines were authenticated using Short Tandem Repeat (STR) analysis and confirmed by the ATCC Cell Line Authentication Service.
Mycoplasma contamination	The cell lines were not tested for mycoplasma contamination yet.
Commonly misidentified lines (See ICLAC register)	The HEK293 cell lines are also used in our other studies, and their authentication was confirmed.

Plants

Seed stocks	N/A
Novel plant genotypes	N/A
Authentication	N/A
