# Supplementary material for: Dual functional POGases from bacteria encompassing broader O-glycanase and adhesin activities
Source: Nat Commun. 2025 Feb 25;16:1960. doi: 10.1038/s41467-025-57143-8 (PMC11861894; doi:10.1038/s41467-025-57143-8)
Supplement: Supplementary file 9 — Source Data 3 [file 41467_2025_57143_MOESM9_ESM.pdf]

nature portfolio

Peer Review File

Dual Functional POGases from Bacteria Encompassing Broader O-glycanase and Adhesin Activities

Corresponding Author: Dr Tongzhong Ju

This file contains all reviewer reports in order by version, followed by all author rebuttals in order by version.

Attachments originally included by the reviewers as part of their assessment can be found at the end of this file.

Version 0:

Reviewer comments:

Reviewer #1

(Remarks to the Author)
This work highlights the utility of a novel O-glycanase for O-glycomics and function as adhesins. The researchers characterized the binding activity of the novel POGase as well as the rate of deglycosylation. Initially 5 POgases were characterized and identified, however one POGase (POGase AS) was the standout enzyme in the group. This POgase was the focus of majority of the paper. One of the stand-out aspects of this enzyme is the ability to remove sialylated core1 and core 2 O-glycans, which most currently available commerical O-glycanases can not do. I believe the identification and characterization of this novel POGase will be extremely beneficial to the the field of glycobiology and glycochemistry. However the paper does require some editing before it should be accepted for publication.

1. The in-text citations appear within the period, when they should be outside of the period when in superscript form. This needs to be fixed throughout the article

2. A general proof reading is needed. There are multiple cases where a period, space, or comma are missing. There are also multiple points throughout the paper where one sentence will switch from singular to plural tense (The last sentence of the introduction is an example of this. It starts mentioning multiple POGases, then says "has" instead of "have", and then sentence ends with no period).

3. multiple times it is mentioned how the POGases can remove glycans from "glycopeptides, glycoproteins and therapeutic glycoproteins" I understand the clarification between glycopeptides and glycoproteins, however why is it necessary to clarify therapeutic glycoproteins vs non? What structurally would make this necessary to clarify?

4. When discussing the deglycosylation abilities of the POGases for glycomics analysis purposes, the term "native peptide" is used to describe the non-glycosylated peptide. This is very confusing. Since these proteins are glycosylated in nature, and in many cases the native form is glycosylated, this is not the best term choice in my opinion. Using "deglycosylated peptide", "non-glycosylated peptide", "unmodified peptide: would all be better options

5. When discussing the Muc5Ac and CD24 peptides, multiple times the term "a2,3sialylcore 2" is used, however there are multiple points where the authors discuss using MSMS to confirm the glycan structures. Do you know the sialic acid is 2,3 linked because the peptides were purchased and characterized or because MSn experiments were conducted? If they were not conducted, how do you know the sialic acid is 2,3 linked?

6. The terms LIFT-MS and MALDI-TOF-MS need to be defined

7. When comparing enzymatically released O-glycans from beta eliminated, the beta eliminated glycans were left as free ends, and the POGase glycans were reduced. This is said to be to help distinguish them, but why not reduce both? Are you releasing them from the same aliquot of protein...? Why do you need to distinguish them when they are processed separately? To me, it makes the most sense to have both in reduced form, as it will ensure everything besides the release mechanism are the same. It will also make sure ionization efficiency is not effected.

8. Did you attempt any deglycosylation studies on complex samples? Though this enzyme will be useful for O-glycomics of simple purified proteins, I find it difficult to find the validity in this helping with complex samples. Majority of researchers will have a greater need for de-o-glycosylating complex samples.

9. You have a figure showing the effects of the POgase when the protein has been denatured vs not, which in my opinion is worth noting but I am not sure how novel that is to include within a main text figure. Instead it might be a better option to compare the glycopeptide to the glycoprotein. I.e. for fetuin compare the results of the POGase on intact fetuin vs fetuin peptides.

Reviewer #2

(Remarks to the Author)
Zhou and colleagues have successfully screened a wide variety of putative POGases and found one in particular that was relatively fast and had broad-specificity. The overall presentation of the work is good and easy to interpret (I have included a few points on figures), the order of the different aspects is also logical. The writing is largely clear and provides easy understanding for the reader. I have no major issues, but a few queries/comments.
• How did the authors pick these enzymes and why? It would be interesting to know where the 30 you screened plus all the characterised ones sit in a phylogenetic tree of all GH101s. I think this would be more insightful than Figure 9.
• Line 99 – is that the activity of the GH16 enzymes?
• Figure 7 – annotate the different colours in a and the sugars in b. What are the salmon-coloured residues. Personally, I don’t like the black background, but that’s being picky.
• Line 396 – what are blood coagulation factors, why is it relevant?
• I’m not clear on your use of the term ‘O-glycanase’ throughout – are you referring to GH101 enzymes? I think, from the way you write, you want the POGases to be a class within GH101 enzymes? If you take the sequences of just the catalytic domains GH101 enzymes, do the POGases group together on a phylogenetic tree?
• Figure 8 – N-glycans have beta-1,4-galactose, not beta-1,3
• Figure 9 – I think this figure need work to make it useful. I’m not sure what the colours are.
• Line 492 – I think this starting sentence contradicts what the rest of the paragraph says. Do you mean that O-glycanases have motifs that are also in other enzymes? I’m not sure.
• Line 518-9 – a bit of repetition here. I’m not sure how there will be the development of therapeutic O-glycoproteins. In this paragraph, how would a GH101 be able to accommodate much longer O-glycans? I had a quick look at the Strep pneumo structure with only the catalytic domain and there’s nothing stopping a longer O-glycan being accommodated in theory. How does your model compare to this GH101?
• Paragraph line 536 – I think the binding data is good, but I think it’s a step too far to talk about disease if these enzymes haven’t been linked. This paragraph is a bit speculative.
• Paragraph 571 – where is this data? I think you need to show a tree of GH101s if you are going to say this

Reviewer #3

(Remarks to the Author)
In the manuscript by Zhou et al., the authors characterize a previously unstudied family of glycan hydrolases they term “POGases”. These enzymes are capable of releasing O-glycan structures that are sialylated and can operate on synthetic glycopeptides as well as therapeutically relevant glycoproteins. The authors perform a thorough investigation and MS-based analysis using POGase AS. They also perform phylogenetic and structural studies to understand why these enzymes are capable of releasing sialylated O-glycans while other known GH101-members are not. Since a universal O-glycanase is not known and highly valuable in glycobiology, this is a major contribution to the field. Overall, the authors present a very thorough, detailed, and well-written manuscript that I largely support. That said, the paper does have a few areas for improvement.

Major concerns
• The figure quality throughout the manuscript is very low. Many of the spectra/fonts/numbers are completely illegible and thus difficult to interpret. Several spectra have unannotated peaks. Many images are low resolution.
• For Figure 4C-E, the enzymatic release is incomplete. Did the authors attempt to change the time of reaction, concentration of enzymes, etc. to optimize full release of the glycans? If not, I would recommend attempting this to maximize utility of the enzyme.
o Related to the same figure, can the authors quantify these peaks/efficiency of glycan release?
o Several other species are in the spectra, what are these? If they cannot be identified by intact mass, which is common, perhaps label them as such?
• In Figure 5, I have a similar comment – what are the unannotated peaks? Also, since Fig 4b is MALDI of released glycans, would it not make sense to move 4b to figure 5?
• Extended Data 6 and 7 are very hard to read. Why are 6d/e so noisy? Is the lower signal due to the lower efficiency of glycan release?
• In Figure 6, what are the unannotated high and low mass peaks? Why are the high mass peaks in all three proteins?
o A discussion on the three proteins and their expected glycan structures would be useful. Fig 6b makes it appear as if there is only one glycan structure on all of the proteins, but this would be unusual. Even in CHO-produced proteins, some di-sialylated core 1 structures would be expected.
o What kind of excipients are present in the abatacept solution? Would it be possible to buffer exchange these contaminants out of solution and repeat the experiment?

o Fig 6c. – please quantify the differences between beta-eliminated/POGase released glycans.
• Figure 8 is difficult to read/decipher. Can the text be limited or removed for clarity?

Minor comments
• “Despite the formation”… “lack a common core” – this seems contradictory. It makes sense that if there are 20 GalNAcTs that the downstream processing will also be similarly convoluted. I would re-word this sentence.
• The authors mention a commercially available O-glycanase early in the paper but cite an academic laboratory. Later, they mention NEB as the manufacturer, but it would be helpful to mention at the first reference of the enzyme.
• A brief, 1-2 sentence introduction to LIFT-MS would be useful.
• Line 337 – the authors say “POGases” but really only seem to fully characterize AS. Might be useful to clarify this here.
• Why was POGase TB chosen for the N vs C terminal studies?
• Throughout the document, the authors refer to the enzymes as “novel”, but this is slightly odd language for enzymes that have existed throughout evolution. Perhaps “previously uncharacterized” is more accurate?
• The authors mention “complex O-glycans” but “complex” is often reserved for N-glycan structures. I believe the authors mean sialylated O-glycans, so this wording should be altered.

Reviewer #4

(Remarks to the Author)
In this manuscript, the authors identify a family of enzymes, which they term peptide:O-glycosidases (POGase), that are capable of cleaving substrates containing glycans with an initiating GalNAc residue. Some enzymes were found to cleave substrates with different core structures (1-3), including those modified with sialic acid. The enzymes appear to have broader glycan tolerance than related glycoside hydrolases (GHs) characterized to date. Phylogenetic analysis and modeling were performed to help rationalize this observation. Finally, one enzyme was found to bind specific structures in the glycan-binding array.

A universal O-glycan hydrolase is not known, as most GHs do not act broadly on all O-glycans. POGases with broader substrate tolerance could help fill that void and be of significant use to the field. That being said, I believe the authors need additional/improved studies to better characterize these enzymes and demonstrate such utility/impact before publication is warranted. As my background is primarily with modeling, my comments will largely focus on that portion of the study.

Major Critiques:

1. While the authors emphasize the importance of mucin-type glycosylation, the protein-based substrates (fetuin and etanercept/abatecept) are sparsely glycosylated and/or have a very small mucin-domain. To better understand the substrate tolerance/preference of these enzymes (which the authors admit may have some steric limitations), the authors should characterize activity on a recombinant mucin or larger mucin-domain-containing protein.

2. Furthermore, the authors should benchmark better against GHs (i.e. the used NEB O-glycosidase) on the synthetic glycopeptides and glycoproteins (beyond just fetuin, which does not contain a mucin-domain). Without doing this, it is difficult to appreciate the utility of POGases as a tool for glycobiology… which is pivotal to demonstrating the advantage of POGases over currently used GHs.

3. The glycan array data is overinterpreted: there’s no direct data demonstrating cellular adhesion. Accessory domains in some O-glycoproteases/mucinases have been suggested (and even observed in Glycobiology 2021, 31, 385) to be involved in secondary recognition of larger mucin substrates. That the N-terminal domain and the C-terminal domain constructs lack enzymatic activity (Extended Figure 10) suggests that the overall fold or cooperativity between the two domains has been lost. Regardless, it’s difficult to ascertain if these or some other mechanism is at play without doing larger structural study.

Modeling Critiques:

The modeling work follows standard protocols to generate model structures (AlphaFold2) and perform docking (AutoDock). The generated enzyme structure appears to have undergone quality control. The docking, by contrast, has not gone through sufficient quality control/analysis.

The current docked poses (Figure 7 & Extended Data Figure 9) have some questionable results, including several instances where (1) the anomeric carbon of the GalNAc residue is distant from the DDE catalytic triad and (2) there are high-energy cis amide bonds in the NAc moiety and ring flips with some sugars. Additionally, the GalNAc and Gal moieties are oriented with their nonpolar faces away from the WW motif (preventing CH-pi stacking), which is unexpected given (a) what is known about GalNAc recognition (Acc. Chem. Res. 2023, 56, 548) and – more importantly – (b) poses of glycans observed in X-ray structures of other GH101-containing enzymes (J. Biol. Chem. 2015, 290, P25657). As such, I doubt that any of the docked poses reflect the actual binding pose of glycans.

The authors should redo the docking studies, ideally using an enzyme-T antigen cocrystal structure from the aforementioned JBC article as a guide to initially place the T-antigen into active site of the modeled enzyme. This can be docked using AutoDock. From there, the other core structures can be easily generated: modifying sugars of the docked T-antigen and/or “grafting” other crystallographic sugars (which can be searched using http://glycanstructure.org/fragment-db) onto the T-antigen core and generating a small library in which the new sugars are allowed to adopt different conformations. Each

conformer library can then be docked using AutoDock as before and you can get improved results. This process will help ensure that docking begins with low energy ligand conformers placed in approximately the “right” place within the pocket. Yes, it introduces bias into the system, but it is at least grounded in prior results and allows for more meaningful data interpretation.

As it stands, the docking doesn’t add much to the story of the paper. I would recommend the authors (1) compare their docked structures to the JBC crystal structures to better understand why the POGases permit larger and more diverse glycans than GHs and (2) possibly dock glycans into modeled structures of POGases that are less active or more selective to better understand these differences, as the authors suggest that these enzymes could be engineered for better results.

Finally – partially because the coloring between Figure 7a and 7b are inconsistent – it remains unclear how the conserved, ligand-binding residues of the F5/8 type C domains (which can probably be determined using UniProt) spatially relate to the active site. Again, it is very well possible that the noncatalytic domains participate cooperatively in binding larger, densely glycosylated substrates like mucins rather than engaging in cellular adhesion, as the authors have implied.

Version 2:

Reviewer comments:

Reviewer #1

(Remarks to the Author)
The manuscript was edited and modified based on the reviewers comments. This manuscript was enhanced by adding additional explanations of techniques used, as well as additional explanations of the data and results. Though the information added is rich, the general grammar in these new sections is still lacking. A detailed proof read is still needed before this manuscript can be accepted.

Minor comments:
The sentence on page 11, line 320 needs to be reworked, please find my suggested sentence structure "Consistent with Figure 6a, three major unreduced
O-glycans were identified in Fetuin (mono-sialylCore 1: m/z 879; di-sialylCore 1: m/z 1240; and
di-sialylCore 2: m/z 1689) (Figure 6c top panel). However, in abatacept and etanercept, only two O-glycan species were identified from β-elimination reactions, which included mono-sialylCore 1 (m/z 879) and di-sialylCore 1(m/z 1240) (Figure 6d, and 6e top panel), which is consistent with the observations made in our previous study with the same glycoproteins30.

page 11 line 333 "tandem mass spectra were only possible..." were should be was. This issue repeats at line 340.

page 11 line 344 first sentence needs proof reading

page 16 line 474, HEK293 doesn't need to be defined twice

The paragraph added on page 23, line 683 seems unusual to me. I appreciate referencing those who helped with piecing together the data and it's meaning, however I am not sure this is a conventional way of representing this.

Figures:
Overall the clearness of the figures has significantly increased, however the color scheme used to represent the glycans does not correspond with SNFG, and is honestly quite harsh to look at. Please update colors as defined by SNFG (https://www.ncbi.nlm.nih.gov/glycans/snfg1_5.html table 2)

Reviewer #2

(Remarks to the Author)
The manuscript has improved significantly. Just two points:
I'm not sure the second sentence of the intro makes sense.
Sialic acid should be pinky purple, not blue - maybe this came out wrong in figure 1?

Reviewer #3

(Remarks to the Author)
The authors have addressed all of my comments and I have no further suggestions.

Reviewer #4

(Remarks to the Author)
Please see separate "Response to Rebuttal" file for full comments.

Open Access This Peer Review File is licensed under a Creative Commons Attribution 4.0 International License, which permits use, sharing, adaptation, distribution and reproduction in any medium or format, as long as you give appropriate credit to the original author(s) and the source, provide a link to the Creative Commons license, and indicate if changes were made.
In cases where reviewers are anonymous, credit should be given to 'Anonymous Referee' and the source.
The images or other third party material in this Peer Review File are included in the article's Creative Commons license, unless indicated otherwise in a credit line to the material. If material is not included in the article's Creative Commons license and your intended use is not permitted by statutory regulation or exceeds the permitted use, you will need to obtain permission directly from the copyright holder.
To view a copy of this license, visit https://creativecommons.org/licenses/by/4.0/

RESPONSES TO REVIEWER COMMENTS

Nature Communications: Manuscript NCOMMS-24-20598-T

Reviewer #1 (Remarks to the Author):

This work highlights the utility of a novel O-glycanase for O-glycomics and function as adhesins. The researchers characterized the binding activity of the novel POGase as well as the rate of deglycosylation. Initially 5 POgases were characterized and identified, however one POGase (POGase AS) was the standout enzyme in the group. This POgase was the focus of majority of the paper. One of the stand-out aspects of this enzyme is the ability to remove sialylated core1 and core 2 O-glycans, which most currently available commerical O-glycanases can not do. I believe the identification and characterization of this novel POGase will be extremely beneficial to the field of glycobiology and glycochemistry. However the paper does require some editing before it should be accepted for publication.

1. The in-text citations appear within the period, when they should be outside of the period when in superscript form. This needs to be fixed throughout the article.

***Response*:** We thank the Reviewer's comment. We carefully looked at the published articles, the citations are within the period.

2. A general proof reading is needed. There are multiple cases where a period, space, or comma are missing. There are also multiple points throughout the paper where one sentence will switch from singular to plural tense (The last sentence of the introduction is an example of this. It starts mentioning multiple POGases, then says "has" instead of "have", and then sentence ends with no period).

***Response*:** We thank the Reviewer's critical reading. We have made corrections of those grammatical errors and typos in this revised manuscript.

3. multiple times it is mentioned how the POGases can remove glycans from "glycopeptides, glycoproteins and therapeutic glycoproteins" I understand the clarification between glycopeptides and glycoproteins, however why is it necessary to clarify therapeutic glycoproteins vs non? What structurally would make this necessary to clarify?

***Response*:** We thank the Reviewer's comment. This is an emphasis of our research as we work at the US FDA in regulating the therapeutic glycoproteins. We changed the sentence to "glycopeptides, glycoproteins, including therapeutic glycoproteins" in the revised manuscript.

4. When discussing the deglycosylation abilities of the POGases for glycomics analysis purposes, the term "native peptide" is used to describe the non-glycosylated peptide. This is very confusing. Since these proteins are glycosylated in nature, and in many cases the native form is glycosylated, this is not the best term choice in my opinion. Using "deglycosylated peptide", "non-glycosylated peptide", "unmodified peptide: would all be better options.

***Response*:** We thank the Reviewer's comment. We agree with the Reviewer. We changed the "native peptide" to "unmodified peptide".

5. When discussing the Muc5Ac and CD24 peptides, multiple times the term "a2,3sialylcore 2" is used, however there are multiple points where the authors discuss using MSMS to confirm the glycan structures. Do you know the sialic acid is 2,3 linked because the peptides were purchased and characterized or because MSn experiments were conducted? If they were not conducted, how do you know the sialic acid is 2,3 linked?

Response: We understand the Reviewer's comment. α2,3sialylcore 2-glycopeptides were made synthesized in-house using step-by-step recombinant glycan extension with recombinant glycosylatransferases and donor substrates. Specifically we used ST3Gal1 and CMP-sialic acid to generate α2,3sialyl Core 1, and Core 2 from synthetic Core 1, Core 2 MUC5AC-glycopeptides to make the α2,3sialylation. The purpose of the MS/MS experiments were to demonstrate that POGase AS could release a variety of intact O-glycans, based on distinct permethylated glycan fragments observed in the tandem mass spectra (Extended Figure 6).

6. The terms LIFT-MS and MALDI-TOF-MS need to be defined

Response: We understand the reviewer's comment on defining LIFT-MS and MALDI-TOF-MS in the manuscript. MALDI-TOF-MS is the abbreviation for matrix assisted laser desorption ionization time of flight mass spectrometry, is a class of mass spectrometry which is commonly used in the field for analysis of permethylated glycans. LIFT is a proprietary technique from Bruker molecular ions are initially accelerated at lower energy, then collide with inert gas in the collision cell that is then lifted to high potential. The MALDI-TOF-MS in our lab has the capability to switch between collision gas to generate fragmentation products, or laser induced dissociation (LID) for fragmentation (PMID:12830354). Fragments generated by LID can also undergo acceleration at lower energy and lifted to high potential through the collision cell. The manuscript has been updated to define MALDI-TOF-MS, and LIFT-MS was changed to tandem mass spectra across the manuscript.

7. When comparing enzymatically released O-glycans from beta eliminated, the beta eliminated glycans were left as free ends, and the POGase glycans were reduced. This is said to be to help distinguish them, but why not reduce both? Are you releasing them from the same aliquot of protein...? Why do you need to distinguish them when they are processed separately? To me, it makes the most sense to have both in reduced form, as it will ensure everything besides the release mechanism are the same. It will also make sure ionization efficiency is not effected.

Response: We note the reviewer's important comment on reducing O-glycans released by β-elimination. The β-eliminated O-glycans were prepared using our optimized one pot glycomic method (Ortega-Rodriguez et al, *Cell Reports Methods*, 2024, DOI: 10.1016/j.crmeth.2024.100834) in which short O-glycopeptides are first generated by proteinase K, and then O-glycans are released though a principle of β-elimination in the presence of NaOH during solid-phase permethylation. The resultant free O-glycans with free reducing ends are immediately methylated as described. Our method does not only efficiently release all O-glycans but also possesses undetectable peeling. This approach is standard practice in our group, which is why the O-glycans released by β-elimination were released in accordance with our method.

8. Did you attempt any deglycosylation studies on complex samples? Though this enzyme will be useful for O-glycomics of simple purified proteins, I find it difficult to find the validity in this helping with complex samples. Majority of researchers will have a greater need for de-o-glycosylating complex samples.

Response: We understand the Reviewer's point. We have tried the O-glycan release from more complex samples such as cell extracts. At this time, we have not worked out an optimum condition to release, recover and analyze the O-glycans from digestion of cell extracts using mass spectrometry, probably due to the complexity of samples in nature, and importantly the limited accessibility of POGase to mucin-domains of glycoproteins from cell extracts which is the common obstacle for O-glycomics. We continue investigating this. We have included this in our revised manuscript. Nevertheless, besides the limitation, our work has the following merits:

1) This is the first identification of naturally occurring enzyme with significant activity to cleave the most common O-glycans, α2,3sialylCore 1, and residual activity to cleave more complex sialylated O-glycans, such as di-sialylCore 1 and Core 2.
2) We have shown that POGase AS can release intact sialylCore 1 and others from glycoproteins, fetuin and 2 therapeutic glycoproteins.
3) This work has demonstrated that the POGase has potential adhesin activity in addition to its enzymatic activity.
4) This work has a significant implication to the microbiology, for example, how the POGases enable bacteria to colonize, and its potential roles in attachment/invasion of pathogens or opportunist pathogens.

Overall, this work is not about biotechnology or tool development. As we discussed, the POGase AS is not a perfect tool yet, however, this work is a step forward to potentially developing a feasible biotechnology/tool for O-glycomics. We believe the audiences will appreciate the study, and more importantly, it will also impact numerous scientific disciplines including glycobiology, biotechnology, microbiology, immunology, and medicine. We respect the Reviewer's comment, we are conducting the study and will be reporting in a different manuscript soon.

9. You have a figure showing the effects of the POgase when the protein has been denatured vs not, which in my opinion is worth noting but I am not sure how novel that is to include within a main text figure. Instead it might be a better option to compare the glycopeptide to the glycoprotein. I.e. for fetuin compare the results of the POGase on intact fetuin vs fetuin peptides.

Response: We understand the Reviewer's comment. Our figures have been reformatted to focus only on O-glycans released by POGase from denatured proteins, and O-glycopeptides generated by Proteinase K-. Reformatted figures to focus only on the protein vs peptides (new figure 6). Additionally, we have added profiles of O-glycans released by β-elimination (using our optimized one pot glycomic method) above the spectra of O-glycans released by POGase from glycopeptides and denatured fetuin, abatacept, and etanercept. The purpose of this change is to emphasize that POGase is capable of releasing most of the O-glycans, but the enzyme prefers to cleave 2,3sialylCore 1 O-glycans over other more sialylated O-glycan species.

Reviewer #2 (Remarks to the Author):

Zhou and colleagues have successfully screened a wide variety of putative POGases and found one in particular that was relatively fast and had broad-specificity. The overall presentation of the work is good and easy to interpret (I have included a few points on figures), the order of the different aspects is also logical. The writing is largely clear and provides easy understanding for the reader. I have no major issues, but a few queries/comments.

• How did the authors pick these enzymes and why? It would be interesting to know where the 30 you screened plus all the characterised ones sit in a phylogenetic tree of all GH101s. I think this would be more insightful than Figure 9.

Response: We understand the Reviewer's comment. Initially, we selected the candidates for testing their potential activity to cleave sialylCore 1-(4-MU) based on these thoughts: 1) predicted to be an endo-α-N-acetyl-β-D-galactosaminidase from BRENDA database; 2) Previously uncharacterized candidates from different bacteria; 3) large proteins, >1,500aa long. Now we know all 30 enzymes plus characterized ones

belong to GH101, a bigger phylogenetic tree, but the POGases with activity to cleave sialylated O-glycans grouped in a monophyletic clade contained within the GH101 phylogenetic tree, specifically within the group of GH101 enzymes expressed in *Actinomycetes* in Fig. 10 in the revised manuscript.

• Line 99 – is that the activity of the GH16 enzymes?

Response: Yes, the enzyme identified in the work was a member of GH16. It is not an O-glycanase, is actual endo-β-N-acetyl-D-glucosaminidase. The term, O-glycanase in that paper confuses readers.

• Figure 7 – annotate the different colours in a and the sugars in b. What are the salmon-coloured residues. Personally, I don't like the black background, but that's being picky.

Response: We agree with the Reviewer's suggestion, and have changed colors to match in each modeling data with white background in our revised manuscript.

• Line 396 – what are blood coagulation factors, why is it relevant?

Response: We understand the Reviewer's comment. The 5C/F Domain was also found the blood coagulation factors, it is irrelevant to this work. We deleted it from this revised manuscript.

• I'm not clear on your use of the term 'O-glycanase' throughout – are you referring to GH101 enzymes? I think, from the way you write, you want the POGases to be a class within GH101 enzymes? If you take the sequences of just the catalytic domains GH101 enzymes, do the POGases group together on a phylogenetic tree?

Response: We note the Reviewer's comment. We use the term 'O-glycanase' throughout to refer to GH101 enzymes. The newly identified POGases are a class within GH101 enzymes, and grouped in a monophyletic clade on the GH101 phylogenetic tree (Fig. 10). Based on the published and our current ongoing studies, it appears that the GH101 family include various sub-families of enzymes, with each sub-family processing distinct enzymatic/substrate specificity. While POGases are only expressed in one phylum, the GH101 family seems to express a wide diversity of subclasses, but that information is outside the scope of this current study. We are now investigating and will continue reporting them.

• Figure 8 – N-glycans have beta-1,4-galactose, not beta-1,3.

Response: We understand the Reviewer's comment. In N-glycans, typically beta1,4-Galactose is attached to N-Acetylglucosamine, known as Type-II chain. We carefully took a look at the GlycanArray results, and found that both full-length and C-terminal domain of POGase bind to the bivalent Galbeta1,3GlcNAc-, the Type-I chains on N-glycans, but not Type-II chains. The GlycanArray used in this study is composed of known glycan structures synthesized chemically and enzymatically, including Type-I chains on N- and O-glycans, and glycolipids (PMID: 21488041).

• Figure 9 – I think this figure need work to make it useful. I'm not sure what the colours are.

Response: We apologize for not including the color codes. We added clarifying text in the article and a key for the colors in the revised figure.

• Line 492 – I think this starting sentence contradicts what the rest of the paragraph says. Do you mean that O-glycanases have motifs that are also in other enzymes? I'm not sure.

Response: We thank the Reviewer's critics. We revised the starting sentence as " POGases have both unique and shared sequence motifs compared to known O-glycanases."

• Line 518-9 – a bit of repetition here. I'm not sure how there will be the development of therapeutic O-glycoproteins. In this paragraph, how would a GH101 be able to accommodate much longer O-glycans? I had a quick look at the Strep pneumo structure with only the catalytic domain and there's nothing stopping a longer O-glycan being accommodated in theory. How does your model compare to this GH101?

Response: We are sorry for the confusion caused by lack of clarity in the manuscript. We address this by discussing the flexibility of the POGase specific motifs as well as the inclusion of the hydrophobic core interactions compared to the known O-glycanses. We also hypothesize that charge states of the motifs might contribute to the increase in substrate pools for POGases. To summarize, as discussed in the main text, the flexibility of the motifs, the change in hydrophobic core interactions, and the change in charge states are hypothesized to play a role in increased substrate pool, but more experiments need to be done to verify in future studies. As for the development of therapeutic O-glycoproteins, we meant that a universal POGase can make it possible for the characterization and control of O-glycosylation of therapeutic O-glycoproteins, thus facilitate the drug development.

• Paragraph line 536 – I think the binding data is good, but I think it's a step too far to talk about disease if these enzymes haven't been linked. This paragraph is a bit speculative.

Response: We respect the Reviewer's comment. To further prove the adhesin activity of POGase, we performed cell-binding experiments to show that the full length POGase, and its C-terminal domain, but not the N-terminal domain of POGase TB can specifically bind to a human cell line, HEK293 cells mainly after de-sialylation. Regarding its role in human diseases, one study showed that the EngCP, a known O-glycanase from *Clostridium perfringens* is a virulence factor involved in gangrene infections. Based on this finding, we speculate the pathogenic role of POGases in human and animal diseases, raise the alarm to the relevant field to pay attention and investigate the potential role of these POGases in diseases.

• Paragraph 571 – where is this data? I think you need to show a tree of GH101s if you are going to say this

Response*:* We thank the Reviewer's comment. We clarified to demonstrate that the tree being shown is the GH101s isolated from the database tree we generated (Fig. 10) in the revised manuscript.

Reviewer #3 (Remarks to the Author):

In the manuscript by Zhou et al., the authors characterize a previously unstudied family of glycan hydrolases they term "POGases". These enzymes are capable of releasing O-glycan structures that are sialylated and can operate on synthetic glycopeptides as well as therapeutically relevant glycoproteins. The authors perform a thorough investigation and MS-based analysis using POGase AS. They also perform phylogenetic and structural studies to understand why these enzymes are capable of releasing sialylated O-glycans while other known GH101-members are not. Since a universal O-glycanase is not known and highly valuable in glycobiology, this is a major contribution to the field. Overall, the authors present a very thorough, detailed, and well-written manuscript that I largely support. That said, the paper does have a few areas for improvement.

Major concerns

• The figure quality throughout the manuscript is very low. Many of the spectra/fonts/numbers are completely illegible and thus difficult to interpret. Several spectra have unannotated peaks. Many images are low resolution.

Response: We thank the Reviewer's comment. The low quality of the figures was partly due to the embedding of figures into the text in the pre-submission to *Nature*, which was then directly transferred to *Nature Communications* for your review. We have revised our figures in high resolution, and labeled the unidentified peaks in MS spectra.

• For Figure 4C-E, the enzymatic release is incomplete. Did the authors attempt to change the time of reaction, concentration of enzymes, etc. to optimize full release of the glycans? If not, I would recommend attempting this to maximize utility of the enzyme.

Response: We thank the Reviewer for this important note. We attempted several approaches to get full optimized release, including using excess enzyme, different buffers, different incubation times, and we did not observe complete release. Most likely a protein stability issue which that could we plan to be address ed with precise, in-depth formulation studies in a future paper. It could also be due to the significant inhibition by the product. We are now investigating these and get an optimum condition to efficient release of sialylated O-glycans.

- Related to the same figure, can the authors quantify these peaks/efficiency of glycan release?

  Response: We agree with the Reviewer, that it is important to quantity the peaks observed in both the controls, and digested samples, in order to understand the efficiency of glycan release with POGase AS. The extracted ion chromatograms (XICs) of each peak were extracted using a mass tolerance of 0.0500 dalton, to facilitate high accuracy mass filtering. The XIC peak area exported % peak area of each substrate peak, and product peak was summarized in a Fig. 6b, and Extended Data figure 6 to provide with %Peak area. The data clearly shows the difference in product peaks after POGase treatment (relative abundance based on extracted ion chromatograms (XIC) of each product peak, underscoring that POGase can fully release 2,3sialylated Core 1, and Core 2 O-glycans, with preference to 2,3sialylCore 1.

- Several other species are in the spectra, what are these? If they cannot be identified by intact mass, which is common, perhaps label them as such?

  Response: As noted by the reviewer, electrospray ionization can generate complex mass spectra, due to a number of reasons, which include adduct formation, multiple charge states, and in-source fragmentation which can complicate the mass spectra of heterogenous mixtures of glycopeptides, and make identification of each peak difficult by intact mass alone. To this end, we labeled unidentified peaks in the spectra.

• In Figure 5, I have a similar comment – what are the unannotated peaks? Also, since Fig 4b is MALDI of released glycans, would it not make sense to move 4b to figure 5?

Response: As noted by the Reviewer, we observed several unidentified peaks in MALDI-TOF/MS spectra of O-glycans released by POGase (Figure 5). We suspect that many of these unidentified peaks are causes by either the matrix clustering effect, which is common with MALDI techniques, and other non-glycan contaminants from the POGase enzyme and sample preparation. At this time, we purify POGase enzymes by HIS-Trap chromatography, and have not fully refined the purification process, or formulation. As your suggestion to move Fig. 4b to Fig. 5, we respect your point. But, Fig. 4 is mainly examining the de-glycosylated (glyco)peptides after POGase AS treatment, while Fig. 5 is to look for what glycans were released from the digestion, we think it is better to keep them separate in two figures.

• Extended Data 6 and 7 are very hard to read. Why are 6d/e so noisy? Is the lower signal due to the lower efficiency of glycan release?

Response: We thank the reviewer for these comments on the resolution of the tandem mass spectra in extended figure 6 and 7. We have updated both figures with high quality spectra, and new glycan fragment cartoons with high resolution. Regarding the noise in 6d and 6e, we believe the noise is caused by lower efficiency of POGase AS on Core 2, and 2,3SialylCore 2 glycan release. For all the glycopeptide reactions in the manuscript, we utilized the same amount of starting glycopeptide substrate and POGase AS enzyme to normalize the reactions.

• In Figure 6, what are the unannotated high and low mass peaks? Why are the high mass peaks in all three proteins?

Response: We thank the reviewer for these comments. We updated figure 6 to higher resolution figures, and the unidentified peaks in the spectra are now labeled by mass. Many of these unidentified peaks are present specifically in the middle panels of original 6a in which O-glycans are released by POGase AS from undenatured glycoprotein. We suspect that these are background peaks which are pronounced by low efficiency of O-glycan release from undenatured glycoprotein. These background peaks are gone when O-glycans are released by POGase from O-glycopeptides generated by proteinase K, most likely because the efficiency of O-glycan release is much better from short O-glycopeptide substrates, when compared to the intact protein. Additionally, other non-specific peaks are also present in figure 6a in which O-glycopeptides of Fetuin are treated with NEB O-glycosidase, and no sialylated O-glycans were released.

- A discussion on the three proteins and their expected glycan structures would be useful. Fig 6b makes it appear as if there is only one glycan structure on all of the proteins, but this would be unusual. Even in CHO-produced proteins, some di-sialylated core 1 structures would be expected.

  Response: We note the reviewer's important comment on the importance of showing the baseline O-glycan profile of all three glycoproteins for direct comparison of O-glycans release by POGase. To address the Reviewer's comment, we have reformatted figure 6 so that each panel now shows the baseline O-glycan profile of O-glycans release by β-elimination, prepared using our optimized one-pot glycomic method (Ortega-Rodriguez et al, *Cell Reports Methods*, 2024, DOI: 10.1016/j.crmeth.2024.100834). This method is standard practice in our group, in which short O-glycopeptides are first generated by proteinase k, and then O-glycans are released though a principle of β-elimination in the presence of NaOH during solid-phase permethylation. The resultant free O-glycans with free reducing ends are immediately methylated as described. Our method does not only efficiently release all O-glycans but also possesses undetectable peeling. This approach is standard practice in our group, which is why the O-glycans released by β- elimination were released in accordance with our method. As shown in figure 6c, d and e, Fetuin, has three major sialylated O-glycan species which include mono and di-sialyl core 1 (879 m/z, 1240 m/z respectively) and di-sialylCore 2 (1689 m/z). Abatacept and etanercept has two major sialylated O-glycan species mono and di-sialylcore 1 (879 m/z, 1240 m/z respectively). It's important to note that our optimized One-pot method results in unreduced O-glycans, and we chose to reduce O-glycans released by POGase, in order to differentiate between β-eliminated O-glycans that might contaminate the spectra if residual O-glycopeptides undergo permethylation, which will result in O-glycan release. Residual β-eliminated O-glycans would be identifiable by a mass difference of -16 m/z, consistent with the β-eliminated profile show in each spectra.

- What kind of excipients are present in the abatacept solution? Would it be possible to buffer exchange these contaminants out of solution and repeat the experiment?

Response: We thank the Reviewer's comment. We are not sure about the detailed excipients presented in the abatacept solution. As this work focuses on characterization for the previously unidentified enzyme, we will investigate the possible excipient(s) that may inhibits the POGase activity in a future study.

- Fig 6c. – please quantify the differences between beta-eliminated/POGase released glycans.

Response: We agree with the reviewer that quantifying the release of O-glycans between POGase and β- elimination is important. The abundance of each peak was determined between β-eliminated/POGase and NEB O-glycosidase in the original figure and a new panel in the revised figure (6b) was added to the manuscript. The data demonstrate that POGase As prefers cleavage of mono-sialylCore 1. Di-sialyl Core 1 and Core 2 O-glycans released by POGase AS accounted for less than 0.01% of the total O-glycan profile, compared to 18.3% (di-sialyl Core 1) and 1.2% (di-sialyl Core 2) which were observed in β- eliminated O-glycans, which release without bias. In contrast, NEB O-glycosidase did not release any sialylated O-glycans from fetuin.

• Figure 8 is difficult to read/decipher. Can the text be limited or removed for clarity?

Response: We thank for the Reviewer's comment. We have revised the Figure 8, in which the texts were removed, and consensus binding glycan-motifs are simplified and summarized in our revised manuscript.

Minor comments

• "Despite the formation"… "lack a common core" – this seems contradictory. It makes sense that if there are 20 GalNAcTs that the downstream processing will also be similarly convoluted. I would re-word this sentence.

Response: We thank the Reviewer's critics. We re-worded the sentence in our revised manuscript.

• The authors mention a commercially available O-glycanase early in the paper but cite an academic laboratory. Later, they mention NEB as the manufacturer, but it would be helpful to mention at the first reference of the enzyme.

Response: We thank the Reviewer's comment and suggestion. We added the commercial source of the EngEF at the first reference of the enzyme in our revised manuscript.

• A brief, 1-2 sentence introduction to LIFT-MS would be useful.

Response: We understand the reviewer's comment on defining LIFT-MS in the manuscript. LIFT is a proprietary technique from Bruker molecular ions are initially accelerated at lower energy, then collide with inert gas in the collision cell that is then lifted to high potential. The MALDI-TOF-MS in our lab has the capability to switch between collision gas to generate fragmentation products, or laser induced dissociation (LID) for fragmentation (PMID:12830354). Fragments generated by LID can also undergo acceleration at lower energy and lifted to high potential through the collision cell. The manuscript has been updated to define LIFT-MS, and LIFT was changed to tandem mass spectra across the manuscript to make it easier for readers to understand.

• Line 337 – the authors say "POGases" but really only seem to fully characterize AS. Might be useful to clarify this here.

Response: We understand the Reviewer's comment. We only focused on characterization of POGase AS because characterization of all 5 POGase orthologs identified in this work would take much larger effort and time, which we are currently conducting now, and those orthologs will be compared and published in our follow up studies. The POGase AS was chosen in this work is because this enzyme relatively easier to express at required amount of protein for characterization studies.

• Why was POGase TB chosen for the N vs C terminal studies?

Response: We thank the Reviewer's comment. The reasons for POGase TB chosen for the N- vs C-terminal studies were that: 1) POGase TB (#22) was the first one identified with significant activity towards sialylCore 1 O-glycans in the first 30 putative O-glycanses for screening; 2) expression of their domains was carried out earlier. We will investigate and publish the domain functions of other POGase orthologues in the follow up studies.

• Throughout the document, the authors refer to the enzymes as "novel", but this is slightly odd language for enzymes that have existed throughout evolution. Perhaps "previously uncharacterized" is more accurate?

Response: We thank the Reviewer's critics. We change the "novel" to "previously uncharacterized" throughout the manuscript.

• The authors mention "complex O-glycans" but "complex" is often reserved for N-glycan structures. I believe the authors mean sialylated O-glycans, so this wording should be altered.

Response: We thank the Reviewer's comment. We change the "complex O-glycans" to "sialylated O-glycans" throughout the manuscript.

Reviewer #4 (Remarks to the Author):

In this manuscript, the authors identify a family of enzymes, which they term peptide:O-glycosidases (POGase), that are capable of cleaving substrates containing glycans with an initiating GalNAc residue. Some enzymes were found to cleave substrates with different core structures (1-3), including those modified with sialic acid. The enzymes appear to have broader glycan tolerance than related glycoside hydrolases (GHs) characterized to date. Phylogenetic analysis and modeling were performed to help rationalize this observation. Finally, one enzyme was found to bind specific structures in the glycan-binding array.

A universal O-glycan hydrolase is not known, as most GHs do not act broadly on all O-glycans. POGases with broader substrate tolerance could help fill that void and be of significant use to the field. That being said, I believe the authors need additional/improved studies to better characterize these enzymes and demonstrate such utility/impact before publication is warranted. As my background is primarily with modeling, my comments will largely focus on that portion of the study.

Major Critiques:

1. While the authors emphasize the importance of mucin-type glycosylation, the protein-based substrates (fetuin and etanercept/abatecept) are sparsely glycosylated and/or have a very small mucin-domain. To better understand the substrate tolerance/preference of these enzymes (which the authors admit may have some steric limitations), the authors should characterize activity on a recombinant mucin or larger mucin-domain-containing protein.

Response: We thank the Reviewer's comment and vision on better and deeper understanding of enzymatic mechanism(s) of the POGases. We acknowledge the limitations that the POGase AS has as it cannot efficiently release all sialylated O-glycans, especially for di-sialylated species from glycoproteins. As the Reviewer pointed out, further characterizing activity on a recombinant mucin or larger mucin-domain-containing protein, as well as cell extracts is essential for better understanding of the enzyme which would lead to identify and generate a universal POGase. But we realize that would be a project itself, rather than simple experiments. Therefore, while we very much respect the Reviewer's comment and suggestion, we will be reporting the results in our follow up manuscript(s) as we are conducting that study in one of ongoing projects.

2. Furthermore, the authors should benchmark better against GHs (i.e. the used NEB O-glycosidase) on the synthetic glycopeptides and glycoproteins (beyond just fetuin, which does not contain a mucin-domain). Without doing this, it is difficult to appreciate the utility of POGases as a tool for glycobiology… which is pivotal to demonstrating the advantage of POGases over currently used GHs.

Response: The Reviewer's comment-2 is continuous of Comment-1. We agree with the Reviewer's comment on benchmarking POGase AS with NEB O-glycosidase in order to demonstrate the advantage of POGase. Importantly, we have demonstrated that the POGase has the advantage over currently used GH101(Figure 5 and Figure 6) using defined glycopeptides as well as glycoproteins, fetuin and 2 biotherapeutics. This work is focusing on identifying and characterizing the previously unidentified enzyme, as opposed to developing biotechnology for O-glycomics which is currently ongoing in the follow up study. We acknowledge the POGase AS is not a perfect tool yet, but this work is a great step forward in developing/generating a true universal POGase with efficient releases of all sialylated O-glycans in future which will promote the functional O-glycomics. Similar to our response to this Reviewer's comment-1, we respect the Reviewer's comment, we are conducting the relevant studies will be reporting soon.

Overall, this work is not about biotechnology or tool development. As we discussed, the POGase AS is not a perfect tool yet, however, this work is a step forward to potentially developing a feasible biotechnology/tool for O-glycomics. We believe the audiences will appreciate the study, and more importantly, it will also impact numerous scientific disciplines including glycobiology, biotechnology, microbiology, immunology, and medicine. We respect the Reviewer's comment, we are conducting the study and will be reporting in a different manuscript soon.

3. The glycan array data is overinterpreted: there's no direct data demonstrating cellular adhesion. Accessory domains in some O-glycoproteases/mucinases have been suggested (and even observed in Glycobiology 2021, 31, 385) to be involved in secondary recognition of larger mucin substrates. That the N-terminal domain and the C-terminal domain constructs lack enzymatic activity (Extended Figure 10) suggests that the overall fold or cooperativity between the two domains has been lost. Regardless, it's difficult to ascertain if these or some other mechanism is at play without doing larger structural study.

Response: Thank you for the comment. We agree with your critical point that the full-length of POGase acts at enzymatic activity toward sialylated O-glycans by cooperatively folding or coordinating the N- and C-terminal domains. To get direct evidence to support that the POGase is a potential adhesin, we conducted the cell-binding study. As shown in the new Fig. 9 in our revised manuscript, we found that both full-length POGase and POGase-CD can bind to the human cell line HEK293 cells, and enhanced binding weas observed after desilaylation of cells. More importantly, both full-length POGase and POGase-CD only had moderate binding to the HEK293 cells-CKO cells which only express truncated O-glycans, the Tn antigens. The POGase-ND did not show any binding to either cell lines. As HEK293 cells do not express type-I

chains on N-glycans, our cell-binding data demonstrated that POGase does bind to the HEK293 cells in an O-glycan dependent manner.

Modeling Critiques:

The modeling work follows standard protocols to generate model structures (AlphaFold2) and perform docking (AutoDock). The generated enzyme structure appears to have undergone quality control. The docking, by contrast, has not gone through sufficient quality control/analysis.

The current docked poses (Figure 7 & Extended Data Figure 9) have some questionable results, including several instances where (1) the anomeric carbon of the GalNAc residue is distant from the DDE catalytic triad and (2) there are high-energy cis amide bonds in the NAc moiety and ring flips with some sugars. Additionally, the GalNAc and Gal moieties are oriented with their nonpolar faces away from the WW motif (preventing CH-pi stacking), which is unexpected given (a) what is known about GalNAc recognition (Acc. Chem. Res. 2023, 56, 548) and – more importantly – (b) poses of glycans observed in X-ray structures of other GH101-containing enzymes (J. Biol. Chem. 2015, 290, P25657). As such, I doubt that any of the docked poses reflect the actual binding pose of glycans.

The authors should redo the docking studies, ideally using an enzyme-T antigen cocrystal structure from the aforementioned JBC article as a guide to initially place the T-antigen into active site of the modeled enzyme. This can be docked using AutoDock. From there, the other core structures can be easily generated: modifying sugars of the docked T-antigen and/or “grafting” other crystallographic sugars (which can be searched using http://glycanstructure.org/fragment-db) onto the T-antigen core and generating a small library in which the new sugars are allowed to adopt different conformations. Each conformer library can then be docked using AutoDock as before and you can get improved results. This process will help ensure that docking begins with low energy ligand conformers placed in approximately the “right” place within the pocket. Yes, it introduces bias into the system, but it is at least grounded in prior results and allows for more meaningful data interpretation.

As it stands, the docking doesn’t add much to the story of the paper. I would recommend the authors (1) compare their docked structures to the JBC crystal structures to better understand why the POGases permit larger and more diverse glycans than GHs and (2) possibly dock glycans into modeled structures of POGases that are less active or more selective to better understand these differences, as the authors suggest that these enzymes could be engineered for better results.

Finally – partially because the coloring between Figure 7a and 7b are inconsistent – it remains unclear how the conserved, ligand-binding residues of the F5/8 type C domains (which can probably be determined using UniProt) spatially relate to the active site. Again, it is very well possible that the noncatalytic domains participate cooperatively in binding larger, densely glycosylated substrates like mucins rather than engaging in cellular adhesion, as the authors have implied.

Response*:* We thank this Reviewer for the critics and suggestions. We have re-done the docking as suggested. Specifically,

1) Considering the intramolecular energy of the disaccharide glycan bonds does not change the result suggesting that while the glycans are strained in their confirmation, the intermolecular contacts between the glycan and enzyme active site can compensate for some of the nonideal torsion angels, as would be expected in the context of induced fit.
2) Considering the intramolecular energy of the sialylated glycan bonds does change the docking result. After considering the intramolecular energy of the sialylated glycan the bound-pose more

closely resembles the bound-pose of non-sialylated glycans. This again suggests coordination between the intermolecular active-site contacts of flexibility of the glycan substrate.

3) The anomeric carbon in the results isn't any further from the catalytic triad than the experimental structure that the reviewer referenced.
4) Interaction through π-stacking is not considered in the molecular force field of AutoDock Vina or the alternative VinaCarb. However, because the enzyme is in its apo form, like one of the conformations of the GH101 domain in 5a56, the tryptophans that act as an active site lid are not expected to contribute to the docked pose. MD trajectories long enough to allow for the apo to substrate bound conformational shift of the enzyme were outside the scope of this study.
5) An analysis was added to hypothesize why the structure of POGase AS allows for coordination of sialylated glycans, while others don't (Changes in motif 1 and changes to hydrophobic core interactions)
6) Other POGase structures have slightly different docking results, focus of the docking for this study was POGase AS, other model POGases will be used for future studies.

We have incorporated these into the corresponding Results and Discussion, as well as the Methods Sections in the revised manuscript. As discussed in the manuscript, the predicted structures through modeling will be either confirmed or corrected through either cryo-EM or Crystallography experiments in future studies as much more needs to be studied and learned.

RESPONSES TO REVIEWER COMMENTS

Nature Communications: Manuscript NCOMMS-24-20598-T

Reviewer #1 (Remarks to the Author):

This work highlights the utility of a novel O-glycanase for O-glycomics and function as adhesins. The researchers characterized the binding activity of the novel POGase as well as the rate of deglycosylation. Initially 5 POgases were characterized and identified, however one POGase (POGase AS) was the standout enzyme in the group. This POgase was the focus of majority of the paper. One of the stand-out aspects of this enzyme is the ability to remove sialylated core1 and core 2 O-glycans, which most currently available commerical O-glycanases can not do. I believe the identification and characterization of this novel POGase will be extremely beneficial to the field of glycobiology and glycochemistry. However the paper does require some editing before it should be accepted for publication.

1. The in-text citations appear within the period, when they should be outside of the period when in superscript form. This needs to be fixed throughout the article.

Response**:** We thank the Reviewer's comment. We carefully looked at the published articles, the citations are within the period.

2. A general proof reading is needed. There are multiple cases where a period, space, or comma are missing. There are also multiple points throughout the paper where one sentence will switch from singular to plural tense (The last sentence of the introduction is an example of this. It starts mentioning multiple POGases, then says "has" instead of "have", and then sentence ends with no period).

Response**:** We thank the Reviewer's critical reading. We have made corrections of those grammatical errors and typos in this revised manuscript.

3. multiple times it is mentioned how the POGases can remove glycans from "glycopeptides, glycoproteins and therapeutic glycoproteins" I understand the clarification between glycopeptides and glycoproteins, however why is it necessary to clarify therapeutic glycoproteins vs non? What structurally would make this necessary to clarify?

Response**:** We thank the Reviewer's comment. This is an emphasis of our research as we work at the US FDA in regulating the therapeutic glycoproteins. We changed the sentence to "glycopeptides, glycoproteins, including therapeutic glycoproteins" in the revised manuscript.

4. When discussing the deglycosylation abilities of the POGases for glycomics analysis purposes, the term "native peptide" is used to describe the non-glycosylated peptide. This is very confusing. Since these proteins are glycosylated in nature, and in many cases the native form is glycosylated, this is not the best term choice in my opinion. Using "deglycosylated peptide", "non-glycosylated peptide", "unmodified peptide: would all be better options.

Response**:** We thank the Reviewer's comment. We agree with the Reviewer. We changed the "native peptide" to "unmodified peptide".

5. When discussing the Muc5Ac and CD24 peptides, multiple times the term "a2,3sialylcore 2" is used, however there are multiple points where the authors discuss using MSMS to confirm the glycan structures. Do you know the sialic acid is 2,3 linked because the peptides were purchased and characterized or because MSn experiments were conducted? If they were not conducted, how do you know the sialic acid is 2,3 linked?

Response: We understand the Reviewer's comment. α2,3sialylcore 2-glycopeptides were made synthesized in-house using step-by-step recombinant glycan extension with recombinant glycosylatransferases and donor substrates. Specifically we used ST3Gal1 and CMP-sialic acid to generate α2,3sialyl Core 1, and Core 2 from synthetic Core 1, Core 2 MUC5AC-glycopeptides to make the α2,3sialylation. The purpose of the MS/MS experiments were to demonstrate that POGase AS could release a variety of intact O-glycans, based on distinct permethylated glycan fragments observed in the tandem mass spectra (Extended Figure 6).

6. The terms LIFT-MS and MALDI-TOF-MS need to be defined

Response: We understand the reviewer's comment on defining LIFT-MS and MALDI-TOF-MS in the manuscript. MALDI-TOF-MS is the abbreviation for matrix assisted laser desorption ionization time of flight mass spectrometry, is a class of mass spectrometry which is commonly used in the field for analysis of permethylated glycans. LIFT is a proprietary technique from Bruker molecular ions are initially accelerated at lower energy, then collide with inert gas in the collision cell that is then lifted to high potential. The MALDI-TOF-MS in our lab has the capability to switch between collision gas to generate fragmentation products, or laser induced dissociation (LID) for fragmentation (PMID:12830354). Fragments generated by LID can also undergo acceleration at lower energy and lifted to high potential through the collision cell. The manuscript has been updated to define MALDI-TOF-MS, and LIFT-MS was changed to tandem mass spectra across the manuscript.

7. When comparing enzymatically released O-glycans from beta eliminated, the beta eliminated glycans were left as free ends, and the POGase glycans were reduced. This is said to be to help distinguish them, but why not reduce both? Are you releasing them from the same aliquot of protein...? Why do you need to distinguish them when they are processed separately? To me, it makes the most sense to have both in reduced form, as it will ensure everything besides the release mechanism are the same. It will also make sure ionization efficiency is not effected.

Response: We note the reviewer's important comment on reducing O-glycans released by β-elimination. The β-eliminated O-glycans were prepared using our optimized one pot glycomic method (Ortega-Rodriguez et al, *Cell Reports Methods*, 2024, DOI: 10.1016/j.crmeth.2024.100834) in which short O-glycopeptides are first generated by proteinase K, and then O-glycans are released though a principle of β-elimination in the presence of NaOH during solid-phase permethylation. The resultant free O-glycans with free reducing ends are immediately methylated as described. Our method does not only efficiently release all O-glycans but also possesses undetectable peeling. This approach is standard practice in our group, which is why the O-glycans released by β-elimination were released in accordance with our method.

8. Did you attempt any deglycosylation studies on complex samples? Though this enzyme will be useful for O-glycomics of simple purified proteins, I find it difficult to find the validity in this helping with complex samples. Majority of researchers will have a greater need for de-o-glycosylating complex samples.

Response: We understand the Reviewer's point. We have tried the O-glycan release from more complex samples such as cell extracts. At this time, we have not worked out an optimum condition to release, recover and analyze the O-glycans from digestion of cell extracts using mass spectrometry, probably due to the complexity of samples in nature, and importantly the limited accessibility of POGase to mucin-domains of glycoproteins from cell extracts which is the common obstacle for O-glycomics. We continue investigating this. We have included this in our revised manuscript. Nevertheless, besides the limitation, our work has the following merits:

1) This is the first identification of naturally occurring enzyme with significant activity to cleave the most common O-glycans, α2,3sialylCore 1, and residual activity to cleave more complex sialylated O-glycans, such as di-sialylCore 1 and Core 2.
2) We have shown that POGase AS can release intact sialylCore 1 and others from glycoproteins, fetuin and 2 therapeutic glycoproteins.
3) This work has demonstrated that the POGase has potential adhesin activity in addition to its enzymatic activity.
4) This work has a significant implication to the microbiology, for example, how the POGases enable bacteria to colonize, and its potential roles in attachment/invasion of pathogens or opportunist pathogens.

Overall, this work is not about biotechnology or tool development. As we discussed, the POGase AS is not a perfect tool yet, however, this work is a step forward to potentially developing a feasible biotechnology/tool for O-glycomics. We believe the audiences will appreciate the study, and more importantly, it will also impact numerous scientific disciplines including glycobiology, biotechnology, microbiology, immunology, and medicine. We respect the Reviewer's comment, we are conducting the study and will be reporting in a different manuscript soon.

9. You have a figure showing the effects of the POgase when the protein has been denatured vs not, which in my opinion is worth noting but I am not sure how novel that is to include within a main text figure. Instead it might be a better option to compare the glycopeptide to the glycoprotein. I.e. for fetuin compare the results of the POGase on intact fetuin vs fetuin peptides.

Response: We understand the Reviewer's comment. Our figures have been reformatted to focus only on O-glycans released by POGase from denatured proteins, and O-glycopeptides generated by Proteinase K-. Reformatted figures to focus only on the protein vs peptides (new figure 6). Additionally, we have added profiles of O-glycans released by β-elimination (using our optimized one pot glycomic method) above the spectra of O-glycans released by POGase from glycopeptides and denatured fetuin, abatacept, and etanercept. The purpose of this change is to emphasize that POGase is capable of releasing most of the O-glycans, but the enzyme prefers to cleave 2,3sialylCore 1 O-glycans over other more sialylated O-glycan species.

Reviewer #2 (Remarks to the Author):

Zhou and colleagues have successfully screened a wide variety of putative POGases and found one in particular that was relatively fast and had broad-specificity. The overall presentation of the work is good and easy to interpret (I have included a few points on figures), the order of the different aspects is also logical. The writing is largely clear and provides easy understanding for the reader. I have no major issues, but a few queries/comments.

• How did the authors pick these enzymes and why? It would be interesting to know where the 30 you screened plus all the characterised ones sit in a phylogenetic tree of all GH101s. I think this would be more insightful than Figure 9.

Response: We understand the Reviewer's comment. Initially, we selected the candidates for testing their potential activity to cleave sialylCore 1-(4-MU) based on these thoughts: 1) predicted to be an endo-α-N-acetyl-β-D-galactosaminidase from BRENDA database; 2) Previously uncharacterized candidates from different bacteria; 3) large proteins, >1,500aa long. Now we know all 30 enzymes plus characterized ones

belong to GH101, a bigger phylogenetic tree, but the POGases with activity to cleave sialylated O-glycans grouped in a monophyletic clade contained within the GH101 phylogenetic tree, specifically within the group of GH101 enzymes expressed in *Actinomycetes* in Fig. 10 in the revised manuscript.

• Line 99 – is that the activity of the GH16 enzymes?

***Response*:** Yes, the enzyme identified in the work was a member of GH16. It is not an O-glycanase, is actual endo-β-N-acetyl-D-glucosaminidase. The term, O-glycanase in that paper confuses readers.

• Figure 7 – annotate the different colours in a and the sugars in b. What are the salmon-coloured residues. Personally, I don't like the black background, but that's being picky.

Response: We agree with the Reviewer's suggestion, and have changed colors to match in each modeling data with white background in our revised manuscript.

• Line 396 – what are blood coagulation factors, why is it relevant?

Response: We understand the Reviewer's comment. The 5C/F Domain was also found the blood coagulation factors, it is irrelevant to this work. We deleted it from this revised manuscript.

• I'm not clear on your use of the term 'O-glycanase' throughout – are you referring to GH101 enzymes? I think, from the way you write, you want the POGases to be a class within GH101 enzymes? If you take the sequences of just the catalytic domains GH101 enzymes, do the POGases group together on a phylogenetic tree?

Response: We note the Reviewer's comment. We use the term 'O-glycanase' throughout to refer to GH101 enzymes. The newly identified POGases are a class within GH101 enzymes, and grouped in a monophyletic clade on the GH101 phylogenetic tree (Fig. 10). Based on the published and our current ongoing studies, it appears that the GH101 family include various sub-families of enzymes, with each sub-family processing distinct enzymatic/substrate specificity. While POGases are only expressed in one phylum, the GH101 family seems to express a wide diversity of subclasses, but that information is outside the scope of this current study. We are now investigating and will continue reporting them.

• Figure 8 – N-glycans have beta-1,4-galactose, not beta-1,3.

Response: We understand the Reviewer's comment. In N-glycans, typically beta1,4-Galactose is attached to N-Acetylglucosamine, known as Type-II chain. We carefully took a look at the GlycanArray results, and found that both full-length and C-terminal domain of POGase bind to the bivalent Galbeta1,3GlcNAc-, the Type-I chains on N-glycans, but not Type-II chains. The GlycanArray used in this study is composed of known glycan structures synthesized chemically and enzymatically, including Type-I chains on N- and O-glycans, and glycolipids (PMID: 21488041).

• Figure 9 – I think this figure need work to make it useful. I'm not sure what the colours are.

Response: We apologize for not including the color codes. We added clarifying text in the article and a key for the colors in the revised figure.

• Line 492 – I think this starting sentence contradicts what the rest of the paragraph says. Do you mean that O-glycanases have motifs that are also in other enzymes? I'm not sure.

Response: We thank the Reviewer's critics. We revised the starting sentence as " POGases have both unique and shared sequence motifs compared to known O-glycanases."

• Line 518-9 – a bit of repetition here. I'm not sure how there will be the development of therapeutic O-glycoproteins. In this paragraph, how would a GH101 be able to accommodate much longer O-glycans? I had a quick look at the Strep pneumo structure with only the catalytic domain and there's nothing stopping a longer O-glycan being accommodated in theory. How does your model compare to this GH101?

Response: We are sorry for the confusion caused by lack of clarity in the manuscript. We address this by discussing the flexibility of the POGase specific motifs as well as the inclusion of the hydrophobic core interactions compared to the known O-glycanses. We also hypothesize that charge states of the motifs might contribute to the increase in substrate pools for POGases. To summarize, as discussed in the main text, the flexibility of the motifs, the change in hydrophobic core interactions, and the change in charge states are hypothesized to play a role in increased substrate pool, but more experiments need to be done to verify in future studies. As for the development of therapeutic O-glycoproteins, we meant that a universal POGase can make it possible for the characterization and control of O-glycosylation of therapeutic O-glycoproteins, thus facilitate the drug development.

• Paragraph line 536 – I think the binding data is good, but I think it's a step too far to talk about disease if these enzymes haven't been linked. This paragraph is a bit speculative.

Response: We respect the Reviewer's comment. To further prove the adhesin activity of POGase, we performed cell-binding experiments to show that the full length POGase, and its C-terminal domain, but not the N-terminal domain of POGase TB can specifically bind to a human cell line, HEK293 cells mainly after de-sialylation. Regarding its role in human diseases, one study showed that the EngCP, a known O-glycanase from *Clostridium perfringens* is a virulence factor involved in gangrene infections. Based on this finding, we speculate the pathogenic role of POGases in human and animal diseases, raise the alarm to the relevant field to pay attention and investigate the potential role of these POGases in diseases.

• Paragraph 571 – where is this data? I think you need to show a tree of GH101s if you are going to say this

***Response**:* We thank the Reviewer's comment. We clarified to demonstrate that the tree being shown is the GH101s isolated from the database tree we generated (Fig. 10) in the revised manuscript.

Reviewer #3 (Remarks to the Author):

In the manuscript by Zhou et al., the authors characterize a previously unstudied family of glycan hydrolases they term "POGases". These enzymes are capable of releasing O-glycan structures that are sialylated and can operate on synthetic glycopeptides as well as therapeutically relevant glycoproteins. The authors perform a thorough investigation and MS-based analysis using POGase AS. They also perform phylogenetic and structural studies to understand why these enzymes are capable of releasing sialylated O-glycans while other known GH101-members are not. Since a universal O-glycanase is not known and highly valuable in glycobiology, this is a major contribution to the field. Overall, the authors present a very thorough, detailed, and well-written manuscript that I largely support. That said, the paper does have a few areas for improvement.

Major concerns

• The figure quality throughout the manuscript is very low. Many of the spectra/fonts/numbers are completely illegible and thus difficult to interpret. Several spectra have unannotated peaks. Many images are low resolution.

Response: We thank the Reviewer's comment. The low quality of the figures was partly due to the embedding of figures into the text in the pre-submission to *Nature*, which was then directly transferred to *Nature Communications* for your review. We have revised our figures in high resolution, and labeled the unidentified peaks in MS spectra.

• For Figure 4C-E, the enzymatic release is incomplete. Did the authors attempt to change the time of reaction, concentration of enzymes, etc. to optimize full release of the glycans? If not, I would recommend attempting this to maximize utility of the enzyme.

Response: We thank the Reviewer for this important note. We attempted several approaches to get full optimized release, including using excess enzyme, different buffers, different incubation times, and we did not observe complete release. Most likely a protein stability issue which that could we plan to be address ed with precise, in-depth formulation studies in a future paper. It could also be due to the significant inhibition by the product. We are now investigating these and get an optimum condition to efficient release of sialylated O-glycans.

- Related to the same figure, can the authors quantify these peaks/efficiency of glycan release?

  Response: We agree with the Reviewer, that it is important to quantity the peaks observed in both the controls, and digested samples, in order to understand the efficiency of glycan release with POGase AS. The extracted ion chromatograms (XICs) of each peak were extracted using a mass tolerance of 0.0500 dalton, to facilitate high accuracy mass filtering. The XIC peak area exported % peak area of each substrate peak, and product peak was summarized in a Fig. 6b, and Extended Data figure 6 to provide with %Peak area. The data clearly shows the difference in product peaks after POGase treatment (relative abundance based on extracted ion chromatograms (XIC) of each product peak, underscoring that POGase can fully release 2,3sialylated Core 1, and Core 2 O-glycans, with preference to 2,3sialylCore 1.

- Several other species are in the spectra, what are these? If they cannot be identified by intact mass, which is common, perhaps label them as such?

  Response: As noted by the reviewer, electrospray ionization can generate complex mass spectra, due to a number of reasons, which include adduct formation, multiple charge states, and in-source fragmentation which can complicate the mass spectra of heterogenous mixtures of glycopeptides, and make identification of each peak difficult by intact mass alone. To this end, we labeled unidentified peaks in the spectra.

• In Figure 5, I have a similar comment – what are the unannotated peaks? Also, since Fig 4b is MALDI of released glycans, would it not make sense to move 4b to figure 5?

Response: As noted by the Reviewer, we observed several unidentified peaks in MALDI-TOF/MS spectra of O-glycans released by POGase (Figure 5). We suspect that many of these unidentified peaks are causes by either the matrix clustering effect, which is common with MALDI techniques, and other non-glycan contaminants from the POGase enzyme and sample preparation. At this time, we purify POGase enzymes by HIS-Trap chromatography, and have not fully refined the purification process, or formulation. As your suggestion to move Fig. 4b to Fig. 5, we respect your point. But, Fig. 4 is mainly examining the de-glycosylated (glyco)peptides after POGase AS treatment, while Fig. 5 is to look for what glycans were released from the digestion, we think it is better to keep them separate in two figures.

• Extended Data 6 and 7 are very hard to read. Why are 6d/e so noisy? Is the lower signal due to the lower efficiency of glycan release?

Response: We thank the reviewer for these comments on the resolution of the tandem mass spectra in extended figure 6 and 7. We have updated both figures with high quality spectra, and new glycan fragment cartoons with high resolution. Regarding the noise in 6d and 6e, we believe the noise is caused by lower efficiency of POGase AS on Core 2, and 2,3SialylCore 2 glycan release. For all the glycopeptide reactions in the manuscript, we utilized the same amount of starting glycopeptide substrate and POGase AS enzyme to normalize the reactions.

• In Figure 6, what are the unannotated high and low mass peaks? Why are the high mass peaks in all three proteins?

Response: We thank the reviewer for these comments. We updated figure 6 to higher resolution figures, and the unidentified peaks in the spectra are now labeled by mass. Many of these unidentified peaks are present specifically in the middle panels of original 6a in which O-glycans are released by POGase AS from undenatured glycoprotein. We suspect that these are background peaks which are pronounced by low efficiency of O-glycan release from undenatured glycoprotein. These background peaks are gone when O-glycans are released by POGase from O-glycopeptides generated by proteinase K, most likely because the efficiency of O-glycan release is much better from short O-glycopeptide substrates, when compared to the intact protein. Additionally, other non-specific peaks are also present in figure 6a in which O-glycopeptides of Fetuin are treated with NEB O-glycosidase, and no sialylated O-glycans were released.

- A discussion on the three proteins and their expected glycan structures would be useful. Fig 6b makes it appear as if there is only one glycan structure on all of the proteins, but this would be unusual. Even in CHO-produced proteins, some di-sialylated core 1 structures would be expected.

  Response: We note the reviewer's important comment on the importance of showing the baseline O-glycan profile of all three glycoproteins for direct comparison of O-glycans release by POGase. To address the Reviewer's comment, we have reformatted figure 6 so that each panel now shows the baseline O-glycan profile of O-glycans release by β-elimination, prepared using our optimized one-pot glycomic method (Ortega-Rodriguez et al, *Cell Reports Methods*, 2024, DOI: 10.1016/j.crmeth.2024.100834). This method is standard practice in our group, in which short O-glycopeptides are first generated by proteinase k, and then O-glycans are released though a principle of β-elimination in the presence of NaOH during solid-phase permethylation. The resultant free O-glycans with free reducing ends are immediately methylated as described. Our method does not only efficiently release all O-glycans but also possesses undetectable peeling. This approach is standard practice in our group, which is why the O-glycans released by β- elimination were released in accordance with our method. As shown in figure 6c, d and e, Fetuin, has three major sialylated O-glycan species which include mono and di-sialyl core 1 (879 m/z, 1240 m/z respectively) and di-sialylCore 2 (1689 m/z). Abatacept and etanercept has two major sialylated O-glycan species mono and di-sialylcore 1 (879 m/z, 1240 m/z respectively). It's important to note that our optimized One-pot method results in unreduced O-glycans, and we chose to reduce O-glycans released by POGase, in order to differentiate between β-eliminated O-glycans that might contaminate the spectra if residual O-glycopeptides undergo permethylation, which will result in O-glycan release. Residual β-eliminated O-glycans would be identifiable by a mass difference of -16 m/z, consistent with the β-eliminated profile show in each spectra.

- What kind of excipients are present in the abatacept solution? Would it be possible to buffer exchange these contaminants out of solution and repeat the experiment?

Response: We thank the Reviewer's comment. We are not sure about the detailed excipients presented in the abatacept solution. As this work focuses on characterization for the previously unidentified enzyme, we will investigate the possible excipient(s) that may inhibits the POGase activity in a future study.

- Fig 6c. – please quantify the differences between beta-eliminated/POGase released glycans.

Response: We agree with the reviewer that quantifying the release of O-glycans between POGase and β- elimination is important. The abundance of each peak was determined between β-eliminated/POGase and NEB O-glycosidase in the original figure and a new panel in the revised figure (6b) was added to the manuscript. The data demonstrate that POGase As prefers cleavage of mono-sialylCore 1. Di-sialyl Core 1 and Core 2 O-glycans released by POGase AS accounted for less than 0.01% of the total O-glycan profile, compared to 18.3% (di-sialyl Core 1) and 1.2% (di-sialyl Core 2) which were observed in β- eliminated O-glycans, which release without bias. In contrast, NEB O-glycosidase did not release any sialylated O-glycans from fetuin.

• Figure 8 is difficult to read/decipher. Can the text be limited or removed for clarity?

Response: We thank for the Reviewer's comment. We have revised the Figure 8, in which the texts were removed, and consensus binding glycan-motifs are simplified and summarized in our revised manuscript.

Minor comments

• "Despite the formation"… "lack a common core" – this seems contradictory. It makes sense that if there are 20 GalNAcTs that the downstream processing will also be similarly convoluted. I would re-word this sentence.

Response: We thank the Reviewer's critics. We re-worded the sentence in our revised manuscript.

• The authors mention a commercially available O-glycanase early in the paper but cite an academic laboratory. Later, they mention NEB as the manufacturer, but it would be helpful to mention at the first reference of the enzyme.

Response: We thank the Reviewer's comment and suggestion. We added the commercial source of the EngEF at the first reference of the enzyme in our revised manuscript.

• A brief, 1-2 sentence introduction to LIFT-MS would be useful.

Response: We understand the reviewer's comment on defining LIFT-MS in the manuscript. LIFT is a proprietary technique from Bruker molecular ions are initially accelerated at lower energy, then collide with inert gas in the collision cell that is then lifted to high potential. The MALDI-TOF-MS in our lab has the capability to switch between collision gas to generate fragmentation products, or laser induced dissociation (LID) for fragmentation (PMID:12830354). Fragments generated by LID can also undergo acceleration at lower energy and lifted to high potential through the collision cell. The manuscript has been updated to define LIFT-MS, and LIFT was changed to tandem mass spectra across the manuscript to make it easier for readers to understand.

• Line 337 – the authors say "POGases" but really only seem to fully characterize AS. Might be useful to clarify this here.

Response: We understand the Reviewer's comment. We only focused on characterization of POGase AS because characterization of all 5 POGase orthologs identified in this work would take much larger effort and time, which we are currently conducting now, and those orthologs will be compared and published in our follow up studies. The POGase AS was chosen in this work is because this enzyme relatively easier to express at required amount of protein for characterization studies.

• Why was POGase TB chosen for the N vs C terminal studies?

Response: We thank the Reviewer's comment. The reasons for POGase TB chosen for the N- vs C-terminal studies were that: 1) POGase TB (#22) was the first one identified with significant activity towards sialylCore 1 O-glycans in the first 30 putative O-glycanses for screening; 2) expression of their domains was carried out earlier. We will investigate and publish the domain functions of other POGase orthologues in the follow up studies.

• Throughout the document, the authors refer to the enzymes as "novel", but this is slightly odd language for enzymes that have existed throughout evolution. Perhaps "previously uncharacterized" is more accurate?

Response: We thank the Reviewer's critics. We change the "novel" to "previously uncharacterized" throughout the manuscript.

• The authors mention "complex O-glycans" but "complex" is often reserved for N-glycan structures. I believe the authors mean sialylated O-glycans, so this wording should be altered.

Response: We thank the Reviewer's comment. We change the "complex O-glycans" to "sialylated O-glycans" throughout the manuscript.

Reviewer #4 (Remarks to the Author):

In this manuscript, the authors identify a family of enzymes, which they term peptide:O-glycosidases (POGase), that are capable of cleaving substrates containing glycans with an initiating GalNAc residue. Some enzymes were found to cleave substrates with different core structures (1-3), including those modified with sialic acid. The enzymes appear to have broader glycan tolerance than related glycoside hydrolases (GHs) characterized to date. Phylogenetic analysis and modeling were performed to help rationalize this observation. Finally, one enzyme was found to bind specific structures in the glycan-binding array.

A universal O-glycan hydrolase is not known, as most GHs do not act broadly on all O-glycans. POGases with broader substrate tolerance could help fill that void and be of significant use to the field. That being said, I believe the authors need additional/improved studies to better characterize these enzymes and demonstrate such utility/impact before publication is warranted. As my background is primarily with modeling, my comments will largely focus on that portion of the study.

Major Critiques:

1. While the authors emphasize the importance of mucin-type glycosylation, the protein-based substrates (fetuin and etanercept/abatecept) are sparsely glycosylated and/or have a very small mucin-domain. To better understand the substrate tolerance/preference of these enzymes (which the authors admit may have some steric limitations), the authors should characterize activity on a recombinant mucin or larger mucin-domain-containing protein.

Response: We thank the Reviewer's comment and vision on better and deeper understanding of enzymatic mechanism(s) of the POGases. We acknowledge the limitations that the POGase AS has as it cannot efficiently release all sialylated O-glycans, especially for di-sialylated species from glycoproteins. As the Reviewer pointed out, further characterizing activity on a recombinant mucin or larger mucin-domain-containing protein, as well as cell extracts is essential for better understanding of the enzyme which would lead to identify and generate a universal POGase. But we realize that would be a project itself, rather than simple experiments. Therefore, while we very much respect the Reviewer's comment and suggestion, we will be reporting the results in our follow up manuscript(s) as we are conducting that study in one of ongoing projects.

2. Furthermore, the authors should benchmark better against GHs (i.e. the used NEB O-glycosidase) on the synthetic glycopeptides and glycoproteins (beyond just fetuin, which does not contain a mucin-domain). Without doing this, it is difficult to appreciate the utility of POGases as a tool for glycobiology… which is pivotal to demonstrating the advantage of POGases over currently used GHs.

Response: The Reviewer's comment-2 is continuous of Comment-1. We agree with the Reviewer's comment on benchmarking POGase AS with NEB O-glycosidase in order to demonstrate the advantage of POGase. Importantly, we have demonstrated that the POGase has the advantage over currently used GH101(Figure 5 and Figure 6) using defined glycopeptides as well as glycoproteins, fetuin and 2 biotherapeutics. This work is focusing on identifying and characterizing the previously unidentified enzyme, as opposed to developing biotechnology for O-glycomics which is currently ongoing in the follow up study. We acknowledge the POGase AS is not a perfect tool yet, but this work is a great step forward in developing/generating a true universal POGase with efficient releases of all sialylated O-glycans in future which will promote the functional O-glycomics. Similar to our response to this Reviewer's comment-1, we respect the Reviewer's comment, we are conducting the relevant studies will be reporting soon.

Overall, this work is not about biotechnology or tool development. As we discussed, the POGase AS is not a perfect tool yet, however, this work is a step forward to potentially developing a feasible biotechnology/tool for O-glycomics. We believe the audiences will appreciate the study, and more importantly, it will also impact numerous scientific disciplines including glycobiology, biotechnology, microbiology, immunology, and medicine. We respect the Reviewer's comment, we are conducting the study and will be reporting in a different manuscript soon.

3. The glycan array data is overinterpreted: there's no direct data demonstrating cellular adhesion. Accessory domains in some O-glycoproteases/mucinases have been suggested (and even observed in Glycobiology 2021, 31, 385) to be involved in secondary recognition of larger mucin substrates. That the N-terminal domain and the C-terminal domain constructs lack enzymatic activity (Extended Figure 10) suggests that the overall fold or cooperativity between the two domains has been lost. Regardless, it's difficult to ascertain if these or some other mechanism is at play without doing larger structural study.

Response: Thank you for the comment. We agree with your critical point that the full-length of POGase acts at enzymatic activity toward sialylated O-glycans by cooperatively folding or coordinating the N- and C-terminal domains. To get direct evidence to support that the POGase is a potential adhesin, we conducted the cell-binding study. As shown in the new Fig. 9 in our revised manuscript, we found that both full-length POGase and POGase-CD can bind to the human cell line HEK293 cells, and enhanced binding weas observed after desilaylation of cells. More importantly, both full-length POGase and POGase-CD only had moderate binding to the HEK293 cells-CKO cells which only express truncated O-glycans, the Tn antigens. The POGase-ND did not show any binding to either cell lines. As HEK293 cells do not express type-I

chains on N-glycans, our cell-binding data demonstrated that POGase does bind to the HEK293 cells in an O-glycan dependent manner.

Modeling Critiques:

The modeling work follows standard protocols to generate model structures (AlphaFold2) and perform docking (AutoDock). The generated enzyme structure appears to have undergone quality control. The docking, by contrast, has not gone through sufficient quality control/analysis.

The current docked poses (Figure 7 & Extended Data Figure 9) have some questionable results, including several instances where (1) the anomeric carbon of the GalNAc residue is distant from the DDE catalytic triad and (2) there are high-energy cis amide bonds in the NAc moiety and ring flips with some sugars.

Response: We respect the Reviewer's critics.

1) Considering the intramolecular energy of the disaccharide glycan bonds does not change the result suggesting that while the glycans are strained in their confirmation, the intermolecular contacts between the glycan and enzyme active site can compensate for some of the nonideal torsion angels, as would be expected in the context of induced fit.
2) Considering the intramolecular energy of the sialylated glycan bonds does change the docking result. After considering the intramolecular energy of the sialylated glycan the bound-pose more closely resembles the bound-pose of non-sialylated glycans. This again suggests coordination between the intermolecular active-site contacts of flexibility of the glycan substrate.
    a. These studies were redone using both Autodock Vina and Vina Carb to account for carbohydrate specific intramolecular forces. The original manuscript used only Autodock Vina as the docking algorithm. The anomeric carbon in the results isn't any further from the catalytic triad than the experimental structure that the reviewer referenced.
    b. The reviewer mentions the anomeric carbon of 5a56, which was used to align to the docked poses presented in the revised manuscript, showing the poses are not significantly farther than the T antigen (Core 1) analogue observed in 5a56. The alignment also shows similar positioning of the docked glycans to the T antigen analogue in 5a56 which informed the presented docked positions shown in the study. Other poses where the anomeric carbon was facing away from the DDE catalytic triad were not presented in this version of the manuscript.

Additionally, the GalNAc and Gal moieties are oriented with their nonpolar faces away from the WW motif (preventing CH-pi stacking), which is unexpected given (a) what is known about GalNAc recognition (Acc. Chem. Res. 2023, 56, 548) and – more importantly – (b) poses of glycans observed in X-ray structures of other GH101-containing enzymes (J. Biol. Chem. 2015, 290, P25657). As such, I doubt that any of the docked poses reflect the actual binding pose of glycans.

Response: We respect the Reviewer's comment. Interaction through π-stacking is not considered in the molecular force field of AutoDock Vina or the alternative VinaCarb. However, because the enzyme is in its apo form, like one of the conformations of the GH101 domain in 5a56, the tryptophans that act as an active site lid are not expected to contribute to the docked pose. MD trajectories long enough to allow for the apo to substrate bound conformational shift of the enzyme were outside the scope of this study. We have other studies looking more into the specific binding properties of these enzymes which we will not be discussing in this article. Specifically, we are planning studies specifically aimed at analyzing the

contribution of the WW domain in activity and binding by site direct mutagenesis. Models and MD simulation that take into account CH-π stacking will be more relevant for those studies.

The authors should redo the docking studies, ideally using an enzyme-T antigen cocrystal structure from the aforementioned JBC article as a guide to initially place the T-antigen into active site of the modeled enzyme. This can be docked using AutoDock. From there, the other core structures can be easily generated: modifying sugars of the docked T-antigen and/or “grafting” other crystallographic sugars (which can be searched using http://glycanstructure.org/fragment-db) onto the T-antigen core and generating a small library in which the new sugars are allowed to adopt different conformations. Each conformer library can then be docked using AutoDock as before and you can get improved results. This process will help ensure that docking begins with low energy ligand conformers placed in approximately the “right” place within the pocket. Yes, it introduces bias into the system, but it is at least grounded in prior results and allows for more meaningful data interpretation.

Response: We thank the Reviewer’s suggestion. The original manuscript used only Autodock Vina as the docking algorithm. The docking studies were redone using both Autodock Vina and Vina Carb to account for carbohydrate specific intramolecular forces. Poses were compared to the 5a56 pose of the T antigen (Core 1) analogue in to direct the pose of the presented glycan poses in our revised manuscript, which was not done for the original manuscript.

As it stands, the docking doesn’t add much to the story of the paper. I would recommend the authors (1) compare their docked structures to the JBC crystal structures to better understand why the POGases permit larger and more diverse glycans than GHs and (2) possibly dock glycans into modeled structures of POGases that are less active or more selective to better understand these differences, as the authors suggest that these enzymes could be engineered for better results.

Response: We thank the Reviewer’s comment and take the recommendation. In the revised manuscript, an analysis was added to hypothesize why the structure of POGase AS allows for coordination of sialylated glycans, while others don’t (Changes in motif 1 and changes to hydrophobic core interactions)**.** Furthermore, we conducted an evolutionary coupling analysis to add more to the structural/computational story so further studies could be designed going forward and to begin to make sense of the structure of POGase AS.

Finally – partially because the coloring between Figure 7a and 7b are inconsistent – it remains unclear how the conserved, ligand-binding residues of the F5/8 type C domains (which can probably be determined using UniProt) spatially relate to the active site. Again, it is very well possible that the noncatalytic domains participate cooperatively in binding larger, densely glycosylated substrates like mucins rather than engaging in cellular adhesion, as the authors have implied.

Response: We thank the Reviewer’s comment. We have included a movie to better show the distribution of each domain in our model POGase AS, specific studies on the F5/8 type C domain our outside the scope of the current study, but again more studies targeted toward specific domains and experimental structures are planned or in progress. Also coloring was made more consistent between figures in this revised manuscript. Interestingly, other POGase structures have slightly different docking results probably due to the less conserved F5/8 type C and GalBD domains. Here, the focus of the docking for this study was

POGase AS, other model POGases will be used for future studies. Exact change in this revised manuscript also include: the revised manuscript only uses POGase AS a model as the original manuscript used multiple POGase models to confirm positions. Other POGase models will be used in future studies.

We have incorporated these major changes/revisions into the corresponding Results and Discussion, as well as the Methods Sections in the revised manuscript. *Because of the <u>extensive revision and changes</u> made in the computational structural docking studies across Results, Discussion and Methods Sections in the revised manuscript, we could not include every change in these point-by-point responses to the Reviewer 4's comments, but a summary is provided below.* As discussed in the manuscript, the predicted structures through modeling will be either confirmed or corrected through either cryo-EM or Crystallography experiments in future studies as much more needs to be studied and learned.

A summary of the specific changes from the original manuscript is included:

- Comparing Autodock Vina and Vina Carb for docking studies
- Inclusion of evolutionary coupling analysis to better understand changes in POGase AS structure from 5a56
- Alignment of 5a56 to the docked POGase:glycan structures for better verification of poses
- Clarification in the methods section for conducting the docking studies
- Changes to the presentation of figures (color of the background, coloring of the molecules)
- Inclusion of movies to help aid in spatial understanding of the models
- Exclusion of other POGase models to give this manuscript more focus (and to save for other studies)
- Additions to results and discussion section to reflect the updated study.

Point-by-Point Response (In purple) to Reviewer Comments (Manuscript_NCOMMS-24-20598B)

Reviewer #1 (Remarks to the Author):

Though the information added is rich, the general grammar in these new sections is still lacking. A detailed proof read is still needed before this manuscript can be accepted.

Response: We really appreciate the Reviewer's critics and revision of our manuscript. We have carefully revised our manuscript with a detailed proofread.

Minor comments:

The sentence on page 11, line 320 needs to be reworked, please find my suggested sentence structure "Consistent with Figure 6a, three major unreduced O-glycans were identified in Fetuin (mono-sialylCore 1: m/z 879; di-sialylCore 1: m/z 1240; and di-sialylCore 2: m/z 1689) (Figure 6c top panel). However, in abatacept and etanercept, only two O-glycan species were identified from β-elimination reactions, which included mono-sialylCore 1 (m/z 879) and di-sialylCore 1(m/z 1240) (Figure 6d, and 6e top panel), which is consistent with the observations made in our previous study with the same glycoproteins[30].

Response: We thank the Reviewer's editing. We made the changes as suggested.

Page 11 line 333 "tandem mass spectra were only possible..." were should be was. This issue repeats at line 340.

Response: We made the corrections in both sentences, in Line 333 and Line 340 (in original manuscript).

Page 11 line 344 first sentence needs proof reading.

Response: We revised the first sentence in page 11 for clarity.

Page 16 line 474, HEK293 doesn't need to be defined twice.

Response: We removed "HEK293" in page 16.

The paragraph added on page 23, line 683 seems unusual to me. I appreciate referencing those who helped with piecing together the data and it's meaning, however I am not sure this is a conventional way of representing this.

Response: We appreciate the Reviewer's comment. We thought it is relevant information for the community to be aware.

Figures: Overall the clearness of the figures has significantly increased, however the color scheme used to represent the glycans does not correspond with SNFG, and is honestly quite harsh to look at. Please update colors as defined by SNFG (https://www.ncbi.nlm.nih.gov/glycans/snfg1_5.html table 2).

Response: We apologize the color scheme issue with monosaccharides in our merged manuscript. The color scheme of glycans is consistent with the colors as defined by SNFG in each original figure after individually uploaded, but somehow it was changed after merging of the text with figures.

Reviewer #2 (Remarks to the Author):

The manuscript has improved significantly. Just two points:

Response: We thank the Reviewer for his/her time and effort to review our manuscript.

I'm not sure the second sentence of the intro makes sense.

Response: We revised the sentence for clarity.

Sialic acid should be pinky purple, not blue - maybe this came out wrong in figure 1.

Response: We apologize the color scheme issue with sialic acid. The color of sialic acids in figures was changed due to the merging of the text with figures. The color scheme of glycans is consistent with the colors as defined by SNFG in each original figure after individually uploaded.

Reviewer #3 (Remarks to the Author):

The authors have addressed all of my comments and I have no further suggestions.

Response: We thank the Reviewer for his/her time and effort to review our manuscript.

Reviewer #4 (Remarks to the Author):

In this manuscript, the authors identify a family of enzymes, which they term peptide:O-glycosidases (POGase), that are capable of cleaving substrates containing glycans with an initiating GalNAc residue. Some enzymes were found to cleave substrates with different core structures (1-3), including those modified with sialic acid. The enzymes appear to have broader glycan tolerance than related glycoside hydrolases (GHs) characterized to date. Phylogenetic analysis and modeling were performed to help rationalize this observation. Finally, one enzyme was found to bind specific structures in the glycan-binding array.

A universal O-glycan hydrolase is not known, as most GHs do not act broadly on all O-glycans. POGases with broader substrate tolerance could help fill that void and be of significant use to the field. That being said, I believe the authors need additional/improved studies to better characterize these enzymes and demonstrate such utility/impact before publication is warranted. As my background is primarily with modeling, my comments will largely focus on that portion of the study.

Major Critiques:

1. While the authors emphasize the importance of mucin-type glycosylation, the protein-based substrates (fetuin and etanercept/abatecept) are sparsely glycosylated and/or have a very small mucin-domain. To better understand the substrate tolerance/preference of these enzymes (which the authors admit may have some steric limitations), the authors should characterize activity on a recombinant mucin or larger mucin-domain-containing protein.

Response: We thank the Reviewer's comment and vision on better and deeper understanding of enzymatic mechanism(s) of the POGases. We acknowledge the limitations that the POGase AS has as it cannot efficiently release all sialylated O-glycans, especially for di-sialylated species from glycoproteins. As the Reviewer pointed out, further characterizing activity on a recombinant mucin or larger mucin-domain-containing protein, as well as cell extracts is essential for better understanding of the enzyme which would lead to identify and generate a universal POGase. But we realize that would be a project itself, rather than simple experiments. Therefore, while we very much respect the Reviewer's comment and suggestion, we will be reporting the results in our follow up manuscript(s) as we are conducting that study in one of ongoing projects.

Reviewer Comment #2: While I understand where the authors are coming from, I do feel that this diminishes the impact/utility of the current report.

Response: We thank the Reviewer's understanding. We focused on the identification and characterization of previously unreported enzyme, POGase in this current study. We will continue searching/identifying and engineering a universal POGase with much broader substrate specifies for biotechnology development and O-glycomics studies.

2. Furthermore, the authors should benchmark better against GHs (i.e. the used NEB O-glycosidase) on the synthetic glycopeptides and glycoproteins (beyond just fetuin, which does not contain a mucin-domain). Without doing this, it is difficult to appreciate the utility of POGases as a tool for glycobiology… which is pivotal to demonstrating the advantage of POGases over currently used GHs.

Response: The Reviewer's comment-2 is continuous of Comment-1. We agree with the Reviewer's comment on benchmarking POGase AS with NEB O-glycosidase in order to demonstrate the advantage of POGase. Importantly, we have demonstrated that the POGase has the advantage over currently used GH101(Figure 5 and Figure 6) using defined glycopeptides as well as glycoproteins, fetuin and 2 biotherapeutics. This work is focusing on identifying and characterizing the previously unidentified enzyme, as opposed to developing biotechnology for O-glycomics which is currently ongoing in the follow up study. We acknowledge the POGase AS is not a perfect tool yet, but this work is a great step forward in developing/generating a true universal POGase with efficient releases of all sialylated O-glycans in future which will promote the functional O-glycomics. Similar to our response to this Reviewer's comment-1, we respect the Reviewer's comment, we are conducting the relevant studies will be reporting soon.

Overall, this work is not about biotechnology or tool development. As we discussed, the POGase AS is not a perfect tool yet, however, this work is a step forward to potentially developing a feasible biotechnology/tool for O-glycomics. We believe the audiences will appreciate the study, and more importantly, it will also impact numerous scientific disciplines including glycobiology,

biotechnology, microbiology, immunology, and medicine. We respect the Reviewer's comment, we are conducting the study and will be reporting in a different manuscript soon.

Reviewer Comment #2: Likewise, I do feel that this diminishes the impact/utility of the current report.

Response: As our response for the Reviewer Comment #1-2, we will continue the research to identify and/or engineer a universal POGase for the biotechnology purpose.

3. The glycan array data is overinterpreted: there's no direct data demonstrating cellular adhesion. Accessory domains in some O-glycoproteases/mucinases have been suggested (and even observed in Glycobiology 2021, 31, 385) to be involved in secondary recognition of larger mucin substrates. That the N-terminal domain and the C-terminal domain constructs lack enzymatic activity (Extended Figure 10) suggests that the overall fold or cooperativity between the two domains has been lost. Regardless, it's difficult to ascertain if these or some other mechanism is at play without doing larger structural study.

Response: Thank you for the comment. We agree with your critical point that the full-length of POGase acts at enzymatic activity toward sialylated O-glycans by cooperatively folding or coordinating the N- and C-terminal domains. To get direct evidence to support that the POGase is a potential adhesin, we conducted the cell-binding study. As shown in the new Fig. 9 in our revised manuscript, we found that both full-length POGase and POGase-CD can bind to the human cell line HEK293 cells, and enhanced binding weas observed after desilaylation of cells. More importantly, both full-length POGase and POGase-CD only had moderate binding to the HEK293 cells-CKO cells which only express truncated O-glycans, the Tn antigens. The POGase-ND did not show any binding to either cell lines. As HEK293 cells do not express type-I chains on N-glycans, our cell-binding data demonstrated that POGase does bind to the HEK293 cells in an O-glycan dependent manner.

Reviewer Comment #2: This is a significant improvement to the manuscript that supports the authors' initial statement about cellular adhesion and is consistent with results in the GlycanArray study. My only comment would be to remove the word "massive" in line 481. It is confusing to refer to smaller/truncated glycans like the Tn antigen as comparatively massive.

Response: We thank the Reviewer's positive comment and critical reading. We removed remove the word "massive" in line 481 in our revised manuscript.

Modeling Critiques:

The modeling work follows standard protocols to generate model structures (AlphaFold2) and perform docking (AutoDock). The generated enzyme structure appears to have undergone quality control. The docking, by contrast, has not gone through sufficient quality control/analysis.

The current docked poses (Figure 7 & Extended Data Figure 9) have some questionable results, including several instances where (1) the anomeric carbon of the GalNAc residue is distant from the DDE catalytic triad and (2) there are high-energy cis amide bonds in the NAc moiety and ring flips with some sugars.

Response: We respect the Reviewer's critics. 1) Considering the intramolecular energy of the disaccharide glycan bonds does not change the result suggesting that while the glycans are strained in their confirmation, the intermolecular contacts between the glycan and enzyme active site can compensate for some of the nonideal torsion angels, as would be expected in the context of induced fit.

Reviewer Comment #2: The major issue with prior docking results was not the torsional angles connecting the individual subunits, but it was the ring flipped forms of the individual carbohydrate residues and the cis amide bonds. Those are very high energy and are unlikely to form (much less be compensated for during induced fit). These issues are not present in the updated docked structures.

Response: We thank the Reviewer for providing critics and insight into our previous docking results.

2) Considering the intramolecular energy of the sialylated glycan bonds does change the docking result. After considering the intramolecular energy of the sialylated glycan the bound-pose more closely resembles the bound-pose of non-sialylated glycans. This again suggests coordination between the intermolecular active-site contacts of flexibility of the glycan substrate.

a. These studies were redone using both Autodock Vina and Vina Carb to account for carbohydrate specific intramolecular forces. The original manuscript used only Autodock Vina as the docking algorithm. The anomeric carbon in the results isn't any further from the catalytic triad than the experimental structure that the reviewer referenced.

b. The reviewer mentions the anomeric carbon of 5a56, which was used to align to the docked poses presented in the revised manuscript, showing the poses are not significantly farther than the T antigen (Core 1) analogue observed in 5a56. The alignment also shows similar positioning of the docked glycans to the T antigen analogue in 5a56 which informed the presented docked positions shown in the study. Other poses where the anomeric carbon was facing away from the DDE catalytic triad were not presented in this version of the manuscript.

Reviewer Comment #2: The current results are significantly improved and are in better agreement with prior experimental data. I would have liked to see the authors include results from all the original 5 glycans and correlate the binding efficiency with GlycanArray results to really bring things full circle.

Response: We appreciate the Reviewer's comment. While Core 1, α2,3SialylCire 1, and Core 3 glycans were able to be docked into the catalytic site of POGase AS with high confidence, the other two glycans, α2,6sialylCore 1 and Core 2 structures could not be docked into POGase AS to generate consistent results. Core 2 could be docked into one of POGase orthologs, POGase KS (#37), as stated in the manuscript. These also strongly suggest that other domains of POGases also participate in the substrate recognition and binding. A statement has been added connecting the activity (kd/km) assays, hypothesizing that the activity is likely sue to the switch to the POGase motifs. Due to the nature of the study, these are the only solid hypotheses we feel ready to discuss

and have further studies designed to better explore how POGase structure allows for a wider substrate pool. Nevertheless, much more needs to be studied and learned in future, will be reported. We respectively ask the Reviewer to agree with us. Finally, while the docking data is focusing on the GH101 catalytic domain at the N-terminus of the POGase AS, the GlycanArray data is generated from the intact protein, POGase TB and its C-terminal Domain.

Additionally, the GalNAc and Gal moieties are oriented with their nonpolar faces away from the WW motif (preventing CH-pi stacking), which is unexpected given (a) what is known about GalNAc recognition (Acc. Chem. Res. 2023, 56, 548) and – more importantly – (b) poses of glycans observed in X-ray structures of other GH101-containing enzymes (J. Biol. Chem. 2015, 290, P25657). As such, I doubt that any of the docked poses reflect the actual binding pose of glycans.

Response: We respect the Reviewer's comment. Interaction through π-stacking is not considered in the molecular force field of AutoDock Vina or the alternative VinaCarb. However, because the enzyme is in its apo form, like one of the conformations of the GH101 domain in 5a56, the tryptophans that act as an active site lid are not expected to contribute to the docked pose. MD trajectories long enough to allow for the apo to substrate bound conformational shift of the enzyme were outside the scope of this study. We have other studies looking more into the specific binding properties of these enzymes which we will not be discussing in this article. Specifically, we are planning studies specifically aimed at analyzing the contribution of the WW domain in activity and binding by site direct mutagenesis. Models and MD simulation that take into account CH-π stacking will be more relevant for those studies.

Reviewer Comment #2: I agree that full on MD and larger structural study are not necessary for the current study. That the new results are consistent with prior experimental data is sufficient.

Response: We thank the Reviewer for agreeing with us.

The authors should redo the docking studies, ideally using an enzyme-T antigen cocrystal structure from the aforementioned JBC article as a guide to initially place the T-antigen into active site of the modeled enzyme. This can be docked using AutoDock. From there, the other core structures can be easily generated: modifying sugars of the docked T-antigen and/or "grafting" other crystallographic sugars (which can be searched using http://glycanstructure.org/fragment-db) onto the T-antigen core and generating a small library in which the new sugars are allowed to adopt different conformations. Each conformer library can then be docked using AutoDock as before and you can get improved results. This process will help ensure that docking begins with low energy ligand conformers placed in approximately the "right" place within the pocket. Yes, it introduces bias into the system, but it is at least grounded in prior results and allows for more meaningful data interpretation.

Response: We thank the Reviewer's suggestion. The original manuscript used only Autodock Vina as the docking algorithm. The docking studies were redone using both Autodock Vina and Vina Carb to account for carbohydrate specific intramolecular forces. Poses were compared to the 5a56 pose of the T antigen (Core 1) analogue in to direct the pose of the presented glycan poses in our revised manuscript, which was not done for the original manuscript.

Reviewer Comment #2: As stated before, the current results are significantly improved and are in better agreement with prior experimental data.

Response: We appreciate the Reviewer's time and effort for improving our manuscript.

As it stands, the docking doesn't add much to the story of the paper. I would recommend the authors (1) compare their docked structures to the JBC crystal structures to better understand why the POGases permit larger and more diverse glycans than GHs and (2) possibly dock glycans into modeled structures of POGases that are less active or more selective to better understand these differences, as the authors suggest that these enzymes could be engineered for better results.

Response: We thank the Reviewer's comment and take the recommendation. In the revised manuscript, an analysis was added to hypothesize why the structure of POGase AS allows for coordination of sialylated glycans, while others don't (Changes in motif 1 and changes to hydrophobic core interactions). Furthermore, we conducted an evolutionary coupling analysis to add more to the structural/computational story so further studies could be designed going forward and to begin to make sense of the structure of POGase AS.

Reviewer Comment #2: This is fine. I look forward to more in-depth structural analysis in the future.

Response: We will conduct more in-dept structural analysis/study and would like to have the opportunity to collaborate the Reviewer in future.

Finally – partially because the coloring between Figure 7a and 7b are inconsistent – it remains unclear how the conserved, ligand-binding residues of the F5/8 type C domains (which can probably be determined using UniProt) spatially relate to the active site. Again, it is very well possible that the noncatalytic domains participate cooperatively in binding larger, densely glycosylated substrates like mucins rather than engaging in cellular adhesion, as the authors have implied.

Response: We thank the Reviewer's comment. We have included a movie to better show the distribution of each domain in our model POGase AS, specific studies on the F5/8 type C domain our outside the scope of the current study, but again more studies targeted toward specific domains and experimental structures are planned or in progress. Also coloring was made more consistent between figures in this revised manuscript. Interestingly, other POGase structures have slightly different docking results probably due to the less conserved F5/8 type C and GalBD domains. Here, the focus of the docking for this study was POGase AS, other model POGases will be used for future studies. Exact change in this revised manuscript also include: the revised manuscript only uses POGase AS a model as the original manuscript used multiple POGase models to confirm positions. Other POGase models will be used in future studies.

Reviewer Comment #2: The adhesion assay has helped to clear some of this up, and the video also helps to make everything clearer to see.

Response: We thank the Reviewer for providing insight into our new data added.

We have incorporated these major changes/revisions into the corresponding Results and Discussion, as well as the Methods Sections in the revised manuscript. Because of the extensive revision and changes made in the computational structural docking studies across Results, Discussion and Methods Sections in the revised manuscript, we could not include every change in these point-by-point responses to the Reviewer 4's comments, but a summary is provided below. As discussed in the manuscript, the predicted structures through modeling will be either confirmed or corrected through either cryo-EM or Crystallography experiments in future studies as much more needs to be studied and learned.

A summary of the specific changes from the original manuscript is included:

- Comparing Autodock Vina and Vina Carb for docking studies.
- Inclusion of evolutionary coupling analysis to better understand changes in POGase AS structure from 5a56.
- Alignment of 5a56 to the docked POGase:glycan structures for better verification of poses.
- Clarification in the methods section for conducting the docking studies.
- Changes to the presentation of figures (color of the background, coloring of the molecules).
- Inclusion of movies to help aid in spatial understanding of the models.
- Exclusion of other POGase models to give this manuscript more focus (and to save for other studies)
- Additions to results and discussion section to reflect the updated study.

Reviewer #4 (Remarks to the Author):

In this manuscript, the authors identify a family of enzymes, which they term peptide:O-glycosidases (POGase), that are capable of cleaving substrates containing glycans with an initiating GalNAc residue. Some enzymes were found to cleave substrates with different core structures (1-3), including those modified with sialic acid. The enzymes appear to have broader glycan tolerance than related glycoside hydrolases (GHs) characterized to date. Phylogenetic analysis and modeling were performed to help rationalize this observation. Finally, one enzyme was found to bind specific structures in the glycan-binding array.

A universal O-glycan hydrolase is not known, as most GHs do not act broadly on all O-glycans. POGases with broader substrate tolerance could help fill that void and be of significant use to the field. That being said, I believe the authors need additional/improved studies to better characterize these enzymes and demonstrate such utility/impact before publication is warranted. As my background is primarily with modeling, my comments will largely focus on that portion of the study.

Major Critiques:

1. While the authors emphasize the importance of mucin-type glycosylation, the protein-based substrates (fetuin and etanercept/abatecept) are sparsely glycosylated and/or have a very small mucin-domain. To better understand the substrate tolerance/preference of these enzymes (which the authors admit may have some steric limitations), the authors should characterize activity on a recombinant mucin or larger mucin-domain-containing protein.

Response: We thank the Reviewer's comment and vision on better and deeper understanding of enzymatic mechanism(s) of the POGases. We acknowledge the limitations that the POGase AS has as it cannot efficiently release all sialylated O-glycans, especially for di-sialylated species from glycoproteins. As the Reviewer pointed out, further characterizing activity on a recombinant mucin or larger mucin-domain-containing protein, as well as cell extracts is essential for better understanding of the enzyme which would lead to identify and generate a universal POGase. But we realize that would be a project itself, rather than simple experiments. Therefore, while we very much respect the Reviewer's comment and suggestion, we will be reporting the results in our follow up manuscript(s) as we are conducting that study in one of ongoing projects.

Reviewer Comment #2: While I understand where the authors are coming from, I do feel that this diminishes the impact/utility of the current report.

2. Furthermore, the authors should benchmark better against GHs (i.e. the used NEB O-glycosidase) on the synthetic glycopeptides and glycoproteins (beyond just fetuin, which does not contain a mucin-domain). Without doing this, it is difficult to appreciate the utility of POGases as a tool for glycobiology… which is pivotal to demonstrating the advantage of POGases over currently used GHs.

Response: The Reviewer's comment-2 is continuous of Comment-1. We agree with the Reviewer's comment on benchmarking POGase AS with NEB O-glycosidase in order to demonstrate the advantage of POGase. Importantly, we have demonstrated that the POGase has the advantage over currently used GH101(Figure 5 and Figure 6) using defined glycopeptides as well as glycoproteins, fetuin and 2 biotherapeutics. This work is focusing on identifying and characterizing the previously unidentified enzyme, as opposed to developing biotechnology for O-glycomics which is currently ongoing in the follow up study. We acknowledge the POGase AS is not a perfect tool yet, but this work is a great step forward in developing/generating a true universal POGase with efficient releases of all sialylated O-glycans in future

which will promote the functional O-glycomics. Similar to our response to this Reviewer's comment-1, we respect the Reviewer's comment, we are conducting the relevant studies will be reporting soon.

Overall, this work is not about biotechnology or tool development. As we discussed, the POGase AS is not a perfect tool yet, however, this work is a step forward to potentially developing a feasible biotechnology/tool for O-glycomics. We believe the audiences will appreciate the study, and more importantly, it will also impact numerous scientific disciplines including glycobiology, biotechnology, microbiology, immunology, and medicine. We respect the Reviewer's comment, we are conducting the study and will be reporting in a different manuscript soon.

Reviewer Comment #2: Likewise, I do feel that this diminishes the impact/utility of the current report.

3. The glycan array data is overinterpreted: there's no direct data demonstrating cellular adhesion. Accessory domains in some O-glycoproteases/mucinases have been suggested (and even observed in Glycobiology 2021, 31, 385) to be involved in secondary recognition of larger mucin substrates. That the N-terminal domain and the C-terminal domain constructs lack enzymatic activity (Extended Figure 10) suggests that the overall fold or cooperativity between the two domains has been lost. Regardless, it's difficult to ascertain if these or some other mechanism is at play without doing larger structural study.

Response: Thank you for the comment. We agree with your critical point that the full-length of POGase acts at enzymatic activity toward sialylated O-glycans by cooperatively folding or coordinating the N- and C-terminal domains. To get direct evidence to support that the POGase is a potential adhesin, we conducted the cell-binding study. As shown in the new Fig. 9 in our revised manuscript, we found that both full-length POGase and POGase-CD can bind to the human cell line HEK293 cells, and enhanced binding weas observed after desilaylation of cells. More importantly, both full-length POGase and POGase-CD only had moderate binding to the HEK293 cells-CKO cells which only express truncated O-glycans, the Tn antigens. The POGase-ND did not show any binding to either cell lines. As HEK293 cells do not express type-I chains on N-glycans, our cell-binding data demonstrated that POGase does bind to the HEK293 cells in an O-glycan dependent manner.

Reviewer Comment #2: This is a significant improvement to the manuscript that supports the authors' initial statement about cellular adhesion and is consistent with results in the GlycanArray study. My only comment would be to remove the word "massive" in line 481. It is confusing to refer to smaller/truncated glycans like the Tn antigen as comparatively massive.

Modeling Critiques:

The modeling work follows standard protocols to generate model structures (AlphaFold2) and perform docking (AutoDock). The generated enzyme structure appears to have undergone quality control. The docking, by contrast, has not gone through sufficient quality control/analysis.

The current docked poses (Figure 7 & Extended Data Figure 9) have some questionable results, including several instances where (1) the anomeric carbon of the GalNAc residue is distant from the DDE catalytic triad and (2) there are high-energy cis amide bonds in the NAc moiety and ring flips with some sugars.

Response: We respect the Reviewer's critics.

1) Considering the intramolecular energy of the disaccharide glycan bonds does not change the result suggesting that while the glycans are strained in their confirmation, the intermolecular contacts between the glycan and enzyme active site can compensate for some of the nonideal torsion angels, as would be expected in the context of induced fit.

Reviewer Comment #2: The major issue with prior docking results was not the torsional angles connecting the individual subunits, but it was the ring flipped forms of the individual carbohydrate residues and the *cis* amide bonds. Those are *very* high energy and are unlikely to form (much less be compensated for during induced fit). These issues are not present in the updated docked structures.

2) Considering the intramolecular energy of the sialylated glycan bonds does change the docking result. After considering the intramolecular energy of the sialylated glycan the bound-pose more closely resembles the bound-pose of non-sialylated glycans. This again suggests coordination between the intermolecular active-site contacts of flexibility of the glycan substrate.
    a. These studies were redone using both Autodock Vina and Vina Carb to account for carbohydrate specific intramolecular forces. The original manuscript used only Autodock Vina as the docking algorithm. The anomeric carbon in the results isn't any further from the catalytic triad than the experimental structure that the reviewer referenced.
    b. The reviewer mentions the anomeric carbon of 5a56, which was used to align to the docked poses presented in the revised manuscript, showing the poses are not significantly farther than the T antigen (Core 1) analogue observed in 5a56. The alignment also shows similar positioning of the docked glycans to the T antigen analogue in 5a56 which informed the presented docked positions shown in the study. Other poses where the anomeric carbon was facing away from the DDE catalytic triad were not presented in this version of the manuscript.

Reviewer Comment #2: The current results are significantly improved and are in better agreement with prior experimental data. I would have liked to see the authors include results from all the original 5 glycans and correlate the binding efficiency with GlycanArray results to really bring things full circle.

Additionally, the GalNAc and Gal moieties are oriented with their nonpolar faces away from the WW motif (preventing CH-pi stacking), which is unexpected given (a) what is known about GalNAc recognition (Acc. Chem. Res. 2023, 56, 548) and – more importantly – (b) poses of glycans observed in X-ray structures of other GH101-containing enzymes (J. Biol. Chem. 2015, 290, P25657). As such, I doubt that any of the docked poses reflect the actual binding pose of glycans.

Response: We respect the Reviewer's comment. Interaction through π-stacking is not considered in the molecular force field of AutoDock Vina or the alternative VinaCarb. However, because the enzyme is in its apo form, like one of the conformations of the GH101 domain in 5a56, the tryptophans that act as an active site lid are not expected to contribute to the docked pose. MD trajectories long enough to allow for the apo to substrate bound conformational shift of the enzyme were outside the scope of this study. We have other studies looking more into the specific binding properties of these enzymes which we will not be discussing in this article. Specifically, we are planning studies specifically aimed at analyzing the contribution of the WW domain in activity and binding by site direct mutagenesis. Models and MD simulation that take into account CH-π stacking will be more relevant for those studies.

Reviewer Comment #2: I agree that full on MD and larger structural study are not necessary for the current study. That the new results are consistent with prior experimental data is sufficient.

The authors should redo the docking studies, ideally using an enzyme-T antigen cocrystal structure from the aforementioned JBC article as a guide to initially place the T-antigen into active site of the modeled

enzyme. This can be docked using AutoDock. From there, the other core structures can be easily generated: modifying sugars of the docked T-antigen and/or "grafting" other crystallographic sugars (which can be searched using http://glycanstructure.org/fragment-db) onto the T-antigen core and generating a small library in which the new sugars are allowed to adopt different conformations. Each conformer library can then be docked using AutoDock as before and you can get improved results. This process will help ensure that docking begins with low energy ligand conformers placed in approximately the "right" place within the pocket. Yes, it introduces bias into the system, but it is at least grounded in prior results and allows for more meaningful data interpretation.

Response: We thank the Reviewer's suggestion. The original manuscript used only Autodock Vina as the docking algorithm. The docking studies were redone using both Autodock Vina and Vina Carb to account for carbohydrate specific intramolecular forces. Poses were compared to the 5a56 pose of the T antigen (Core 1) analogue in to direct the pose of the presented glycan poses in our revised manuscript, which was not done for the original manuscript.

Reviewer Comment #2: As stated before, the current results are significantly improved and are in better agreement with prior experimental data.

As it stands, the docking doesn't add much to the story of the paper. I would recommend the authors (1) compare their docked structures to the JBC crystal structures to better understand why the POGases permit larger and more diverse glycans than GHs and (2) possibly dock glycans into modeled structures of POGases that are less active or more selective to better understand these differences, as the authors suggest that these enzymes could be engineered for better results.

Response: We thank the Reviewer's comment and take the recommendation. In the revised manuscript, an analysis was added to hypothesize why the structure of POGase AS allows for coordination of sialylated glycans, while others don't (Changes in motif 1 and changes to hydrophobic core interactions)**.** Furthermore, we conducted an evolutionary coupling analysis to add more to the structural/computational story so further studies could be designed going forward and to begin to make sense of the structure of POGase AS.

Reviewer Comment #2: This is fine. I look forward to more in-depth structural analysis in the future.

Finally – partially because the coloring between Figure 7a and 7b are inconsistent – it remains unclear how the conserved, ligand-binding residues of the F5/8 type C domains (which can probably be determined using UniProt) spatially relate to the active site. Again, it is very well possible that the noncatalytic domains participate cooperatively in binding larger, densely glycosylated substrates like mucins rather than engaging in cellular adhesion, as the authors have implied.

Response: We thank the Reviewer's comment. We have included a movie to better show the distribution of each domain in our model POGase AS, specific studies on the F5/8 type C domain our outside the scope of the current study, but again more studies targeted toward specific domains and experimental structures are planned or in progress. Also coloring was made more consistent between figures in this revised manuscript. Interestingly, other POGase structures have slightly different docking results probably due to the less conserved F5/8 type C and GalBD domains. Here, the focus of the docking for this study was POGase AS, other model POGases will be used for future studies. Exact change in this revised manuscript also include: the revised manuscript only uses POGase AS a model as the original manuscript used multiple POGase models to confirm positions. Other POGase models will be used in future studies.

Reviewer Comment #2: The adhesion assay has helped to clear some of this up, and the video also helps to make everything clearer to see.

We have incorporated these major changes/revisions into the corresponding Results and Discussion, as well as the Methods Sections in the revised manuscript. *Because of the extensive revision and changes made in the computational structural docking studies across Results, Discussion and Methods Sections in the revised manuscript, we could not include every change in these point-by-point responses to the Reviewer 4's comments, but a summary is provided below.* As discussed in the manuscript, the predicted structures through modeling will be either confirmed or corrected through either cryo-EM or Crystallography experiments in future studies as much more needs to be studied and learned.

A summary of the specific changes from the original manuscript is included:

- Comparing Autodock Vina and Vina Carb for docking studies
- Inclusion of evolutionary coupling analysis to better understand changes in POGase AS structure from 5a56
- Alignment of 5a56 to the docked POGase:glycan structures for better verification of poses
- Clarification in the methods section for conducting the docking studies
- Changes to the presentation of figures (color of the background, coloring of the molecules)
- Inclusion of movies to help aid in spatial understanding of the models
- Exclusion of other POGase models to give this manuscript more focus (and to save for other studies)
- Additions to results and discussion section to reflect the updated study.
